# Non-responsiveness to cardioprotection by ischaemic preconditioning in Ossabaw minipigs with genetic predisposition to, but without the phenotype of the metabolic syndrome

**DOI:** 10.1007/s00395-022-00965-0

**Published:** 2022-11-11

**Authors:** Petra Kleinbongard, Helmut Raphael Lieder, Andreas Skyschally, Mouhamad Alloosh, Axel Gödecke, Sven Rahmann, Michael Sturek, Gerd Heusch

**Affiliations:** 1 Institute for Pathophysiology, West German Heart and Vascular Center, University of Essen Medical School, University of Duisburg-Essen, Hufelandstr. 55, 45147 Essen, Germany; 2 Department of Anatomy, Cell Biology, and Physiology, Indiana University School of Medicine, Indianapolis, USA; 3 Institute for Cardiovascular Physiology, University Hospital and Heinrich-Heine University, Düsseldorf, Germany; 4 Algorithmic Bioinformatics, Center for Bioinformatics and Department of Computer Science, Saarland University, Saarbrücken, Germany

**Keywords:** Cardioprotection, Genetic predisposition, Ischaemic preconditioning, Metabolic syndrome, Myocardial ischaemia

## Abstract

The translation of successful preclinical and clinical proof-of-concept studies on cardioprotection to the benefit of patients with reperfused acute myocardial infarction has been difficult so far. This difficulty has been attributed to confounders which patients with myocardial infarction typically have but experimental animals usually not have. The metabolic syndrome is a typical confounder. We hypothesised that there may also be a genuine non-responsiveness to cardioprotection and used Ossabaw minipigs which have the genetic predisposition to develop a diet-induced metabolic syndrome, but before they had developed the diseased phenotype. Using a prospective study design, a reperfused acute myocardial infarction was induced in 62 lean Ossabaw minipigs by 60 min coronary occlusion and 180 min reperfusion. Ischaemic preconditioning by 3 cycles of 5 min coronary occlusion and 10 min reperfusion was used as cardioprotective intervention. Ossabaw minipigs were stratified for their single nucleotide polymorphism as homozygous for valine (V/V) or isoleucine (I/I)) in the γ-subunit of adenosine monophosphate-activated protein kinase. Endpoints were infarct size and area of no-reflow. Infarct size (V/V: 54 ± 8, I/I: 54 ± 13% of area at risk, respectively) was not reduced by ischaemic preconditioning (V/V: 55 ± 11, I/I: 46 ± 11%) nor was the area of no-reflow (V/V: 57 ± 18, I/I: 49 ± 21 vs. V/V: 57 ± 21, I/I: 47 ± 21% of infarct size). Bioinformatic comparison of the Ossabaw genome to that of *Sus scrofa* and Göttingen minipigs identified differences in clusters of genes encoding mitochondrial and inflammatory proteins, including the janus kinase (JAK)—signal transducer and activator of transcription (STAT) pathway. The phosphorylation of STAT3 at early reperfusion was not increased by ischaemic preconditioning, different from the established STAT3 activation by cardioprotective interventions in other pig strains. Ossabaw pigs have not only the genetic predisposition to develop a metabolic syndrome but also are not amenable to cardioprotection by ischaemic preconditioning.

**Supplementary Information:**

The online version contains supplementary material available at 10.1007/s00395-022-00965-0.

## Introduction

There is still a need for cardioprotection beyond that by early reperfusion. Interventional reperfusion is increasingly used in patients with acute myocardial infarction, but 1-year mortality in a large contemporary registry was still around 14% in 2017 [86], and the morbidity from post-infarct heart failure is even increasing [29]. Injury to both, cardiomyocytes, i.e., infarct size [82], and the coronary microcirculation, i.e., microvascular obstruction [11], contribute to patients’ prognosis. Whereas encouraging preclinical and clinical proof-of-concept studies demonstrated reduced infarct size by mechanical and pharmacological cardioprotection strategies [27], their translation to better clinical outcome in phase III trials in patients with acute myocardial infarction has been largely disappointing [1, 4, 25]. Such failure of translation has primarily been attributed to co-morbidities and co-medications of patients with acute myocardial infarction which may impair cardioprotective signalling [26]. In contrast, animals in most preclinical studies have no such co-morbidities and co-medications [4, 13, 39]. The metabolic syndrome is a typical co-morbidity which predisposes patients to acute myocardial infarction and comprises several features which impair cardioprotective signalling, including glucose intolerance and hyperinsulinaemia that progress to type 2 diabetes, obesity, hypertension, and dyslipidaemia. We originally aimed to better understand the mechanisms through which a metabolic syndrome interferes with cardioprotection and possibly overcome this obstacle. We, therefore, used our established clinically relevant model of coronary occlusion/reperfusion [32, 37, 40, 49, 78] in minipigs of a particular strain (Ossabaw) which develop a metabolic syndrome upon a hypercaloric, atherogenic diet and consequently coronary atherosclerosis and occasional myocardial infarction [83, 88, 94]. To induce cardioprotection we used ischaemic preconditioning (IPC) [56] which is unequivocally the strongest and most robust stimulus for cardioprotection, but failed to reduce infarct size when using our established protocol of IPC [14] in preliminary experiments.

We then hypothesised that there may also be a **primordial** [7], genetically determined non-responsiveness to cardioprotection and designed a prospective study to look at infarct size reduction by IPC. The Ossabaw minipigs used for these experiments had the major genotype associated with the thrifty phenotype and develop a robust metabolic syndrome [83]. These Ossabaw minipigs have a single nucleotide polymorphism encoding for isoleucine (I) rather than valine (V) in the 199^th^ position of the γ-subunit of adenosine monophosphate-activated protein kinase (AMPK) [50, 83]. AMPK is an important sensor of the metabolic and energetic state in myocardial ischaemia [51, 92] and causally involved in cardioprotection by IPC and by atorvastatin in mice [53, 57]. We, therefore, studied Ossabaw minipigs homozygous for both γ-subunit AMPK genotypes (V/V and I/I). For IPC, we now used the consensus protocol of the Consortium for preclinicAl assESsment of cARdioprotective therapies (CAESAR) [35].

Again, both genotypes had no infarct size reduction with IPC. We performed DNA sequencing in the Ossabaw minipigs and a bioinformatic comparison to the genomes of *Sus scrofa* and Göttingen minipigs [67] to identify genes that might explain the lack of cardioprotection in Ossabaw minipigs. Major differences in genes encoding janus kinase (JAK)—signal transducer and activator of transcription (STAT) signalling were identified and were consistent with a lack of increased STAT3 phosphorylation at early reperfusion in the Ossabaw minipigs which we have demonstrated before as causal for cardioprotection by ischaemic conditioning in Göttingen minipigs [14, 31, 78]. For comparison, we used contemporary and published data [40] from our Göttingen minipigs.

## Methods

The authors declare that all supporting data of the present study are available in the article and its Online supplement. Experiments were performed between September 2017 and June 2021. Unless otherwise specified, materials were obtained from Sigma-Aldrich (Deisenhofen, Germany).

### Experimental preparation

Ossabaw minipigs (females, age 15 ± 3 months, and castrated males, aged 16 ± 3 months) were purchased from CorVus Biomedical, LLC (Crawfordsville, Indiana USA). Male piglet castration was performed at around 4 weeks of age under local anaesthesia with lidocaine and/or bupivacaine. Ossabaw minipigs were housed in tiled rooms (~ 2 m^2^/pig) with straw-bedding at 12/12 h light/dark cycles, fed with standard chow (500 g twice/day, #V4133. Ssniff, Soest, Germany) and had ad libitum access to water. The phenotypic features of Ossabaw minipigs, including body weight, baseline haemodynamics and serum glucose and lipids are displayed in Table 1 and compared to those of Göttingen minipigs in Table 2. Pigs were sedated with flunitrazepam (i.m.: 0.8 mg/kg). Anaesthesia was induced with etomidate (i.v.: 0.3 mg/kg, Piramal Critical Care B.V., Hypnomidat; Voorschoten, The Netherlands) and sufentanil (i.v.: 1 µg/kg, Sufentanil-hameln, hameln pharma gmbh, Hameln, Germany). Anaesthesia was maintained with isoflurane (2%, TEVA, Eastbourne, United Kingdom) during artificial ventilation with room air. Muscle relaxation during electrosurgery was induced with a single bolus of rocuronium (i.v. 1.2 mg/kg, B. Braun, Melsungen, Germany). This anaesthetic regimen is identical to that used in our institution for patients undergoing surgical coronary revascularization [87]. The pigs were placed on a heated table and covered with drapes to keep oesophageal temperature between 37.0 and 39.0 °C. ECG-lead II was continuously recorded using a single-channel, calibrated amplifier. A midline cervical incision was performed. After tracheotomy for mechanical ventilation, the left jugular vein was cannulated for volume replacement and intravenous drug administration, and the right common carotid artery was cannulated to measure arterial pressure. The heart was exposed by a left lateral thoracotomy and instrumented with a micromanometer (DPT-6000, Codan-PVB, Forsting, Germany) in the left ventricle to measure left ventricular pressure and a Teflon catheter in the left atrium for the injection of coloured microspheres. The distal aortic arch was cannulated to withdraw a reference sample for regional blood flow measurement. The left anterior descending coronary artery (LAD) was dissected and prepared distal to its second diagonal branch for later coronary occlusion. Blood cell counts and laboratory parameters were determined from arterial blood samples taken within 60 min after induction of anaesthesia (Tables 1 and 2).

**Table 1:** Phenotypic features of Ossabaw minipigs with V/V and I/I genotypes undergoing ischaemia/reperfusion without or with prior ischaemic preconditioning

	Ischaemia/reperfusion		Ischaemic preconditioning + Ischaemia/reperfusion		
	V/V (*n* = 15)	I/I (*n* = 16)	V/V (*n* = 16)	I/I (*n* = 15)	
Body weight [kg]	50 ± 9	52 ± 6	50 ± 6	50 ± 6	
Left ventricular pressure [mmHg]	86 ± 8	93 ± 8	85 ± 6	88 ± 7	Main effect genotype p = 0.0117
Erythrocytes [10^6^/mm^3^]	6.0 ± 0.9	6.2 ± 0.8	5.9 ± 0.8	5.9 ± 1.0	
Leukocytes [10^3^/mm^3^]	8.2 ± 1.7	8.6 ± 1.6	8.7 ± 2.2	8.7 ± 1.9	
Platelets [10^3^/mm^3^]	308 ± 51	253 ± 47	314 ± 73	234 ± 40	Main effect genotype p < 0.0001
Na^+^ [mmol/L]	141 ± 2	143 ± 8	142 ± 2	141 ± 1	
K^+^ [mmol/L]	3.6 ± 0.2	3.7 ± 0.4	3.7 ± 0.3	3.8 ± 0.4	
Glucose [mg/dL]	103 ± 27	83 ± 19	89 ± 25	87 ± 28	
Cholesterol [mg/dL]	68 ± 8	76 ± 15	67 ± 8	75 ± 12	Main effect genotype p = 0.0069
HDL [mg/dL]	27 ± 5	29 ± 8	28 ± 4	28 ± 5	
LDL [mg/dL]	29 ± 6	35 ± 9	27 ± 5	34 ± 7	Main effect genotype p = 0.0002
Triglycerides [mg/dL]	42 ± 14	39 ± 13	45 ± 11	40 ± 11	
hs-CRP [mg/dL]	< 0.02	< 0.02	< 0.02	< 0.02	
AST [U/L]	47 ± 9	50 ± 8	46 ± 10	47 ± 12	
GPT [U/L]	71 ± 15	90 ± 20	69 ± 16	71 ± 23	Main effect protocol *p* = 0.0481 Main effect genotype p = 0.0332
Creatinine [mg/dL]	1.10 ± 0.17	1.02 ± 0.23	1.14 ± 0.20	1.00 ± 0.23	

Baseline variables and laboratory parameters of Ossabaw minipigs with a V/V genotype (pigs with homozygous valine in the γ-subunit of the adenosine monophosphate-activated protein kinase) and I/I genotype (pigs with homozygous isoleucine in the γ-subunit of the adenosine monophosphate-activated protein kinase) undergoing myocardial ischaemia/reperfusion without or with prior ischaemic preconditioning. Two-way (genotype, protocol) analysis of variance with least square means tests

*AST* aspartate aminotransferase; *GPT* glutamate pyruvate transaminase; *HDL* high-density lipoproteins; *hs-CRP* high sensitivity C-reactive protein; *LDL* low-density lipoproteins

**Table 2:** Phenotypic features of female and castrated male Ossabaw and Göttingen minipigs undergoing ischaemia/reperfusion without or with prior ischaemic preconditioning

	Ossabaw minipigs				Göttingen minipigs			
	Ischaemia/reperfusion		Ischaemic preconditioning + Ischaemia/reperfusion		Ischaemia/reperfusion		Ischaemic preconditioning + Ischaemia/reperfusion	
	Female (*n* = 15)	Castrated male (n = 16)	Female (*n* = 15)	Castrated male (n = 16)	Female (*n* = 5)	Castrated male (n = 4)	Female (*n* = 5)	Castrated male (n = 4)
Age [months]	16 ± 3	16 ± 3	15 ± 2	15 ± 2	14 ± 1	15 ± 1	14 ± 1	14 ± 1
Body weight [kg]	49 ± 9	53 ± 9	52 ± 6	48 ± 6	44 ± 5	45 ± 5	47 ± 4	40 ± 13
Left ventricular pressure [mmHg]	89 ± 9	90 ± 9	87 ± 7	86 ± 7	84 ± 5	79 ± 10	83 ± 7	94 ± 16
Erythrocytes [10^6^/mm^3^]	6.1 ± 1.0	6.0 ± 1.0	6.0 ± 0.8	5.8 ± 0.8	5.4 ± 0.7	5.3 ± 0.7	5.1 ± 0.1	5.5 ± 0.7
Leukocytes [10^3^/mm^3^]	8.8 ± 1.6	8.1 ± 1.6	8.4 ± 1.6	9.0 ± 1.9	5.4 ± 0.9	4.9 ± 0.7	7.0 ± 2.2	6.0 ± 0.7
Platelets [10^3^/mm^3^]	293 ± 47	267 ± 47	284 ± 61	267 ± 75	426 ± 117	502 ± 67	417 ± 90	453 ± 71
Na^+^ [mmol/L]	143 ± 9	142 ± 9	142 ± 2	141 ± 2	139 ± 2	139 ± 2	141 ± 4	142 ± 3
K^+^ [mmol/L]	3.7 ± 0.3	3.6 ± 0.3	3.7 ± 0.4	3.7 ± 0.4	3.6 ± 0.3	3.9 ± 0.5	3.6 ± 0.4	3.8 ± 0.5
Glucose [mg/dL]	97 ± 24	89 ± 24	94 ± 26	82 ± 26	85 ± 24	82 ± 17	102 ± 28	115 ± 38
Cholesterol [mg/dL]	73 ± 17	71 ± 17	72 ± 9	70 ± 14	85 ± 11	68 ± 16	89 ± 15	86 ± 16
HDL [mg/dL]	28 ± 6	28 ± 6	28 ± 7	27 ± 5	40 ± 7	33 ± 7	38 ± 10	42 ± 7
LDL [mg/dL]	32 ± 11	32 ± 11	30 ± 3	30 ± 8	29 ± 7	23 ± 8	37 ± 14	33 ± 11
Triglycerides [mg/dL]	42 ± 15	39 ± 15	45 ± 12	40 ± 14	51 ± 13	40 ± 5	54 ± 14	64 ± 27
hs-CRP [mg/dL]	< 0.02	< 0.02	< 0.02	< 0.02	< 0.02	< 0.02	< 0.02	< 0.02
AST [U/L]	46 ± 6	51 ± 6	45 ± 10	48 ± 13	23 ± 6	26 ± 3	24 ± 5	29 ± 7
GPT [U/L]	81 ± 23	78 ± 23	70 ± 17	71 ± 18	42 ± 9	57 ± 9	44 ± 8	56 ± 13
Creatinine [mg/dL]	1.08 ± 0.21	1.05 ± 0.21	1.07 ± 0.19	1.07 ± 0.27	0.62 ± 0.13	0.70 ± 0.29	0.67 ± 0.05	0.83 ± 0.07

Baseline variables and laboratory parameters of female and castrated male Ossabaw minipigs and female and castrated male Göttingen minipigs undergoing myocardial ischaemia/reperfusion without or with prior ischaemic preconditioning

*AST* aspartate aminotransferase; *GPT* glutamate pyruvate transaminase; *HDL* high-density lipoproteins; *hs-CRP* high sensitivity C-reactive protein; *LDL* low-density lipoproteins

### Protocols

#### Ischaemia/reperfusion (I/R)

When baseline heart rate was below 95/min, left atrial pacing at 100/min was performed with bipolar rectangular pulses of 2 ms duration and 2–4 V amplitude using an analogue stimulus isolator (Model 2200; A-M Systems/ADInstruments, Dunedin, New Zealand). At baseline, systemic haemodynamics and regional myocardial blood flow were measured. Myocardial drill biopsies (2–10 mg) were taken from the designated area at risk, immediately snap-frozen in liquid nitrogen and stored at − 80 °C for later Western blot analysis. Thereafter, unfractionated heparin (i.v.: 500 I.E. LEO Pharma, Neu-Isenburg, Germany) was administered and the LAD occluded distal to its second diagonal branch using a microvascular clamp (TKL-1, Biover AG, Hergiswill, Switzerland). The heparin administration was repeated at 30 and 55 min coronary occlusion. After 5 and 55 min coronary occlusion, systemic haemodynamics and regional myocardial blood flow were measured again. Biopsies were taken at 55 min coronary occlusion. Reperfusion was induced after 60 min coronary occlusion by quick removal of the vascular clamp and visually confirmed by the reappearance of red colour on the surface of the reperfused myocardium. Systemic haemodynamics were again measured at 10, 60, 120 min and 180 min reperfusion and regional myocardial blood flow at 10 and 180 min reperfusion. Additional myocardial biopsies were taken at 10 min reperfusion. The experiment was terminated after 180 min reperfusion by intracardiac injection of 20 ml potassium chloride (1 mol/L). Ventricular fibrillation during ischaemia or reperfusion, as identified from the continuous lead II ECG recording, was immediately terminated by intra-thoracic defibrillation (up to 50 Ws); 6/4 ms biphasic pulse; Zoll R Series Monitor and Defibrillator, Zoll Medical Cooperation, Chelmsford, MA, USA). We did not use any anti-arrhythmic or inotropic agents, since they might interfere with the infarction process and/or cardioprotection. At the end of the experiment, pigs were euthanised by intracardiac injection of 20 ml potassium chloride (1 mol/L).

#### Ischaemic preconditioning (IPC)

The experimental protocol was identical to that of I/R, except that IPC was induced by three 5 min LAD occlusions separated by 10 min reperfusion before the 60 min LAD occlusion [35]. After the IPC procedure, myocardial biopsies were also taken from the area at risk.

### Regional myocardial blood flow

Coloured microspheres were recovered from transmural myocardial samples taken from the central area at risk by digestion with 4 mol/L KOH and subsequent filtration (8 µm pore size, Pieper Filter, Bad Zwischenahn, Germany). Fluorescent dye was resolved from microspheres and quantified in a spectrophotometer (F-7100, Hitachi High-Tech, Krefeld, Germany). Blood flow was calculated as blood flow per tissue mass [44].

### Area at risk, infarct size and area of no-reflow

Thirty ml of warmed 4% thioflavin-S solution (Morphisto, Frankfurt, Germany) was filtered through a 0.2 µm syringe-filter to remove particulate debris and slowly infused into the left atrium to demarcate non-perfused areas of the left ventricle after 180 min reperfusion [6, 79]. Thereafter, the LAD was re-occluded at the same location as for the index ischaemia, and 5 ml blue dye (Patentblau V, Guerbet GmbH, Sulzbach, Germany) was quickly injected into the left atrium to delineate the area at risk as remaining unstained. The heart was quickly removed from the chest, rinsed with cold saline, and cut into 5 slices perpendicular to the ventricular long axis. The tissue slices were examined under ultraviolet light (340–360 nm, VL-UVA 135.11, Vilber Louramat, Eberhardzell, Germany). Areas without yellow–green fluorescence (thioflavin-S-negative) were encircled by incisions. After documenting the slices using a digital camera, the slice shape, the thioflavin-S-negative areas, and the demarcated area at risk were transferred to a transparent film. Thereafter, infarcted tissue was demarcated by triphenyl-tetrazolium chloride (TTC) staining (1% dissolved in 90 mmol/L sodium phosphate buffer containing 8% dextran, Roth, Karlsruhe, Germany). The TTC-stained slices were again photographed and together with the tissue areas which remained unstained by TTC transferred to the same transparent film which was used to document the area at risk and the areas of no-reflow. Particular care was taken to proper re-align the slices using “landmarks”, such as the position of papillary muscles and the incisions surrounding no-reflow areas. In 6 Ossabaw minipigs, area at risk and infarct size were corrected for unclear border delineation by blue dye or for permanently occluded areas resulting from biopsy lesions or side branch occlusion by use of the microsphere regional flow data; the corrected tissue mass amounted to 1.8 ± 1.8 g.

The transparent films were scanned and analysed using digital planimetry. The following areas were calculated and averaged for both sides of each slice: total area of the left ventricle, the area at risk, the area of TTC-negative tissue (infarcted), the area of thioflavin-S-negative tissue within the infarcted tissue (no-reflow). Using the slice weight for normalization, the tissue masses for the area at risk, the area of no-reflow, and the infarcted area were calculated. In addition, the area at risk was calculated as a fraction of the left ventricle, infarct size was calculated as a fraction of the area at risk, and the area of no-reflow was calculated as a fraction of infarct size.

### AMPK genotyping

#### Genotyping for γ subunit of AMPK

The γ-subunit of AMPK is encoded by the PRKAG3 gene. Primer and restriction enzyme were chosen on the basis of the *Sus scrofa* PRKAG3 gene sequence data base, GenBank, AF214521.1 [55]. Ear biopsies were used to isolate the DNA. Tissue was lysed in a buffer containing: 1 mol/L Tris, 10% sodium dodecyl sulfate, 0.5 mol/L ethylenediamine tetra-acetic acid, 5 mol/L NaCl, pH 7.5 and proteinase K. DNA was isolated by adding an equal volume of phenol:chloroform (both 100%). Samples were vortexed and centrifuged at room temperature for 7 min at 16,060 g. To the upper aqueous phase, a double volume of 100% isopropanol was added. Precipitated DNA was washed twice with addition of 70% ethanol and centrifugation for 6 min at 16,060 g. Dry DNA pellets were resuspended in TE-buffer (AppliChem, Darmstadt, Germany) until solved. DNA was added to Taq polymerase chain reaction (PCR) Master Mix Kit (Quiagen, Hilden, Germany) with dNTP´s (Invitrogen, California, USA) including forward and reverse primer. For PCR, the following PCR primer sequences 5’-ACCAGCAGCCTTAGATCTGGAACAAATGTG-3’ (forward); 5’-TTCCTTCCTCCGCCTGTCCTCTTCTTACTT-3’ (reverse) and 35 cycles for 45 s at 94 °C, 45 s at 65 °C, 60 s at 72 °C were used. PCR products (545 bp) were digested with the restriction enzyme BsaHI (10,000 U/ml, New England Biolabs, Frankfurt, Germany) for 4 h at 37 °C. The digested samples and a DNA ladder (Take5™ 50 bp, highQu, Kraichtal, Germany) ranging from 50 to 1500 bp were loaded on a 4% agarose gel to identify homozygous Ossabaw minipigs with the point mutation resulting in a change from valine 199 to isoleucine in the γ-subunit of AMPK (I/I) with 436 and 109 bp products, homozygous Ossabaw minipigs with valine 199 in the γ-subunit of AMPK (V/V) by 317, 119 and 109 bp products and heterozygous Ossabaw minipigs (I/V) by 436, 317, 119 and 109 bp, respectively. Genotyping for the individual Ossabaw minipigs is displayed in supplemental Fig. 1.

#### DNA extraction

Blood samples were taken from female (*n* = 4, *n* = 2 each of both AMPK γ subunit genotypes) and male (*n* = 4, *n* = 2 of each of both AMPK γ subunit genotypes) Ossabaw minipigs (age 1.2–7.5 years) in the breeding colony of CorVus Biomedical, LLC (Crawfordsville, Indiana USA). Whole blood was stabilised with EDTA as anti-coagulant. Red blood cells were lysed using a hypotonic buffer containing ammonium chloride to remove red blood cells with minimal effects on mononuclear cells. After centrifugation (280 g, 4 min, 20 °C) and discarding the supernatant, mononuclear blood cells were separated, and DNA was extracted using the MagAttract kit (MagAttract HMW DNA Handbook 03/2020, Qiagen, Germantown, USA). A chaotropic buffer stabilised and protected the nucleic acids from liberated nucleases. All methods followed the manufacturer’s guidelines (Qiagen, www.qiagen.com). Briefly, magnetic beads were added to the cell lysate and stabilised to enable removal of the supernatant and contaminants. The magnetic beads attenuate shear stress by centrifugation that may fragment the extracted DNA.

### Whole genome sequencing and bioinformatic analysis

**Fig. 1:**
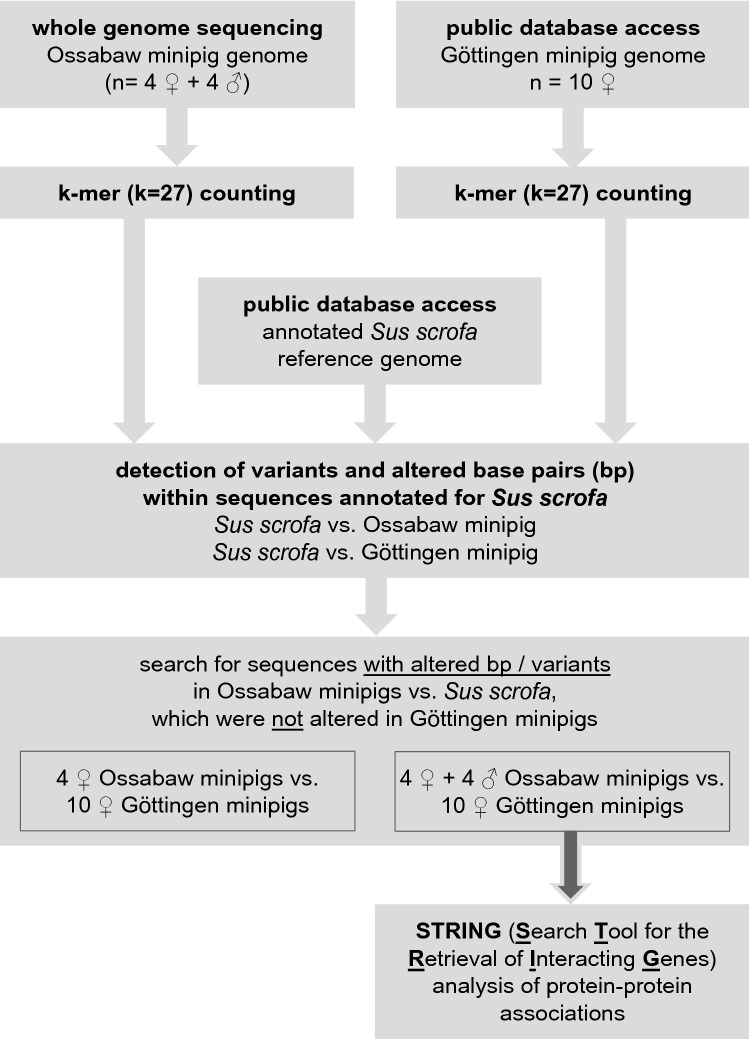
Flowchart of bioinformatic analysis of the genomes of Ossabaw minipigs vs. Göttingen minipigs

The flowchart in Fig. 1 illustrates the process of bioinformatic comparisons between the genomes of Ossabaw minipigs and Göttingen minipigs.

#### Illumina Nextera genome sequencing

The DNA was evaluated for its quantity and quality using the Agilent TapeStation 4200 (Santa Clara, CA, USA) and the Thermo Fisher Qubit Fluorometer 3.0 (Waltham, MA, USA). One hundred ng high quality genomic DNA of each sample was used for library preparation. The quality of high molecular weight DNA was assessed by gel analysis compared to a ladder of 14 different DNA sizes. Briefly, the DNA library was prepared using Illumina Nextera DNA Flex Library Prep Kit (Cat# 20018707; Illumina, Inc. San Diego, CA, USA), following Illumina Nextera DNA Flex Library Prep Reference Guide (Document #1000000025416 v01, April 2018; Illumina, Inc. San Diego, CA, USA). Each resulting library was quantified and its quality accessed by the Qubit and Agilent Bioanalyzer (Santa Clara, CA, USA), and multiple libraries were pooled in equal molarity. The pooled library was then denatured, neutralised and sequenced in 150 bp paired-end read format on a NovaSeq 6000 sequencer (Illumina, Inc. San Diego, CA, USA). More than 300 million paired reads per sample were generated for 30–40-fold coverage, and 91% of the sequencing reads reached Q30 (99.9% base call accuracy). A Phred quality score (Q score) was used to measure the quality of sequencing (80 × for DNA) [48]. The raw data for these 8 Ossabaw minipigs are available from the national center for biotechnology information (NCBI) Sequence Read Archive/BioProject number PRJNA837405 (accession numbers: SAMN28202074–SAMN28202081).

Whole genome data from 10 female Göttingen minipigs were obtained from the public European Nucleotide Archive (project number PRJEB27654, accession number ERR2744277–ERR2744286). These Göttingen minipig genomes were sequenced on the Illumina platform (paired-end 2 × 100 bp) with an average coverage of approximately 15-fold (i.e., 38.7 sequenced Gbp for an assumed genome size of 2.58 Gbp) [67]. Details on the sequenced genome of the 8 Ossabaw minipigs and the used genome data of the Göttingen minipigs are given in supplemental Table 1.

#### Genomic k-mer content of Ossabaw minipigs and Göttingen minipigs

K-mer-based methods are well-suited to discover genetic variants associated with phenotypes [66, 81]. We report the genomic content of Ossabaw minipigs and Göttingen minipigs by the set of their constituting k-mers (DNA pieces of length k). Throughout this study, we used *k* = 27 as it provides sufficient specificity (most k-mers which do not occur in repetitive regions anyway are unique in the genome). We assume that a k-mer belongs to an individual genome if we see it sufficiently many times in the sequenced reads of the individual. The exact definition of sufficiently many times depends on the average coverage of the genome and is shown in the table as the threshold T (see supplemental Table 1). The threshold is obtained by analysing the k-mer histogram which plots the number of k-mers (*y*-axis) with a specific occurrence count c against c (*x*-axis) [80]. We assume that a k-mer belongs to the strain of Göttingen minipigs or Ossabaw minipigs, respectively, if it belongs to sufficiently many individual genomes of that strain (at least 3 out of 10 Göttingen minipigs, at least 3 out of 8 Ossabaw minipigs). We did the same analysis only for female pigs (at least 3 out of 10 female Göttingen minipigs, at least 3 out of 4 female Ossabaw minipigs). To determine these sets of k-mers, we counted all k-mers in all samples using the fastpork k-mer counter (developed by SR: https://gitlab.com/rahmannlab/fastpork, Project ID: 36185346, https://doi.org/10.5281/zenodo.6551661) and verified the numbers with a different k-mer counter (KMC3 [41]). We then aggregated the k-mer counts of all samples in one large table, where we tracked to how many individual Göttingen minipigs and Ossabaw minipigs each k-mer belongs. In other words, each k-mer now has a pair of counters (i,j), where i (between 0 and 8 [or 4, for females only]) represents the number of Ossabaw minipigs to which it belongs and j (between 0 and 10) represents the number of Göttingen minipigs to which it belongs. The total number of k-mers in the aggregated table is 2 782 273 127 (for all 8 Ossabaw minipigs, or 2 759 727 695 for the 4 female Ossabaw minipigs). Summary statistics of the aggregated table (number of k-mers with specific (i,j) values) are given in supplemental Table 2 (for all 8 Ossabaw minipig samples and separately for the 4 female Ossabaw minipig samples).

#### Comparison of Ossabaw minipig and Göttingen minipig genomes to Sus scrofa reference

The Ossabaw minipig and the Göttingen minipig genomes are not available as annotated reference genomes. Thus, we used the only publicly available representative reference genome assembly from NCBI for *Sus scrofa*, submitted by “The Swine Genome Sequencing Consortium” [72] (Sscrofa11.1; GenBank: GCA_000003025.6; RefSeq: GCF_000003025.6).

For the present analysis, we hypothesised that the observed phenotype, specifically here “infarct size reduction by IPC” is connected to genes that are present in the *Sus scrofa* reference and unchanged in the Göttingen minipig genome, but heavily altered in the Ossabaw minipig genome. This assumption is supported by the fact that IPC reduced infarct size in all published studies in pigs so far, no matter which strain of pigs and which algorithm of IPC [12, 16, 35, 40, 42, 46, 52, 58, 64, 71, 73, 75–77].

Therefore, we scanned along the *Sus scrofa* reference and queried each k-mer whether it also belongs to the Ossabaw minipig genome or to the Göttingen minipig genome. In the positive case, we marked the corresponding k base pairs of the *Sus scrofa* reference as present in the corresponding other strain(s). Base pairs which were not marked after scanning all positions of the reference were called altered base pairs. Altered base pairs can correspond to deleted or changed base pairs. A block of consecutive altered base pairs is called a variant (e.g., a deletion of several base pairs, a substitution of several consecutive base pairs, supplemental Fig. 2). Another type of variant, not discovered by altered base pairs, but by reference k-mers which do not belong to the genomic content of one of the other strains, are insertions (supplemental Fig. 2). We kept track of the genomic positions of all altered base pairs and all variants in the *Sus scrofa* reference in comparison with both the Göttingen minipig and the Ossabaw minipig genome separately (supplemental Table 3).

Using the *Sus scrofa* genome annotation, we counted, for each gene separately, the number of variants and the number of altered base pairs in the total genome, the sequence from first to last coding base, and the gene’s exons for both the Ossabaw minipig and the Göttingen minipig genome (supplemental Table 3). These analyses were performed for the comparison of the 8 (4 female, 4 male) Ossabaw vs. the 10 female Göttingen minipigs and separately for the 4 female Ossabaw vs. the 10 female Göttingen minipigs (supplemental Table 3).

#### Protein–protein associations

To analyse protein–protein associations of protein coding sequences altered in the Ossabaw minipig but unchanged in the Göttingen minipig genome in comparison with the *Sus scrofa* reference, the Search Tool for the Retrieval of Interacting Genes (STRING, http://string-db.org/, version 11.5) [85] was used. Protein–protein association networks were generated using the “Multiple Proteins by Names/Identifiers” tool, the organism was set as “Homo Sapiens”. The “Interaction score” was set as medium confidence (0.400). Protein–protein association networks were visualised with the STRING tool, and networks were exported to an excel data sheet (supplemental “STRING-protein–protein associations”).

### Western blot analysis

Snap-frozen myocardial drill biopsies were homogenised in 100.0 mmol/L tris(hydroxymethyl)aminomethane with 2% sodium dodecyl sulfate, (w/v; SERVA Electrophoresis GmbH, Heidelberg, Germany), heated to 70 °C for 5 min and centrifuged at 16,000 g for 10 min. The protein lysate containing supernatant was stored at -80 °C in aliquots to prevent freeze and thaw cycles [38]. In preliminary experiments, the combined linear range had been determined for each analysed protein and its phosphorylated form according to the manufacturer’s recommendation when using fluorescence signals [63], using series of 8, 12, 16, 20, 24, 28, and 32 µg protein lysate. The determined protein quantities within the linear range (32 µg for protein kinase B (AKT), phosphatase and tensin homolog (PTEN) and signal transducer and activator of transcription (STAT)3 and 24 µg for adenosine monophosphate-activated protein kinase (AMPK) and extracellular signal-regulated kinase (ERK)) were used for Western blotting. Protein lysates from 61 of the 62 Ossabaw minipigs were loaded randomly in blocks per pig on the gels, and each protein lysate was loaded onto different gels (2 × 32 µg and 1 × 24 µg). One Ossabaw minipig (V/V female with IPC) was excluded from analysis, because only one biopsy was available. A reference sample, prepared from a pool of 30 randomly selected protein lysates, was loaded onto each gel for subsequent data normalization.

Protein lysate aliquots were electrophoretically separated on precast stain-free 7.5% sodium dodecyl sulphate polyacrylamide electrophoresis gels (BioRad, Hercules, USA). Total protein was visualised by an ultraviolet light-induced fluorescence reaction of protein–tryptophan with tri-halocompounds within the stain-free gels and imaged using the Gel Doc EZ system (BioRad). Proteins were transferred to 0.45 µm low fluorescence polyvinylidene difluoride membranes (Merck, Chemicals GmbH, Darmstadt, Germany) using the Trans-Blot Turbo™ transfer system (BioRad). The membranes were imaged (Gel Doc EZ system) and dried. After reactivation with 100% methanol, membranes were incubated with Revert® (1:15; LI-COR Biosciences, Lincoln, USA) for total protein staining and imaged using the LI-COR Odyssey F_C_ system (LI-COR Biosciences). Membranes were de-stained, cut horizontally, blocked (1:10; EveryBlot® blocking buffer, BioRad) for 5 min at room temperature, and rinsed with tris-buffered saline.

Then membranes for AKT, AMPK, ERK, STAT3 and PTEN were incubated with the respective primary antibodies directed against the phosphorylated forms of the proteins. Membranes were washed 4 × for 5 min with tris-buffered saline containing polyoxyethylene-20-sorbitan monolaurate (TBST) before being incubated with antibodies directed against the total forms. Membranes were again washed for 4 × 5 min with TBST before incubation with the secondary antibodies. Primary antibodies were diluted in TBST containing 5% bovine serum albumin. Secondary antibodies were diluted in EveryBlot® blocking buffer (1:10; BioRad) supplemented with 0.02% sodium dodecyl sulphate. Signals were detected by fluorescence. For the detection of the 160 kDa substrate of the AKT Ser/Thr kinase (AS160, a downstream target of AMPK) and for STAT5, membranes from gels loaded with 32 µg protein lysate, respectively, were incubated with the respective primary antibody directed against the phosphorylated AS160 and STAT5, respectively. After detection of the phosphorylated forms of the proteins, membranes were stripped and re-probed with antibodies against total AS160 and STAT5. Signals were detected by enhanced chemiluminescence (ECL; Pierce Biotechnology, Rockford, USA). Protein phosphorylation sites, ordering numbers of primary and secondary antibodies, antibody working concentrations and antibody incubation times are listed in supplemental Table 4. Fluorescence/luminescence signal intensity of phosphorylated proteins and their respective total proteins was imaged using the LI-COR Odyssey F_C_ system. Detected signals were analysed with the LI-COR Biosciences Empiria® studio software (version 2.1.0.134). All signal intensities were normalised to that of the reference sample on the respective membrane. Then, the signal intensity of each phosphorylated protein was normalised to that of the respective total protein. For the time course analysis, the signals (of total proteins as well as the phosphorylated proteins normalized to their respective total proteins) from each Ossabaw minipig were normalised to the respective baseline signal and expressed as % of baseline.

### Power analysis

Infarct size was the primary endpoint, and we defined type I error α as 0.05 and type II error 1-β as 0.90. Since no data on infarct size reduction in Ossabaw minipigs were available, we used the infarct size data from our own laboratory obtained in male Göttingen minipigs subjected to either I/R (*n* = 23) or an IPC manoeuvre preceding I/R (*n* = 19) with an infarct size reduction by IPC using our established algorithm [14] (21 ± 9 vs. 43 ± 10%) [38]. For the present study, we conservatively assumed an infarct size reduction by IPC in Ossabaw minipigs of half the magnitude of that observed in Göttingen minipigs. This estimated effect size was a compromise between the Null hypothesis, i.e., Ossabaw minipigs do not differ from Göttingen minipigs, and our preliminary observation that Ossabaw pigs were not protected by our established IPC algorithm [14]. The resulting effect size *f* was 0.597 and almost identical to the effect size *f* = 0.594 calculated from the infarct size reduction by IPC in young female Yorkshire pigs reported by the CAESAR consortium (37 ± 19 vs. 58 ± 14%), and we used their IPC algorithm for the present study [35]. For the comparison of 4 groups (I/R vs. IPC in AMPK γ-subunit genotypes V/V vs. I/I) the final a priori calculation of the total cohort size using G-Power 3.1 (University of Düsseldorf, Germany) resulted in *n* = 62 experiments. We did not aim for an additional a priori stratification for sex in the Ossabaw minipigs (females vs. castrated males) but rather chose to perform a pre-specified secondary analysis of I/R vs. IPC in females vs. castrated males. In fact, our contemporary study in Göttingen minipigs revealed no sex-related differences in infarct size [40]. We did not use any inclusion or exclusion criteria and conducted the study until *n* = 62 experiments with infarct data were available. Ossabaw minipigs of a given sex and genotype were block-randomised (in blocks of Ossabaw minipig delivery) for an I/R or IPC protocol, respectively, using sealed envelopes.

### Statistics

Investigators who quantitatively analysed haemodynamics, regional myocardial blood flow, infarct size, area of no-reflow and Western blots were blinded to the pigs´ sex and protocol, respectively. Data were tested for normal distribution using the Shapiro–Wilk test. Data are presented as means ± standard deviations; individual data on infarct size and area of no-reflow are also presented as scatterplots. Data on infarct size, area at risk, and area of no-reflow from Ossabaw minipigs were analysed by two-way analysis of variance (ANOVA; general linear model procedure; main effects protocol and genotype). In a secondary analysis, two-way ANOVA was performed for I/R vs. IPC and female vs. castrated male sex. Haemodynamic, regional myocardial blood flow and AKT, AMPK, AS160, ERK, STAT3, STAT5 and PTEN total protein and protein phosphorylation data were analysed by three-way ANOVA for repeated measures (MIXED procedure; main effects protocol, genotype, and time). When ANOVA indicated a significant main effect or interaction, least square means were used for further analysis of simple effects. Differences were considered significant at the level of *p* < 0.05 (SAS 9.4; Cary, NC, USA).

## Results

Baseline body weight, left ventricular pressure and laboratory data are presented in Tables 1 and 2. There were only minor differences between genotypes (Table 1) and only minor differences to data from the contemporary cohort of female and castrated Göttingen minipigs of the same age (14–15 months) [40] (Table 2). Obviously, the Ossabaw minipigs in the absence of a high-fat diet had no increased body weight or signs of a metabolic syndrome by comparison to Göttingen minipigs of equal age (Table 2). We lost 8 Ossabaw minipigs due to surgical problems (*n* = 5) or intractable ventricular fibrillation (*n* = 3).

### Area at risk, regional myocardial blood flow and systemic haemodynamics

Area at risk was 20–25% of left ventricular myocardium and not different between the 4 groups of Ossabaw minipigs when stratified for their genotypes. Likewise, regional myocardial blood flow at baseline was not different between the 4 groups of Ossabaw minipigs; it was markedly decreased at 5 and 55 min ischaemia, and it recovered with a wide range of intra-individual variation over 180 min reperfusion. Heart rate and left ventricular pressure at baseline were not different between the 4 groups of Ossabaw minipigs; during ischaemia, left ventricular pressure decreased and heart rate increased, without differences between the 4 groups of Ossabaw minipigs. During reperfusion, left ventricular pressure did not fully recover (Table 3). In the pre-specified secondary analysis stratifying the Ossabaw minipigs for their sex, there was also no difference between the 4 groups in area at risk, regional blood flow, heart rate and left ventricular pressure (Table 4).

**Table 3:** Area at risk, baseline temperature, regional myocardial blood flow, heart rate, and left ventricular pressure in V/V and I/I Ossabaw minipigs undergoing ischaemia/reperfusion without and with prior ischaemic preconditioning

			Ischaemia/reperfusion						Ischaemic preconditioning + Ischaemia/reperfusion						
			V/V (*n* = 15)			I/I (*n* = 16)			V/V (*n* = 16)			I/I (*n* = 15)			
AAR [%LV]			24 ± 6			20 ± 5			25 ± 7			24 ± 5			
TEMP [°C]			38.1 ± 0.5			38.1 ± 0.7			38.2 ± 0.5			38.1 ± 0.8			
RMBF [ml/min/g]	*Time p* < *0.0001*														
		BL	0.96 ± 0.22			1.01 ± 0.34			0.96 ± 0.18			1.17 ± 0.49			
		after IPC							1.12 ± 0.36			1.45 ± 0.43			
		I5	0.04 ± 0.02			0.04 ± 0.03			0.05 ± 0.03			0.07 ± 0.04			
		I55	0.03 ± 0.03			0.03 ± 0.02			0.03 ± 0.02			0.03 ± 0.02			
		R10	1.47 ± 0.66			1.15 ± 0.67			1.05 ± 0.62			1.12 ± 0.50			
		R180	0.67 ± 0.30			0.67 ± 0.29			0.63 ± 0.22			0.83 ± 0.59			
HR [1/min]	*Time p* < *0.0001*														Main effect genotype p = 0.0035
		BL	103 ± 6			96 ± 7			99 ± 3			99 ± 8			
		after IPC							103 ± 4			99 ± 4			
		I5	102 ± 4			98 ± 5			103 ± 5			100 ± 4			
		I55	102 ± 8			96 ± 9			103 ± 11			98 ± 7			
		R10	101 ± 8			97 ± 9			102 ± 11			101 ± 5			
		R180	106 ± 8			102 ± 11			108 ± 15			100 ± 10			
LVP [mmHg]	*Time p* < *0.00001*														Main effect protocol p = 0.0391
		BL	86 ± 8			93 ± 8			85 ± 6			88 ± 7			
		after IPC							82 ± 8			84 ± 6			
		I5	79 ± 11			82 ± 12			77 ± 10			79 ± 5			
		I55	77 ± 10			82 ± 15			72 ± 13			73 ± 10			
		R10	75 ± 7			80 ± 13			71 ± 13			76 ± 7			
		R180	75 ± 8			80 ± 11			68 ± 13			74 ± 6			

Area at risk (AAR), temperature before sustained coronary occlusion (TEMP), regional myocardial blood flow (RMBF), heart rate (HR), and left ventricular pressure (LVP) in Ossabaw minipigs with a V/V genotype (pigs with homozygous valine in the γ-subunit of the adenosine monophosphate-activated protein kinase) and I/I genotype (pigs with homozygous isoleucine in the γ-subunit of the adenosine monophosphate-activated protein kinase) undergoing myocardial ischaemia/reperfusion without or with prior ischaemic preconditioning (IPC). Two-way (genotype, protocol) analysis of variance for AAR and temperature; three-way (genotype, protocol, time) analysis of variance for RMBF, HR, and LVP, each with least square means tests

*BL* baseline; *I5* 5 min ischaemia; *I55* 55 min ischaemia; *R10* 10 min reperfusion; *R180* 180 min reperfusion

**Table 4:** Area at risk, baseline temperature, regional myocardial blood flow, heart rate, and left ventricular pressure in female and castrated male Ossabaw minipigs undergoing ischaemia/reperfusion without and with prior ischaemic preconditioning

			Ischaemia/reperfusion						Ischaemic preconditioning + ischaemia/reperfusion						
			Female (*n* = 15)			Castrated male (*n* = 16)			Female (*n* = 15)			Castrated male (*n* = 16)			
AAR [%LV]			24 ± 7			20 ± 4			24 ± 5			24 ± 13			
TEMP [°C]			38.2 ± 0.8			38.0 ± 0.4			38.5 ± 0.6			37.9 ± 0.6			
RMBF [ml/min/g]	*Time p* < *0.0001*														
		BL	1.04 ± 0.32			0.94 ± 0.25			1.17 ± 0.46			0.98 ± 0.39			
		after IPC							1.20 ± 0.46			1.36 ± 0.03			
		I5	0.04 ± 0.03			0.04 ± 0.03			0.05 ± 0.04			0.06 ± 0.02			*p* < *0.001 vs. BL, R10, R180*
		I55	0.03 ± 0.02			0.03 ± 0.02			0.03 ± 0.03			0.03 ± 0.52			*p* < *0.001 vs. BL, R10, R180*
		R10	1.45 ± 0.66			1.18 ± 0.68			1.01 ± 0.58			1.16 ± 0.23			
		R180	0.77 ± 0.28			0.56 ± 0.26			0.74 ± 0.58			0.69 ± 0.23			
HR [1/min]	*Time p* < *0.0001*														
		BL	101 ± 7			98 ± 7			100 ± 4			98 ± 4			
		after IPC							103 ± 5			100 ± 2			
		I5	101 ± 4			98 ± 5			103 ± 6			100 ± 7			
		I55	99 ± 11			99 ± 7			100 ± 12			101 ± 7			
		R10	99 ± 7			99 ± 10			100 ± 10			102 ± 13			
		R180	105 ± 9			103 ± 11			104 ± 10			104 ± 23			
LVP [mmHg]	*Time p* < *0.00001*														*Main effect protocol p* = *0.0397*
		BL	89 ± 9			90 ± 9			87 ± 7			86 ± 7			
		after IPC							83 ± 7			83 ± 10			
		I5	77 ± 12			84 ± 10			77 ± 5			79 ± 12			
		I55	75 ± 13			84 ± 12			70 ± 11			75 ± 10			
		R10	76 ± 10			79 ± 11			70 ± 11			77 ± 9			
		R180	76 ± 8			80 ± 11			68 ± 12			74 ± 10			

Area at risk (AAR), temperature before sustained coronary occlusion (TEMP), regional myocardial blood flow (RMBF), heart rate (HR), and left ventricular pressure (LVP) in female and castrated male Ossabaw minipigs undergoing myocardial ischaemia/reperfusion without or with prior ischaemic preconditioning (IPC). Two-way (sex, protocol) analysis of variance for AAR and temperature; three-way (sex, protocol, time) analysis of variance for RMBF, HR, and LVP, each with least square means tests

*BL* baseline; *I5* 5 min ischaemia; *I55* 55 min ischaemia; *R10* 10 min reperfusion; *R180* 180 min reperfusion

### Infarct size and area of no-reflow

**Fig. 2:**
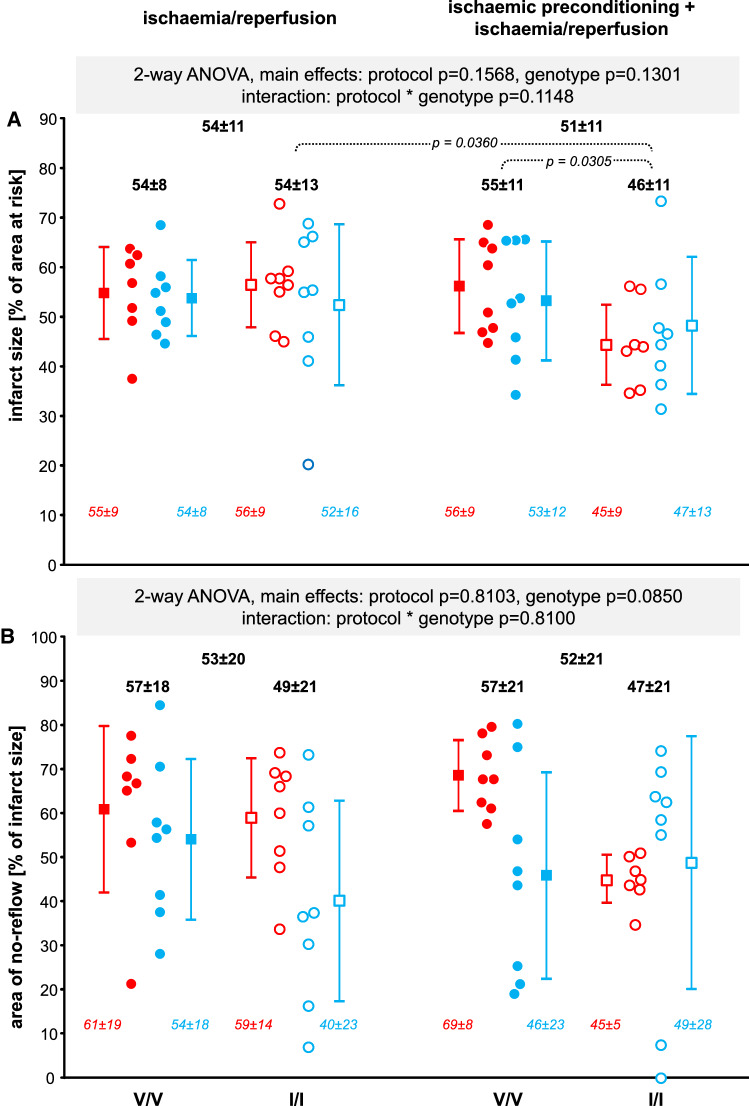
Infarct size (**A**) and area of no-reflow (**B**) following ischaemia/reperfusion without and with prior ischaemic preconditioning in female (red) and castrated male (blue) Ossabaw minipigs—stratified for genotype—with the V/V (filled symbols) and I/I (open symbols) genotypes, respectively. Individual data (circles) and means (squares) with standard deviation. Two-way analysis of variance (general linear model procedure; main effects protocol and genotype) with least square means tests. Overall, there was neither a significant reduction of infarct size nor of area of no-reflow. When overstepping the do-not-test rule of the two-way ANOVA, a reduction of infarct size in the I/I genotype became apparent. V/V: Ossabaw minipigs with homozygous valine in the γ-subunit of the adenosine monophosphate-activated protein kinase; I/I: Ossabaw minipigs with homozygous isoleucine in the γ-subunit of the adenosine monophosphate-activated protein kinase; V/V females with I/R (*n* = 7); V/V castrated males with I/R (*n* = 8); I/I females with I/R (*n* = 8); I/I castrated males with I/R (*n* = 8); V/V females with IPC + I/R (*n* = 8); V/V castrated males with IPC + I/R (*n* = 8); I/I females with IPC + I/R (*n* = 7); I/I castrated males with IPC + I/R (*n* = 8)

**Fig. 3:**
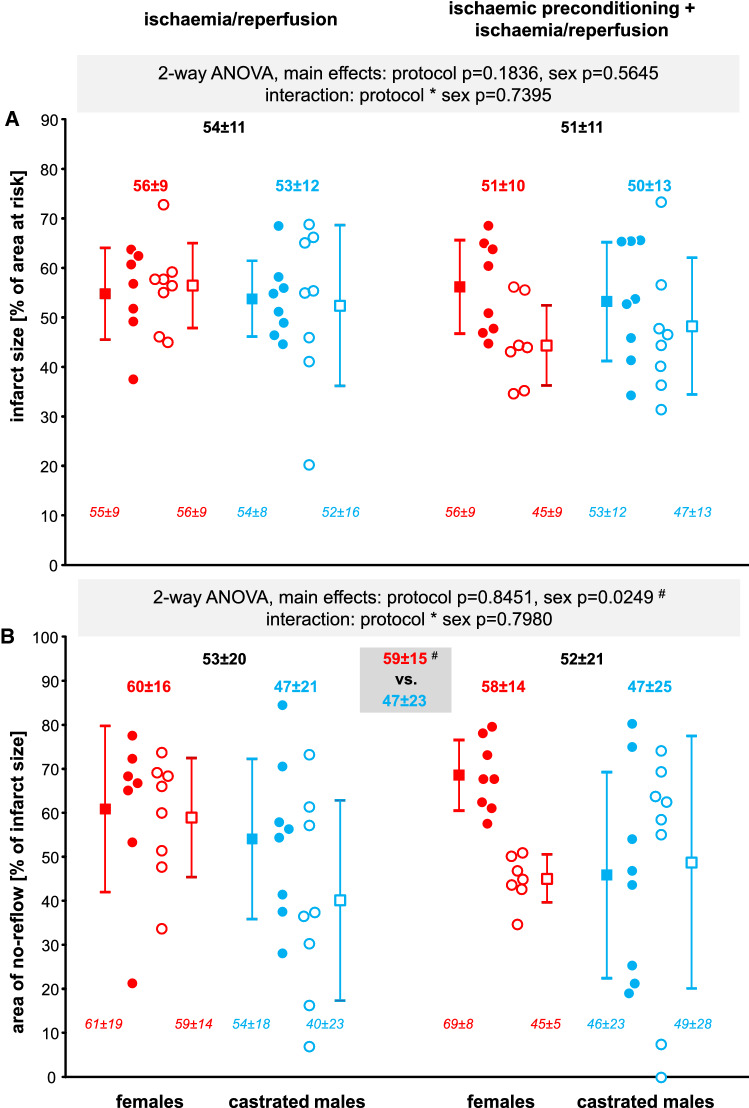
Infarct size (**A**) and area of no-reflow (**B**) following ischaemia/reperfusion without and with prior ischaemic preconditioning in female (red) and castrated male (blue) Ossabaw minipigs—stratified for sex—with the V/V (filled symbols) and I/I (open symbols) genotypes**,** respectively. Individual data (circles) and means (squares) with standard deviation. Two-way analysis of variance (general linear model procedure; main effects protocol and genotype) with least square means tests. **A** There was no significant reduction of infarct size. **B** Area of no-reflow was less in castrated males than in females, ^**#**^ indicates the significant main effect between females vs. castrated males, grey insert. V/V: Ossabaw minipigs with homozygous valine in the γ-subunit of the adenosine monophosphate-activated protein kinase; I/I: Ossabaw minipigs with homozygous isoleucine in the γ-subunit of the adenosine monophosphate-activated protein kinase; females V/V with I/R (*n* = 7); females I/I with I/R (*n* = 8); castrated males V/V with I/R (*n* = 8); castrated males I/I with I/R (*n* = 8); females V/V with IPC + I/R (*n* = 8); females I/I with IPC + I/R (*n* = 7); castrated males V/V with IPC + I/R (*n* = 8); castrated males I/I with IPC + I/R (*n* = 8)

The two-way ANOVA did not reveal any significant effect of protocol, genotype or their interaction on infarct size or no-reflow; thus, infarct size and area of no-reflow were not different between genotypes, and IPC did not reduce infarct size and area of no-reflow (Fig. 2). When overstepping the “do not test”- rule of the two-way ANOVA, IPC reduced infarct size only in the I/I genotype Ossabaw minipigs (Fig. 2A). When looking at individual data points, the infarct size reduction by IPC within the I/I group was more marked in the female than the castrated male Ossabaw minipigs. Even when overstepping the “do not test” -rule, the area of no-reflow was not different between any particular groups (Fig. 2B). In the alternate two-way ANOVA approach comparing the effects of IPC between females and castrated males, there was no significant difference in infarct size. The area of no-reflow was less in castrated males than in females; this difference, however, was not significant between any particular groups (Fig. 3).

### Bioinformatic analysis of differences in the Ossabaw and Göttingen minipig genomes

**Fig. 4:**
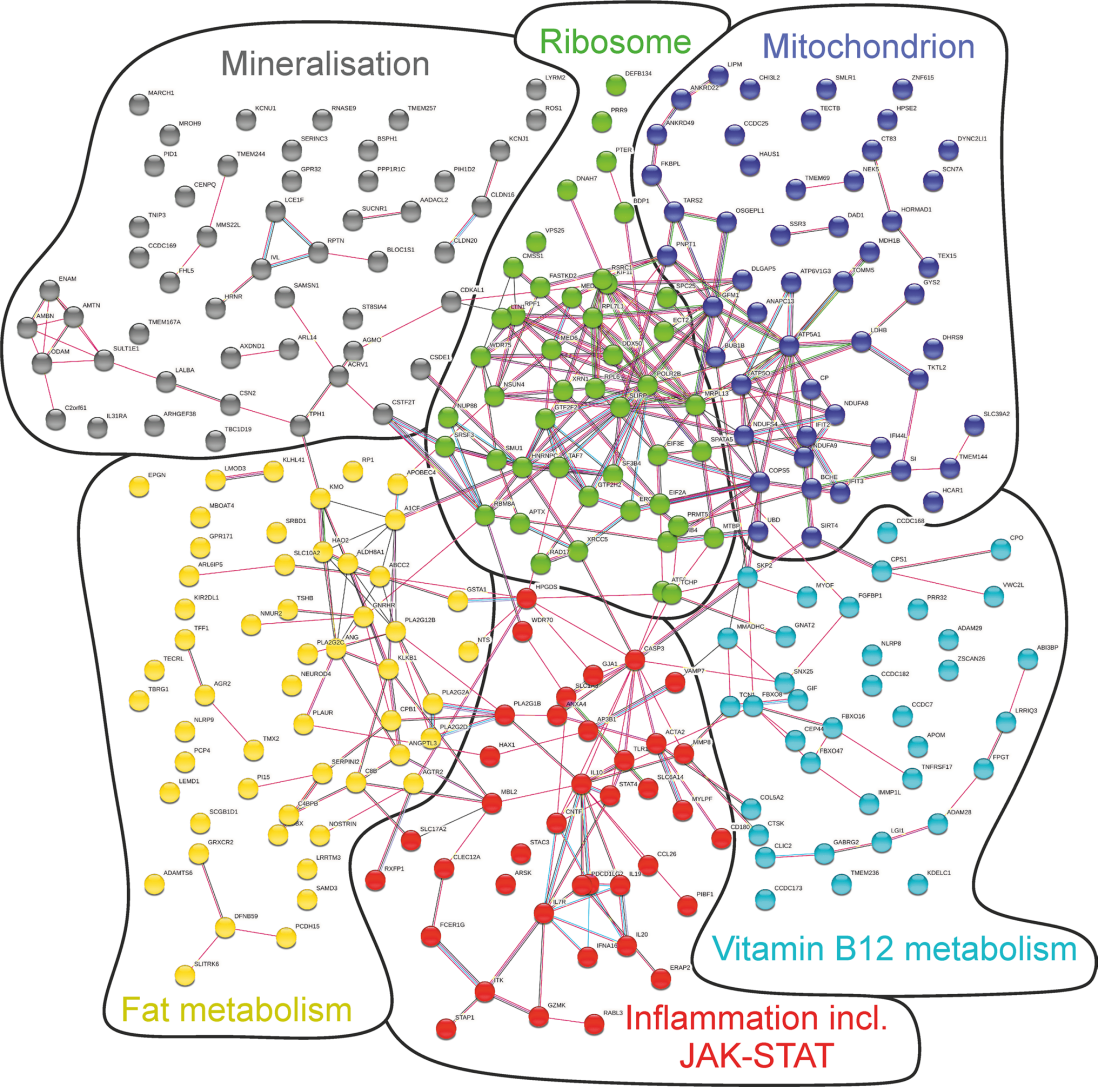
Protein–protein associations of variant protein coding sequences between the Ossabaw minipig and Göttingen minipig strains: The Search Tool for the Retrieval of Interacting Genes (STRING) identified networks for the topic mitochondrion, vitamin B12 metabolism, inflammation including the janus kinase (JAK)—signal transducer and activator of transcription (STAT) pathway, fat metabolism, mineralisation and ribosome. We assigned the cluster designation with reference of the functional enrichments in the STRING network analysis. The matching proteins within the respective networks are listed in the supplemental excel data sheet “STRING–protein–protein associations” column I “matching proteins in your network (labels)”

The exons encoding AMPK γ-subunit (PRKAG3) had by comparison to *Sus scrofa* zero altered bp/Mbp and variants/Mbp in Ossabaw minipigs but several thousand altered bp/Mbp and variants/Mbp in Göttingen minipigs. The same was true for exons encoding for STAT3. The exons encoding ERK1/2 (mitogen-activated protein kinase (3/1) and AKT had by comparison to *Sus scrofa* more altered bp/Mbp in Göttingen minipigs than in Ossabaw minipigs (supplemental Table 5). The exons for the two pig strains differed in 480 altered base pairs and in 294 variants when the 4 female and 4 castrated male Ossabaw minipigs were compared to the 10 female Göttingen minipigs using the *Sus scrofa* annotated genome as reference (supplemental Table 3, supplemental excel data sheet “Altered base pairs and variants in Ossabaw minipigs and Göttingen minipigs”). To contrast potential differences between the Ossabaw minipig and the Göttingen minipig genomes, we focused only on the variants in the exons. The analysis of the 294 variants with STRING referring to the organism “Homo sapiens” identified several protein–protein associations of variant protein coding sequences between the Ossabaw minipig and Göttingen minipig strains, including notably one network on mitochondrial proteins and another one on inflammatory proteins including the JAK–STAT pathway (Fig. 4). In the supplemental excel data sheet “STRING–protein–protein associations” the respective matching proteins (including protein names) of the networks, their strength and false discovery rate are listed.

### Protein kinase phosphorylation

**Fig. 5:**
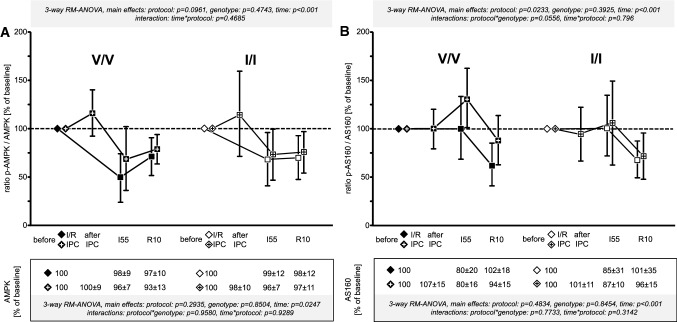
Signal ratio of phosphorylated (p) adenosine monophosphate-activated protein kinase (AMPK) (**A**) and the 160 kDa substrate of the AKT Ser/Thr kinase (AS160) (**B**) vs. total AMPK and AS160 in the V/V genotype (Ossabaw minipigs with homozygous valine in the -subunit of the AMPK; filled symbols) and the I/I genotype (Ossabaw minipigs with homozygous isoleucine in the -subunit of the AMPK; open symbols); quantification of the total protein signals is displayed in the box below the *x*-axis; + within the symbols indicates data from Ossabaw minipigs with ischaemic preconditioning (IPC) prior to ischaemia/reperfusion (I/R); all data were normalised to the baseline samples taken before the I/R or IPC protocol, respectively (indicated by diamonds); symbols are means with standard deviation; I55: 55 min ischaemia, R10: 10 min reperfusion; three-way analysis of variance (MIXED procedure; main effects protocol, genotype, and time) for repeated measures with least square means tests; V/V with I/R (*n* = 15): myocardial biopsies at baseline (*n* = 15), at I55 (*n* = 14*) and at R10 (*n* = 14^+^); I/I with I/R (*n* = 15^#^): myocardial biopsies at baseline (*n* = 15), at I55 (*n* = 12*) and at R10 (*n* = 15); V/V with IPC + I/R (*n *= 16): myocardial biopsies at baseline (*n* = 16), after IPC (*n* = 16), at I55 (*n* = 15*; *n* = 14^+^ for AS160) and at R10 (*n* = 16); I/I with IPC + I/R (*n* = 15): myocardial biopsies at baseline (*n* = 15), after IPC (*n* = 15), at I55 (*n* = 15) and at R10 (*n* = 15); *left ventricular biopsies at I55 could not be taken due to ventricular fibrillation and intra-thoracic defibrillation; ^#^left ventricular biopsy at baseline went lost during sample preparation; ^+^missing protein lysate due to insufficient protein quantity

**Fig. 6:**
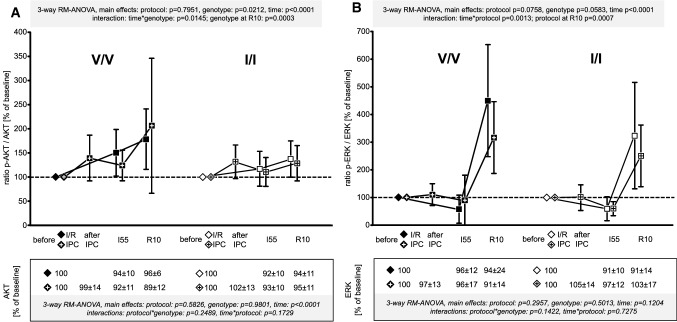
Signal ratio of phosphorylated (p) protein kinase b (AKT) (**A**) and extracellular signal-regulated kinase (ERK) (**B**) vs. total AKT and ERK in the V/V genotype (Ossabaw minipigs with homozygous valine in the γ-subunit of the AMPK; filled symbols) and the I/I genotype (Ossabaw minipigs with homozygous isoleucine in the -subunit of the AMPK; open symbols); quantification of the total protein signals is displayed in the box below the *x*-axis; + within the symbols indicates data from Ossabaw minipigs with ischaemic preconditioning (IPC) prior to ischaemia/reperfusion (I/R); all data were normalised to the baseline samples taken before the I/R or IPC protocol, respectively (indicated by diamonds); symbols are means with standard deviation; I55: 55 min ischaemia, R10: 10 min reperfusion; three-way analysis of variance (MIXED procedure; main effects protocol, genotype, and time) for repeated measures with least square means tests; V/V with I/R (*n* = 15): myocardial biopsies at baseline (*n* = 15), at I55 (*n* = 14*) and at R10 (*n* = 15); I/I with I/R (*n* = 15^#^): myocardial biopsies at baseline (*n* = 15), at I55 (*n* = 12*) and at R10 (*n* = 15); V/V with IPC + I/R (*n* = 16): myocardial biopsies at baseline (*n* = 16), after IPC (*n* = 16), at I55 (*n* = 15*) and at R10 (*n* = 16); I/I with IPC + IR (*n* = 15): myocardial biopsies at baseline (*n* = 15), after IPC (*n* = 15), at I55 (*n* = 15) and at R10 (*n* = 15); *left ventricular biopsies at I55 could not be taken due to ventricular fibrillation and intra-thoracic defibrillation; ^#^left ventricular biopsy at baseline went lost during sample preparation, consequently one Ossabaw minipig was excluded from further analysis

**Fig. 7:**
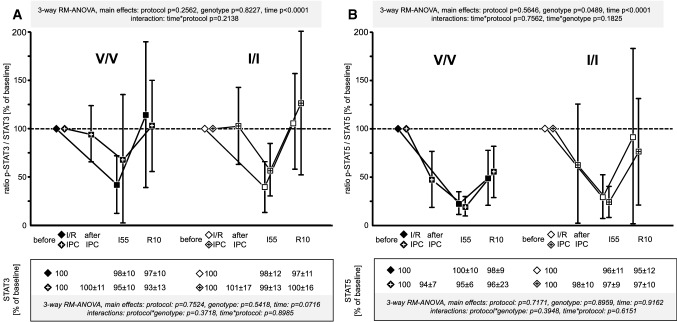
Signal ratio of phosphorylated (p) signal transducer and activator of transcription (STAT)3 (**A**) and STAT5 (**B**) vs. total STAT3 and STAT5 in the V/V genotype (Ossabaw minipigs with homozygous valine in the γ-subunit of the adenosine monophosphate-activated protein kinase; filled symbols) and the I/I genotype (Ossabaw minipigs with homozygous isoleucine in the γ-subunit of the adenosine monophosphate-activated protein kinase; open symbols); quantification of the total protein signals is displayed in the box below the *x*-axis; + within the symbols indicates data from Ossabaw minipigs with ischaemic preconditioning (IPC) prior to ischaemia/reperfusion (I/R); all data were normalised to the baseline samples taken before the I/R or IPC protocol, respectively (indicated by diamonds); symbols are means with standard deviation; I55: 55 min ischaemia, R10: 10 min reperfusion; three-way analysis of variance (MIXED procedure; main effects protocol, genotype, and time) for repeated measures with least square means tests; V/V with I/R (*n* = 15): myocardial biopsies at baseline (*n* = 15), at I55 (*n* = 14*) and at R10 (*n* = 15; *n* = 14^+^ for STAT5); I/I with I/R (*n* = 15^#^): myocardial biopsies at baseline (*n* = 15), at I55 (*n* = 12*) and at R10 (*n* = 15); V/V with IPC + I/R (*n* = 16): myocardial biopsies at baseline (*n* = 16), after IPC (*n* = 16), at I55 (*n* = 15* for STAT3; *n* = 14*^+^ for STAT5) and at R10 (*n* = 16); I/I with IPC + I/R (*n* = 15): myocardial biopsies at baseline (*n* = 15), after IPC (*n* = 15), at I55 (*n* = 15) and at R10 (*n* = 15); *left ventricular biopsies at I55 could not be taken due to ventricular fibrillation and intra-thoracic defibrillation; ^+^insufficient amount of protein lysate; ^#^left ventricular biopsy at baseline went lost during sample preparation, consequently one Ossabaw minipig was excluded from further analysis

**Fig. 8:**
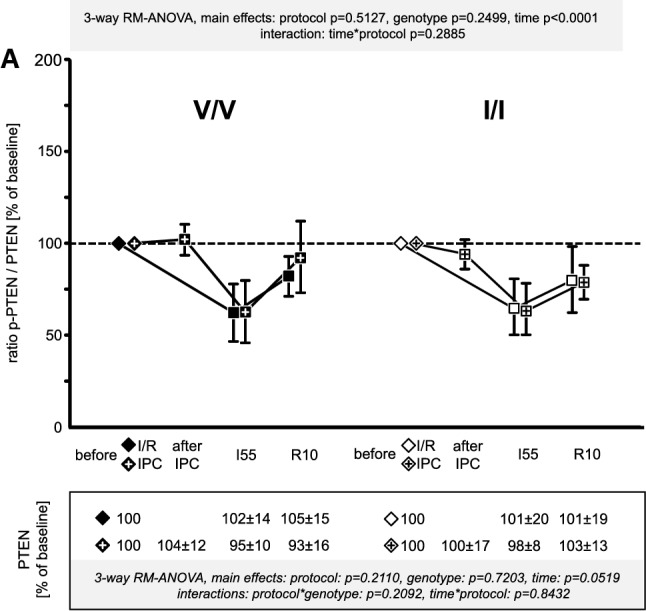
Signal ratio of phosphorylated (p) phosphatase and tensin homolog (PTEN) vs. total PTEN in the V/V genotype (Ossabaw minipigs with homozygous valine in the γ-subunit of the adenosine monophosphate-activated protein kinase; filled symbols) and the I/I genotype (Ossabaw minipigs with homozygous isoleucine in the γ-subunit of the adenosine monophosphate-activated protein kinase; open symbols); quantification of the total protein signals is displayed in the box below the *x*-axis; + within the symbols indicates data from Ossabaw minipigs with ischaemic preconditioning (IPC) prior to ischaemia/reperfusion (I/R); all data were normalised to the baseline samples taken before the I/R or IPC protocol, respectively (indicated by diamonds); symbols are means with standard deviation; I55: 55 min ischaemia, R10: 10 min reperfusion; three-way analysis of variance (MIXED procedure; main effects protocol, genotype, and time) for repeated measures with least square means tests; V/V with I/R (*n* = 15): myocardial biopsies at baseline (*n* = 15), at I55 (*n* = 14*) and at R10 (*n* = 14^+^); I/I with I/R (*n* = 15^#^): myocardial biopsies at baseline (*n* = 15), at I55 (*n* = 12*) and at R10 (*n* = 15); V/V with IPC + I/R (*n* = 16): myocardial biopsies at baseline (*n* = 16), after IPC (*n* = 16), at I55 (*n* = 14*^+^) and at R10 (*n* = 14^$^); I/I with IPC + I/R (*n* = 14^§^): myocardial biopsies at baseline (*n* = 14), after IPC (*n* = 14), at I55 (*n* = 14) and at R10 (*n* = 14); *left ventricular biopsies at I55 could not be taken due to ventricular fibrillation and intra-thoracic defibrillation; ^+^insufficient amount of protein lysate; ^$^impossible signal quantification due to fluorescence signal artefact; ^#^left ventricular biopsy at baseline went lost during sample preparation and ^§^impossible signal quantification due to fluorescence signal artefact for the baseline sample; the missing baseline values consequently led to exclusion of these two Ossabaw minipigs from further analyses

The total levels of the analysed proteins (boxes below the *x*-axis of Figs. 5, 6, 7 and 8, respectively) were stable or decreased only slightly when significantly (AMPK, AS160, AKT) over time. There were no differences between the genotypes and the protocols in the total levels of all analysed proteins. There was no significant difference between the V/V and I/I genotypes of AMPK in their phosphorylation (Fig. 5A) and activity, as identified by the phosphorylation of AS160 (Fig. 5B). Both, phosphorylation and activity decreased over time. AKT phosphorylation increased with ischaemia and reperfusion, but was not different between I/R and IPC for both genotypes (Fig. 6A). ERK phosphorylation decreased during ischaemia in both genotypes and then increased to a lesser extent at 10 min reperfusion after IPC than I/R in both genotypes (Fig. 6B). Consistent with the bioinformatic analysis as to JAK–STAT signalling, there was a decrease in STAT3 phosphorylation during ischaemia and an increase at 10 min reperfusion, which was not different between genotypes and not different between I/R without and with IPC (Fig. 7A), whereas in our contemporary study in Göttingen minipigs IPC was associated with a significant increase in STAT3 phosphorylation at 10 min reperfusion [40]. The phosphorylation of STAT5 also decreased during ischaemia in both genotypes and increased back toward baseline during early reperfusion, without a difference between I/R and IPC (Fig. 7B). The phosphorylation of PTEN decreased during ischaemia in both genotypes, irrespectively of IPC, and tended to return back toward baseline during early reperfusion (Fig. 8).

All original Western blots are displayed in supplemental Fig. 3.

## Discussion

For the present study, we have used a prospective design including a power analysis, as typically used for a randomised clinical trial. The main result of our study is: Ossabaw minipigs, in contrast to Göttingen minipigs, are not protected from infarction by IPC. This raises issues with respect to the mechanisms of cardioprotection and with respect to the clinical translation of cardioprotection.

There was no significant reduction in infarct size and area of no-reflow no matter what AMPK γ-subunit genotype the Ossabaw minipigs had. In the pre-specified secondary analysis, it did also not matter whether they were females or castrated males. IPC is the strongest cardioprotective stimulus known [20, 27], and we have used the IPC protocol which reduced infarct size robustly in the multi-centre studies of the CAESAR [35] and CIBER–CLAP consortia [69]. Apparently, the lack of cardioprotection by IPC was not related to the single nucleotide polymorphism resulting in a change from valine 199 to isoleucine in the γ-subunit of AMPK. The bioinformatic analysis identified more variants in the AMPK coding exons of Göttingen minipigs than of Ossabaw minipigs. In addition, there was apparently no difference between the AMPK γ-subunit genotypes in their protein phosphorylation/activity. Thus, it is not surprising that the AMPK γ-subunit genotype also had no impact on cardioprotection or the lack of it. AMPK has been demonstrated to be activated by IPC in mice [34, 57] and a dominant negative mutation of the α_2_-subunit abrogated enzyme activity and infarct size reduction by IPC in mice [84]. However, the AMPK γ-subunit genotype made no difference for the lack of cardioprotection, as defined by infarct size reduction, by IPC whether the Ossabaw minipigs were females or castrated males. In our contemporary study in Göttingen minipigs [40], IPC was also not associated with an increase in AMPK phosphorylation (data not shown). Apparently, AMPK is not important for cardioprotection by IPC in pigs.

A protective role for female sex against myocardial I/R injury has been proposed [62, 70], but in our contemporary study in Göttingen minipigs infarct size did not display sex-related differences [40]. In addition, infarct size was larger in the female Yorkshire pigs of the CAESAR consortium with 60 min LAD occlusion and thus even larger than in our Göttingen minipigs but still reduced significantly by IPC [35]. Thus, the lack of cardioprotection by IPC in our female and castrated male Ossabaw minipigs cannot be related to their sex being not male. Interestingly, the area of no-reflow was less in castrated males than in females - there were no apparent differences in systemic haemodynamics and regional blood flow during ischaemia and reperfusion (Table 4) -, but we could not identify pertinent data in the literature and have no explanation for this finding.

Whereas isoflurane anaesthesia may have attenuated the effects of IPC, since isoflurane as compared to, e.g., barbiturates is cardioprotective per se [36], such effect is unlikely to account for the failure of IPC to reduce infarct size in the Ossabaw pigs in our current study. In fact, infarct size without IPC was even larger than that in our contemporary Göttingen minipigs (female: 56 ± 9 vs. 45 ± 8% area at risk; castrated male: 53 ± 12 vs. 45 ± 13% area at risk), arguing against a more pronounced genuine cardioprotection in the Ossabaw than the Göttingen minipigs, and on the other hand IPC still decreased infarct size markedly in the contemporary Göttingen minipigs [40] but not in the Ossabaw minipigs. Isoflurane, in contrast to propofol, also permits cardioprotection by remote ischemic conditioning in humans [43, 93].

The neutral results of our prospectively designed study resemble the neutral results of several larger randomised controlled trials in patients undergoing interventional reperfusion of myocardial infarction [21] or cardiovascular surgery [19, 54] which used an all-comer approach and did not confirm prior smaller proof-of-concept studies on remote ischaemic conditioning [5, 18, 87]. These larger trials were neutral with respect to clinical outcome as the primary endpoint but also with respect to infarct size. In the discussion of these trials several points of critique were pointed out. The neutral cardiosurgical trials were conducted under propofol anaesthesia which is known to interfere with cardioprotection [28, 93]. In the neutral myocardial infarction trial, infarct size was determined from somewhat incomplete troponin data which do not consider for the ischaemic area at risk, and more importantly the 1-year mortality was so low in the placebo group (< 3%) that it was not possibly further reduced by any intervention [17, 30]. In fact, cardioprotection by remote ischaemic conditioning is clinically only apparent in patients with acute myocardial infarction who most need it, i.e., those with cardiac arrest or cardiogenic shock prior to interventional reperfusion [9]. In our present study, infarct size in both female and castrated male Ossabaw minipigs was somewhat larger than in female and castrated male Göttingen minipigs with the same duration of coronary occlusion and reperfusion [40]. Our primary endpoint infarct size in the power analysis-based prospective study design was not significantly changed; therefore, secondary sub-group analyses are, therefore, in a strict sense not permitted and, if anything, hypothesis generating. Nevertheless, when overstepping the do-not-test rule of the two-way ANOVA, a significant reduction of infarct size by IPC in the I/I AMPK γ-subunit genotype Ossabaw minipigs became apparent, which was, however, much less pronounced than in the Göttingen minipigs (50 ± 11 vs. 25 ± 11% area at risk in females and 51 ± 13 vs. 30 ± 8% area at risk in castrated males) [40]. When looking closer at the data, the protection in the I/I Ossabaw minipigs was mostly confined to those of female sex, i.e., a very specific subgroup had protection which was, however, hidden in the all-comer approach. Somewhat different from the above clinical studies, the I/I female Ossabaw minipigs undergoing I/R were not in greater need for cardioprotection, since infarct size in those not undergoing IPC was not different from the other subgroups. The reduction in the I/I females Ossabaw minipigs may have entirely been by chance, and it would require another prospective study to confirm it as true. Of note, however, females of both genotypes had greater coronary microvascular injury than castrated males, and we have no explanation for this difference.

When searching for a mechanism for the observed lack of cardioprotection with IPC in Ossabaw minipigs, a number of differences between Ossabaw minipigs and Göttingen minipigs became apparent in the bioinformatic DNA sequencing analysis which notably identified variant protein coding sequences and subsequently differences of mitochondrial and inflammatory (including the JAK–STAT pathway) protein–protein associations. For both mitochondria and for STAT signalling, a causal role in cardioprotection is well-established [27]. Consistent with the bioinformatic analysis, the lack of infarct size reduction in the Ossabaw minipigs was associated with the lack of increased STAT3 phosphorylation at tyrosine 705 in the Western blot analysis of established cardioprotective proteins [24] which we performed in parallel. For STAT3 activation we have previously established causality by use of blocker experiments in Göttingen minipigs experiencing cardioprotection not only by IPC [14] but also by ischaemic postconditioning [31] and remote ischaemic preconditioning [78]. Such increased STAT3 phosphorylation at 10 min reperfusion with IPC was also seen in female, castrated male and male Göttingen minipigs in our contemporary study [40]. In principle, the unchanged STAT3 phosphorylation in the present study in Ossabaw minipigs could have resulted from a lack of increased kinase activity upstream of STAT3 or a simultaneous increase of phosphatase activity downstream of STAT3. PTEN is a phosphatase which decreases STAT3 phosphorylation [8, 91], but PTEN expression and phosphorylation were not increased in the present study. Of note, the STRING data bank reference is to human proteins, and STAT3 [90] and STAT5 [33, 90] activation is associated with cardioprotection by remote ischemic conditioning in cardiosurgical patients. We realise that STAT3 is a transcription factor that is causally involved in the protection of late IPC [3, 10], but its acute activation by phosphorylation during IPC within the immediate time frame of an ischaemia–reperfusion protocol cannot induce protection by initiating transcription and increased expression of proteins as in late IPC, but must occur through other mechanisms, such as improved mitochondrial respiration [31, 89]. In the present study, ERK phosphorylation at early reperfusion which in some studies including our contemporary study in Göttingen minipigs [40] was also associated with cardioprotection, was even decreased with IPC and thus consistent with the observed lack of cardioprotection. The phosphorylation of STAT5 in the present study was also decreased during ischaemia and returned back toward baseline during early reperfusion, but was not improved by IPC. Incidentally, the established genetic predisposition of the Ossabaw minipigs to develop a metabolic syndrome found its counterpart in the appearance of clusters of protein–protein associations of mitochondria and fat metabolism, where the genomes of Ossabaw minipigs differed from that of Göttingen minipigs and *Sus scrofa*, thus serving as a positive control. We realise that we did not identify specific genes which are responsible for lack of cardioprotection by IPC but just provided a transparently structured screening of genetic differences between Ossabaw minipigs and other pig strains which might be involved in the observed lack of cardioprotection. We also realise that we just identified variants in base pair sequences in the exons of protein-encoding genes between Ossabaw minipigs on one hand and Göttingen minipigs and *Sus scrofa* on the other hand, but we do not know whether the respective proteins are expressed at all and, if so, in which cell type and which subcellular compartment exactly. The lack of cardioprotection by IPC in the Ossabaw minipigs was apparent before the true risk factors for coronary atherosclerosis and its consequences had developed under the influence of a hypercaloric and hyperlipidic diet. At the time of the study, the Ossabaw minipigs still had the non-diseased phenotype in body weight, left ventricular pressure and blood glucose and lipid levels. We did not perform functional testing with a glucose tolerance test, but it has been reported to be not impaired in lean Ossabaw minipigs of this age [45]. Thus, this lack of cardioprotection by IPC cannot be related to established co-morbidities [39]. We realise that a different IPC algorithm than the one which we used might have induced cardioprotection, but we consider this possibility were unlikely. We have used the consensus IPC protocol advocated by the CAESRAR [35] and CIBER–CLAP [69] consortia, and all published studies using IPC in pigs of various strains have reported protection, no matter which IPC algorithm was used [12, 16, 35, 40, 42, 46, 52, 58, 64, 71, 73, 75–77].

The lack of benefit from cardioprotection by IPC in the Ossabaw minipig strain is probably genetically determined as is the predisposition to develop a metabolic syndrome upon exposure to a hypercaloric and hyperlipidic diet, thus forming a double threat. Apparently, there are genuine responders and non-responders to a given cardioprotective intervention. When translating to humans, this double threat would make a strong case for **primordial** rather than primary prevention [15]. In patients with coronary artery disease, a genetically determined inability to develop a cardioprotective response to ischaemic conditioning as may occur during spontaneous episodes of myocardial ischaemia, i.e., pre-infarction angina [23, 68], would be considered a novel risk factor. The Ossabaw minipig is then a suitable model to further study such genetic lack of amenability to cardioprotection and to develop therapeutic strategies to circumvent this block in cardioprotective signal transduction. The signal transduction block could possibly be circumvented by agents and interventions that act distal to STAT3 in the cardioprotective signal transduction cascade, e.g., diazoxide [22, 24, 59] or malonate [65, 74] which target mitochondria as potential end-effectors of cardioprotection.

### Conclusions and perspective

Our neutral experimental data with a potential protection by IPC only in a small subgroup of Ossabaw minipigs resemble the clinical trials on remote ischaemic conditioning and several cardioprotective drugs in which benefits were seen in smaller, well-defined cohorts but not confirmed in larger cohorts using an all-comer approach. We have thus established an animal model of reverse translation that reproduces phenotypically the lack of cardioprotection which is seen in the recent large cardioprotection trials. We recognise that at this point the lack of infarct size reduction by IPC and the differences in protein encoding exons between Ossabaw minipigs and Göttingen minipigs are associative and not causal, but they were nevertheless consistent with the lack of increase in STAT3 phosphorylation which we have previously established as causal for cardioprotection by IPC in Göttingen minipigs. In any event, for future preclinical studies which are not reductionist on purpose with the aim to decipher a mechanism but are translational we propose that a prospective all-comer study such as the present one should be performed to avoid unrealistic expectations before a clinical trial is initiated. Such strategy for cardioprotection studies was recently advocated by the COST ACTION Cardioprotection consortium [47] and by Roberto Bolli in an editorial in this journal [2].

## Supplementary Information

Below is the link to the electronic supplementary material.

Supplementary file1 (PDF 8958 KB)

Supplementary file2 (XLSX 1158 KB)

Supplementary file3 (XLSX 42 KB)
