# Supplementary material for: Non-responsiveness to cardioprotection by ischaemic preconditioning in Ossabaw minipigs with genetic predisposition to, but without the phenotype of the metabolic syndrome
Source: Basic Res Cardiol. 2022 Nov 11;117(1):58. doi: 10.1007/s00395-022-00965-0 (PMC9652280; doi:10.1007/s00395-022-00965-0)
Supplement: Supplementary file 1 — Supplementary file1 (PDF 8958 KB) [file 395_2022_965_MOESM1_ESM.pdf]

Supplemental Tables and Figures

Non-responsiveness to cardioprotection by ischaemic preconditioning in Ossabaw minipigs with genetic predisposition to, but without the phenotype of the metabolic syndrome

Petra Kleinbongard[1], PhD; Helmut Raphael Lieder[1], MD; Andreas Skyschally[1], PhD; Mouhamad Alloosh[2], MD; Axel Gödecke[3], PhD; Sven Rahmann[4], PhD; Michael Sturek[2], PhD; Gerd Heusch[1], MD

[1]Institute for Pathophysiology, West German Heart and Vascular Center, University of Essen Medical School, University of Duisburg-Essen, Essen, Germany; [2]Department of Anatomy, Cell Biology, & Physiology, Indiana University School of Medicine, Indianapolis, USA; [3]Institute for Cardiovascular Physiology, University Hospital and Heinrich-Heine University, Düsseldorf, Germany; [4]Algorithmic Bioinformatics, Center for Bioinformatics and Department of Computer Science, Saarland University, Saarbrücken, Germany

Short title: Lack of cardioprotection in Ossabaw minipigs

Supplemental Table 1

accession number	pig strain	pig name / ID(#)	sex	genotype	reads	Gbp	Cov.	*T*
SAMN28202074	Ossabaw minipig	Todd, #P2110	m	I/I	750275394	225.083	87.2x	15
SAMN28202075	Ossabaw minipig	Vladir, #P2536	m	V/V	711942413	213.583	82.8x	14
SAMN28202076	Ossabaw minipig	Valencia, #P2540	f	V/V	1055076183	316.523	122.7x	23
SAMN28202077	Ossabaw minipig	Anarietta, #P2638	f	I/I	672374388	201.712	78.2x	14
SAMN28202078	Ossabaw minipig	Kangaroo, #P2781	f	I/I	649794528	194.938	75.6x	14
SAMN28202079	Ossabaw minipig	Valor, #P3057	m	V/V	766072312	229.822	89.1x	16
SAMN28202080	Ossabaw minipig	Novak, #P3080	m	I/I	642446760	192.734	74.7x	13
SAMN28202081	Ossabaw minipig	Valentine, #P3247A	f	V/V	972190118	291.657	113.0x	22
ERR2744277	Göttingen minipig	-	f	-	183937614	36.788	14.26x	3
ERR2744278	Göttingen minipig	-	f	-	195476661	39.095	15.15x	3
ERR2744279	Göttingen minipig	-	f	-	199177973	39.836	15.44x	4
ERR2744280	Göttingen minipig	-	f	-	195578756	39.116	15.16x	4
ERR2744281	Göttingen minipig	-	f	-	193841291	38.768	15.03x	4
ERR2744282	Göttingen minipig	-	f	-	191300443	38.260	14.83x	3
ERR2744283	Göttingen minipig	-	f	-	212701827	42.540	16.49x	4
ERR2744284	Göttingen minipig	-	f	-	205724225	41.145	15.95x	3
ERR2744285	Göttingen minipig	-	f	-	207048949	41.410	16.05x	4
ERR2744286	Göttingen minipig	-	f	-	184378852	36.876	14.29x	3

f: female pigs; m: male pigs; V/V: pigs with homozygous valine in the γ-subunit of the adenosine monophosphate-activated protein kinase; I/I: pigs with homozygous isoleucine in the γ-subunit of the adenosine monophosphate-activated protein kinase

Details to genome sequences of Ossabaw minipigs and Göttingen minipigs. For each individual pig, the accession number, name and ID number (if assigned), sex, genotype, the number of sequenced reads (length 2x150 for Ossabaw minipigs, 2x100 for Göttingen minipigs), the total number of sequenced giga-basepairs (Gbp), the average genome coverage (Cov.) with an assumed genome size of 2.58 Gbp, and k-mer frequency threshold values (T) are given.

Ossabaw minipig genome data are made available at the National Library of Medicine, Sequence Read Archive, Bioproject PRJNA837405. Göttingen minipig genome data were retrieved from the European Nucleotide Archive; project number PRJEB27654.

Supplemental Table 2

a)	0	1	2	3	4	5	6	7	8	9	10
0	0	57035093	38862945	35151135	31553520	27805367	26773156	25759814	25295282	26150470	47372397
1	24320458	2098980	1847970	1645666	1656652	1634977	1662051	1782188	1877735	2113077	4185046
2	19896290	2401276	2274538	2029623	1966666	1902474	2017209	2213450	2598534	2809455	6142604
3	22187746	3390402	2560092	2697499	2624967	2618272	2514164	2741767	2955843	3588000	7899336
4	40030623	5920134	4596084	4698928	4726133	4517352	4412569	4637822	5341669	6483151	14515516
5	15495903	2804766	2389608	2496686	2308956	2362817	2493170	2668560	2977852	3770057	9348315
6	16938364	3414571	2798878	2640339	2814446	2833028	3070524	3411276	3703636	4134549	10414518
7	21525984	5090591	4449718	4582449	4508965	4649142	4887736	5479092	6124176	8269817	23764923
8	81743088	32774421	31892905	34336990	37313575	41191431	46995021	58605678	81046297	166366800	1382889342

b)	0	1	2	3	4	5	6	7	8	9	10
0	0	58450462	40003471	36373574	32758807	28901931	27974797	27150061	26817936	28010761	51120295
1	41468298	5424360	4542564	4443309	4482620	4488570	4200238	4393166	4790767	5484413	12091093
2	50681098	8454788	6850340	6752200	6697011	6475727	6654084	7296053	8248667	9827656	22479232
3	33867109	7600528	6290026	6377178	6236971	6425026	6829664	7494218	8239265	10289315	26444078
4	93576519	35000096	33986337	36333054	39298471	43223606	49166817	60966149	83824389	170073231	1394397299

a) Number of k-mers that belong to i out of 8 Ossabaw minipig individuals (row number i) and j out of 10 Göttingen minipig individuals (column number j) for all pairs $0 \leq i \leq 8$ and $0 \leq j \leq 10$.

b) Number of k-mers that belong to i out of 4 female Ossabaw minipig individuals (row number i) and j out of 10 female Göttingen minipig individuals (column number j) for all pairs $0 \leq i \leq 4$ and $0 \leq j \leq 10$.

Supplemental Table 3

***Sus scrofa* reference genome data**	Sscrofa11.1; RefSeq: GCF_000003025.6*		
**Annotation file**	GFF file, gene interval annotations https://ftp.ncbi.nlm.nih.gov/genomes/all/GCF/000/003/025/GCF_000003025.6_Sscrofa11.1/GCF_000003025.6_Sscrofa11.1_genomic.gff.gz	protein table, download at https://www.ncbi.nlm.nih.gov/genome/browse/#!/proteins/84/317145%7CSus%20scrofa/	GFF file, exon annotations for each gene https://ftp.ncbi.nlm.nih.gov/genomes/all/GCF/000/003/025/GCF_000003025.6_Sscrofa11.1/GCF_000003025.6_Sscrofa11.1_genomic.gff.gz
**sequence interval type**	total gene interval	from first to last coding base (including introns)	exon intervals
**total number of annotated sequences**	25875	19558	25424
**LOCxxxx sequences**	8711	3229	8705
**MIRxxxx sequences**	307	0	307
**TRNAxxxx sequences**	459	11	58
**sequences with assigned protein name**	16398	16318	16354

* representative reference genome assembly from NCBI for *Sus scrofa*, submitted by “The Swine Genome Sequencing Consortium (SGSC)”; LOCxxxx: annotated protein-coding sequences without assigned protein name; MIRxxxx: sequences annotated as micro RNAs; TRNAxxxx: sequences annotated as transfer RNAs; LOCxxxx, MIRxxxx, and TRNAxxxx sequences were excluded in the numbers reported from Ossabaw minipigs and Göttingen minipigs.

**Göttingen minipig genome data**	**European Nucleotide Archive (project number PRJEB27654, accession numbers ERR2744277 - ERR2744286) data from 10 ♀ Göttingen minipigs**					
**Ossabaw minipig genome data**	**NCBI Sequence Read Archive (project number PRJNA837405, accession numbers SAMN28202074 – SAMN28202081)**					
	data from 4 ♀ Ossabaw minipigs			data from 4 ♀ and 4 ♂ Ossabaw minipigs		
**sequence frame type**	total gene	from first to last coding base	exons	total gene	from first to last coding base	exons
**sequences with altered bp in Ossabaw minipigs**	11522	10517	6858	8040	7220	3873
**sequences with variants in Ossabaw minipigs**	13217	12139	7821	10485	9505	4673
**sequences with altered bp in Ossabaw minipigs and zero altered bp in Göttingen minipigs**	726	1154	906	380	604	480
**sequences with variants in Ossabaw minipigs and zero variants in Göttingen minipigs**	261	474	563	126	227	294

bp: base pairs

More than 10.000 altered bp sequences were identified in the 4 female Ossabaw minipigs, slightly over 8.000 also in the larger cohort of 4 female and 4 castrated male Ossabaw minipigs. To contrast differences between the Ossabaw minipigs on the one hand and Göttingen minipigs and *Sus scrofa* on the other hand, we focused on sequences with variants in the exons of protein-encoding genes.

Supplemental Table 4

antibody against	primary antibody	Cell Signaling catalogue number	dilution	incubation	secondary antibody	Li-COR (1) Cell Signaling (2) catalogue number	dilution	incubation
AKT$_{ser473}$*	rabbit polyclonal	9271	1:150	overnight at 4°C	goat anti-rabbit IgG	(1) 926-32211	1:4000	2 h at RT
AKT*	mouse monoclonal	58295	1:1000	2 h at RT	goat anti-mouse IgG	(1) 926-68070	1:10000	1 h at RT
AMPK$_{thr172}^{\#}$	rabbit monoclonal	2535	1:500	overnight at 4°C	goat anti-rabbit IgG	(1) 926-32211	1:10000	1h at RT
AMPK$^{\#}$	mouse monoclonal	2793	1:500	1 h at RT	goat anti-mouse IgG	(1) 926-68070	1:10000	1h at RT
AS160$_{thr642}$*	rabbit monoclonal	8881	1:500	overnight at 4°C	goat anti-rabbit IgG	(2) 7074	1:4000	1h at RT
AS160*	rabbit monoclonal	2670	1:500	overnight at 4°C	goat anti-rabbit IgG	(2) 7074	1:5000	1h at RT
ERK1/2$_{thr202-tyr204/thr185-tyr187}^{\#}$	rabbit polyclonal	9101	1:500	overnight at 4°C	goat anti-rabbit IgG	(1) 926-32211	1:20000	2 h at RT
ERK$^{\#}$	mouse monoclonal	4696	1:500	1 h at RT	goat anti-mouse IgG	(1) 926-68070	1:10000	2 h at RT
PTEN$_{ser380/thr382/383}$	rabbit monoclonal	9549	1:500	overnight at 4°C	goat anti-rabbit IgG	(1) 926-32211	1:5000	1h at RT
PTEN	mouse monoclonal	14642	1:500	overnight at 4°C	goat anti-mouse IgG	(1) 926-68070	1:5000	1h at RT
STAT3$_{tyr705}$*	mouse monoclonal	9138	1:250	overnight at 4°C	goat anti-mouse IgG1	(1) 926-32350	1:5000	1 h at RT
STAT3*	mouse monoclonal	9139	1:2000	overnight at 4°C	goat anti-mouse IgG2a	(1) 926-68051	1:10000	2 h at RT
STAT5$_{tyr694}$*	rabbit monoclonal	4322	1:500	overnight at 4°C	goat anti-rabbit IgG	(2) 7074	1:4000	1h at RT
STAT5*	rabbit monoclonal	94205	1:500	overnight at 4°C	goat anti-rabbit IgG	(2) 7074	1:4000	1h at RT

*sequential incubation of primary and secondary antibodies; # sequential incubation of primary antibodies for phosphorylated and total form of the protein, respectively, and simultaneous incubation of the respective secondary antibodies; AKT: protein kinase B; AMPK: AMP-activated protein kinase; AS160: 160 kDa substrate of the AKT Ser/Thr kinase; ERK: mitogen extracellular-regulated-kinase; PTEN: phosphatase and tensin homolog; STAT: signal transducer and activator of transcription; RT: room temperature

Protein phosphorylation sites, ordering numbers of primary and secondary antibodies, antibodies working concentration and antibody incubation times.

Supplemental Table 5

			Ossabaw minipig		Göttingen minipig	
protein	**annotated sequence**	**sequence length**	**altered bp / Mbp**	**variants / Mbp**	**altered bp / Mbp**	**variants / Mbp**
AKT2	AKT2	8423	0	119	163362	3324
AKT3	AKT3	7945	4405	2140	45563	755
AMPK	PRKAG3	2022	0	0	106825	7418
ERK1	MAPK3	1844	542	542	419197	1085
ERK2	MAPK1	4184	239	239	163002	239
STAT3	STAT3	2473	0	0	46098	1213
STAT5A	STAT5A	3835	0	0	56584	4954
STAT5B	STAT5B	5086	0	0	47975	1966

AKT: protein kinase B; AMPK: AMP-activated protein kinase; ERK: mitogen extracellular-regulated-kinase; STAT3: signal transducer and activator of transcription 3; STAT5: signal transducer and activator of transcription 5

Excerpt of supplemental data file “Altered base pairs and variants in Ossabaw minipigs and Göttingen minipigs.xlsx”; altered base pairs (bp) and variants, both normalised to 1 Mbp, in Ossabaw minipigs and Göttingen minipigs for the proteins AKT2, AKT3, AMPK, ERK1, ERK2, STAT3, STS5A, AND STAT5B which have been subjected to Western blot analysis for changes in phosphorylation during ischaemia/reperfusion with and without cardioprotection.

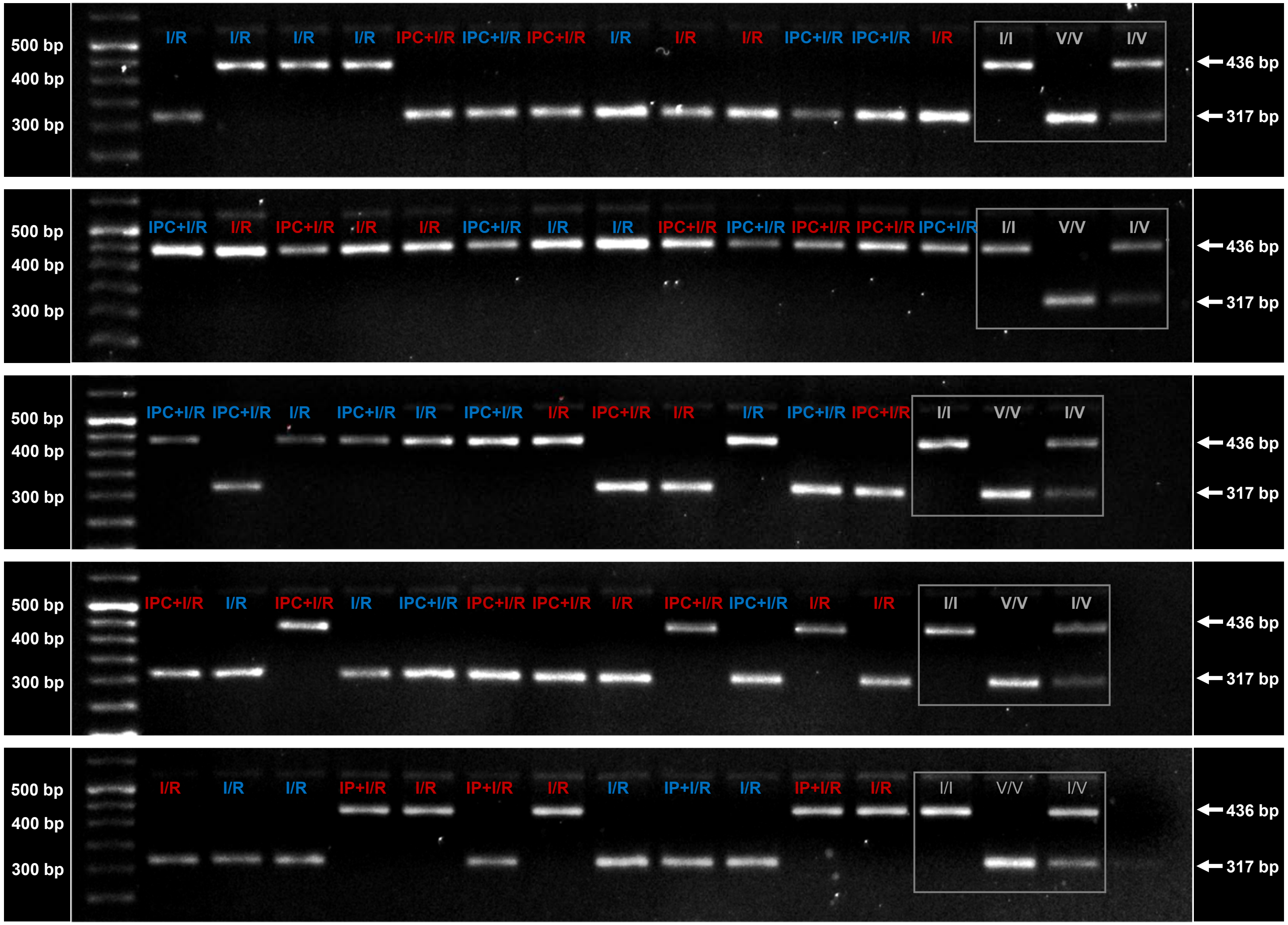

Supplemental Figure 1. Genotyping for the individual Ossabaw minipigs. Identification of homozygous point mutation resulting in a change from valine 199 to isoleucine in the γ-subunit of adenosine monophosphate-activated protein kinase (I/I) with the 436 bp product and of homozygous (V/V) valine 199 in the γ-subunit of adenosine monophosphate-activated protein kinase by the 317 bp product. Heterozygous Ossabaw minipigs (I/V) can be identified via both products 436 and 317 bp, respectively. On each gel, DNA of the 3 possible genotypes was loaded as reference (box on the right side, respectively).
I/R=ischaemia/reperfusion; IPC+I/R=ischaemic preconditioning+ischaemia/reperfusion.

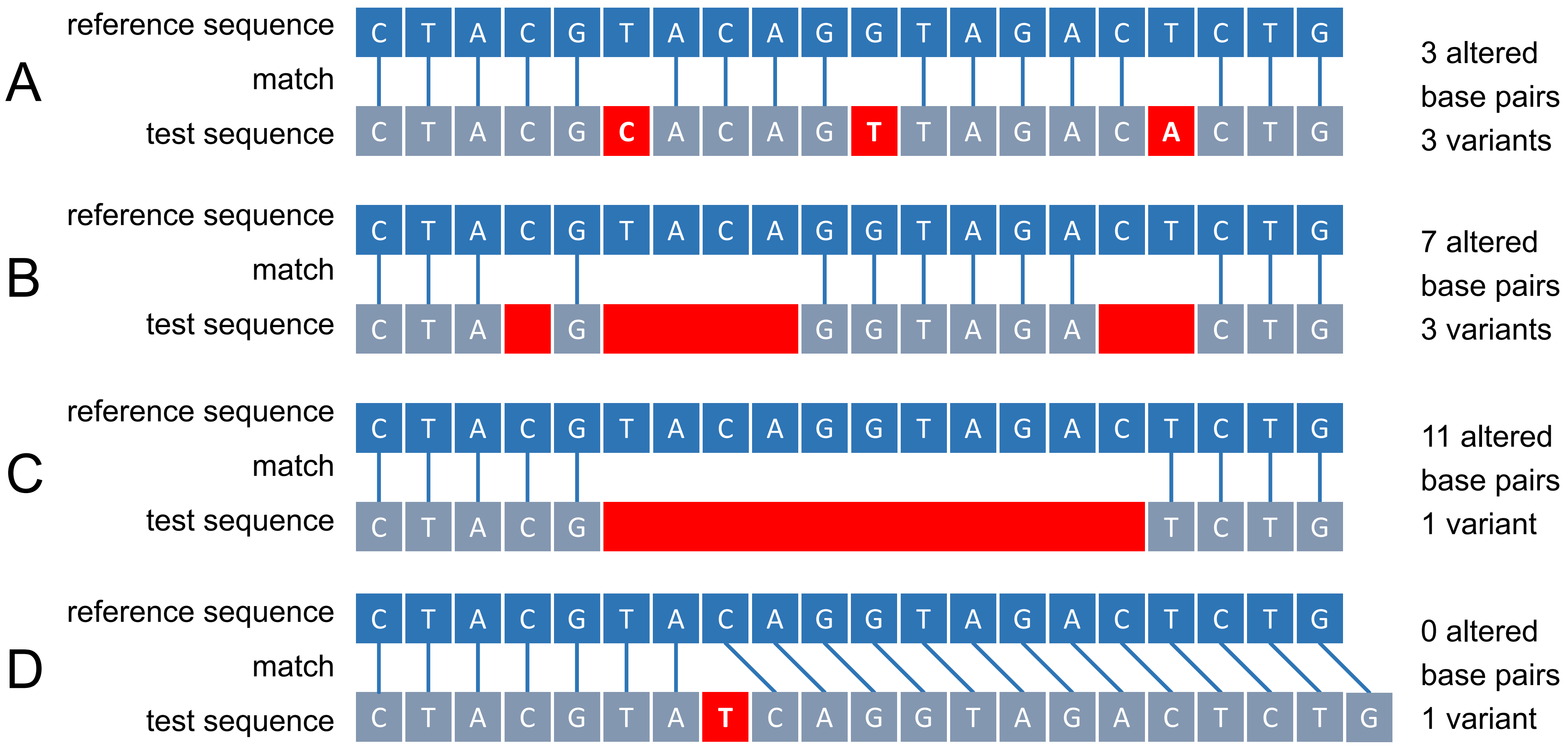

Supplemental Figure 2. Altered base pairs and variants: Altered base pairs can correspond to deleted or changed base pairs (A, B). A block of consecutive altered base pairs is called a variant (C). Another type of variant, not discovered by altered base pairs, but by reference k-mers which do not belong to the genomic content of one of the other strains, are insertions (D).

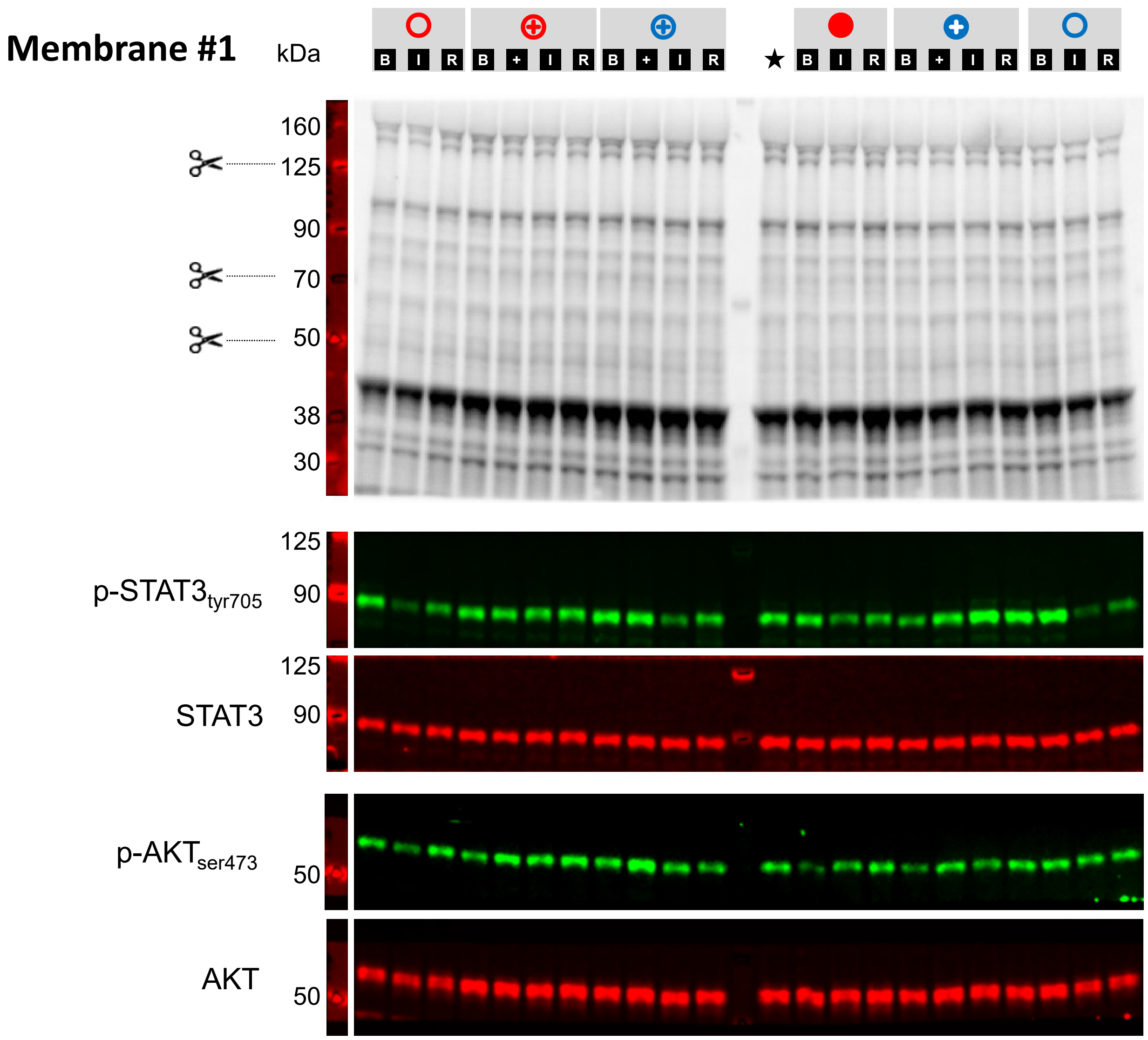

Supplemental Figure 3. Full unedited membranes for validation of equal protein loading; total protein is visualised by an ultraviolet light-induced fluorescence reaction of protein-tryptophan with tri-halocompounds. Dotted lines indicate where the membrane was cut horizontally. Marker proteins are visualised via red fluorescence/luminescence signals, respectively. The membrane sections are the areas chosen for signal intensity analysis.

Samples from females are indicated by red, from castrated males by blue symbols; V/V genotype (Ossabaw minipigs with homozygous valine in the γ-subunit of the adenosine monophosphate-activated protein kinase, AMPK) are indicated by filled symbols, and I/I genotype (Ossabaw minipigs with homozygous isoleucine in the γ-subunit of the AMPK) by open symbols.

(legend continued on next page...)

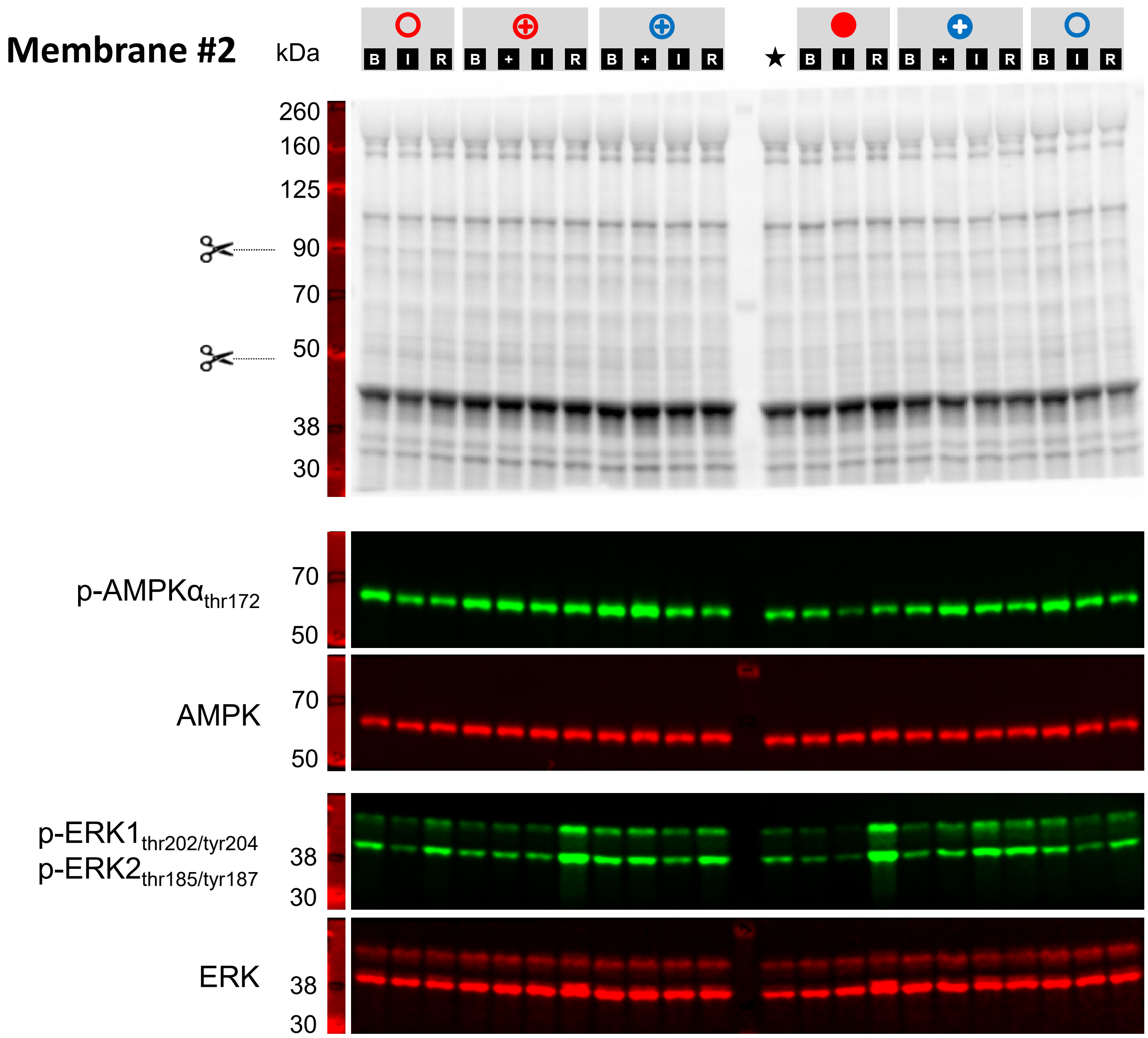

Individual samples are marked with B=baseline; +=after ischaemic preconditioning (IPC); I=55 min ischaemia; R=10 min reperfusion, respectively. ★ indicates the reference sample, which was loaded on each gel for subsequent data normalisation. *indicates the left ventricular biopsy at I (55 min ischaemia) could not be taken due to ventricular fibrillation and intra-thoracic defibrillation. + on membranes #3 and #21 indicates a missing protein lysate; due to a very small left ventricular biopsy, resulting in low protein quantity. A white **X** above lanes in membranes #18 and #30 indicate fluorescence signal artefacts resulting in impossible signal quantification.

AKT: protein kinase b; AS160: 160 kDa substrate of the AKT Ser/Thr kinase; ERK: extracellular signal-regulated kinase; PTEN: phosphatase and tensin homolog; STAT: signal transducer and activator of transcription.

(legend continued on next page...)

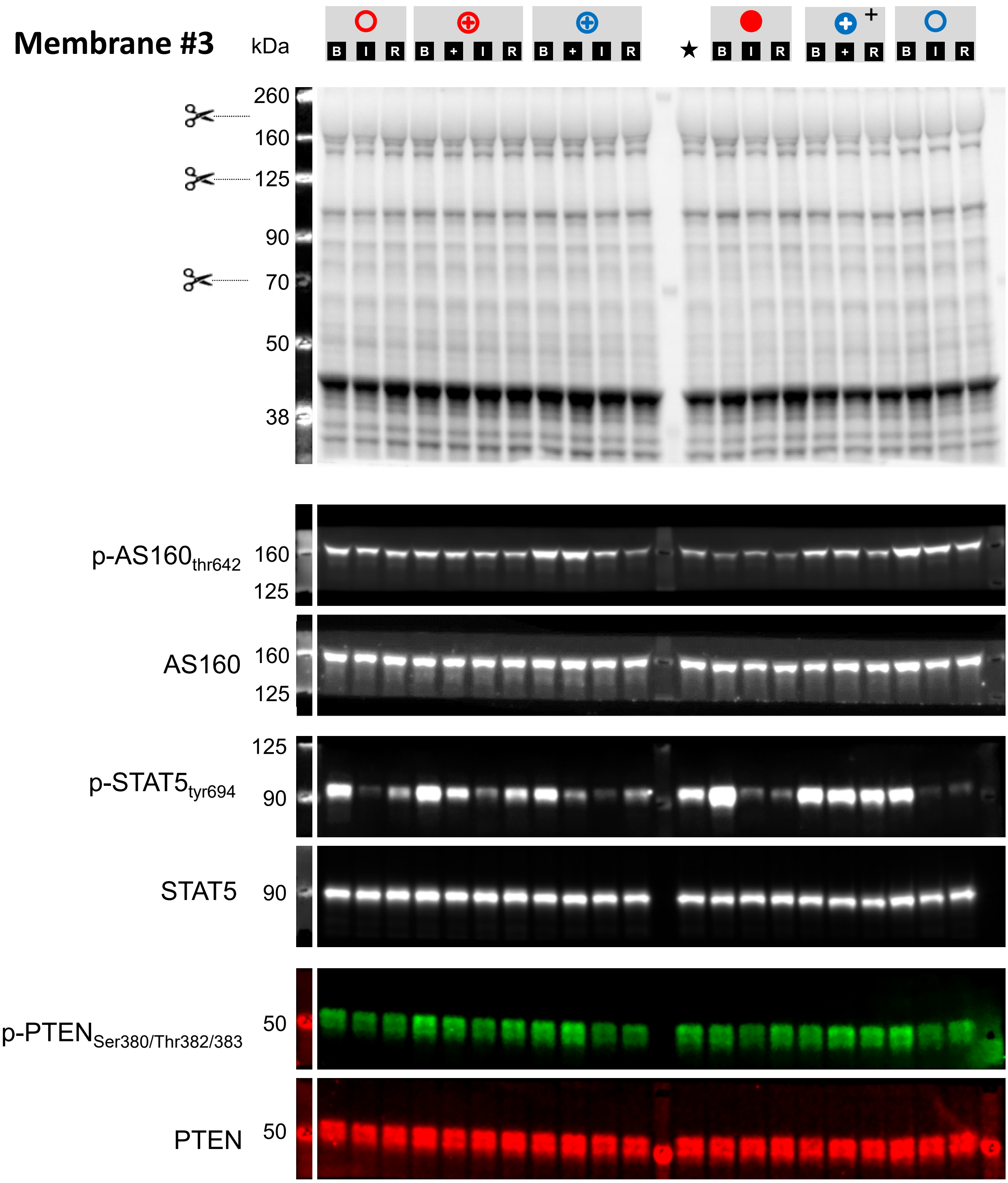

V/V females with ischaemia/reperfusion (I/R; n=7): myocardial biopsies at baseline (n=7), at I55 (n=6) and at R10 (n=7; n=6 for AS160, STAT5, and PTEN); V/V castrated males with I/R (n=8): myocardial biopsies at baseline (n=8), at I55 (n=8) and R10 (n=8); I/I females with I/R (n=8): myocardial biopsies at baseline (n=8), at I55 (n=7) and at R10 (n=8); I/I castrated males with I/R (n=7): myocardial biopsies at baseline (n=7), at I55 (n=5) and R10 (n=7); V/V females with IPC+I/R (n=8): myocardial biopsies at baseline (n=8), after IPC (n=8), at I55 (n=8) and at R10 (n=8);

(legend continued on next page...)

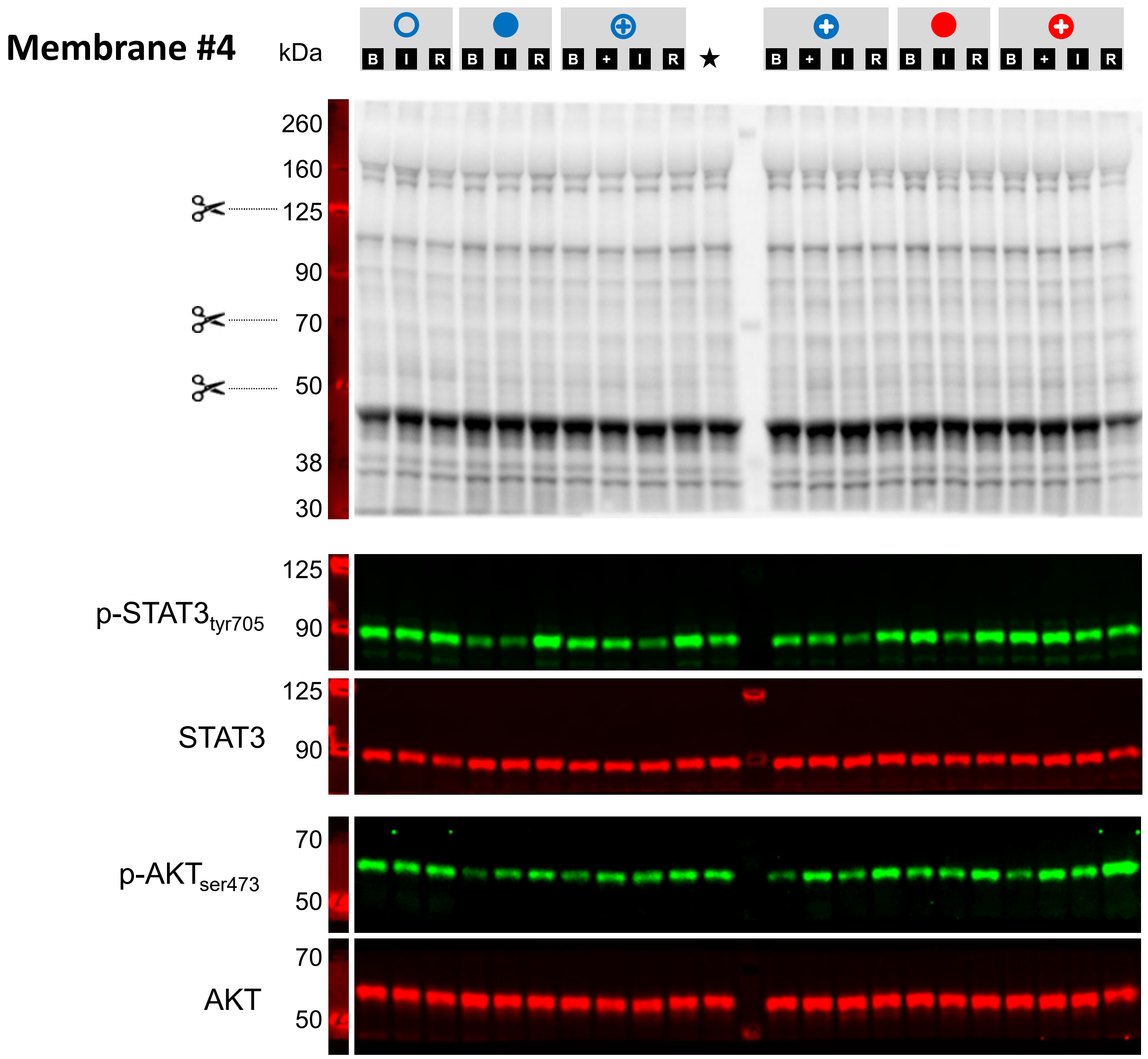

V/V castrated males with IPC+I/R (n=8): myocardial biopsies at baseline (n=8), after IPC (n=8), at I55 (n=7; n=6 for AS160, STAT5, and PTEN) and R10 (n=8); I/I females with IPC+I/R (n=7): myocardial biopsies at baseline (n=7), after IPC (n=7), at I55 (n=7) and at R10 (n=7); I/I castrated males with IPC+I/R (n=8): myocardial biopsies at baseline (n=8), after IPC (n=8), at I55 (n=8) and R10 (n=8).

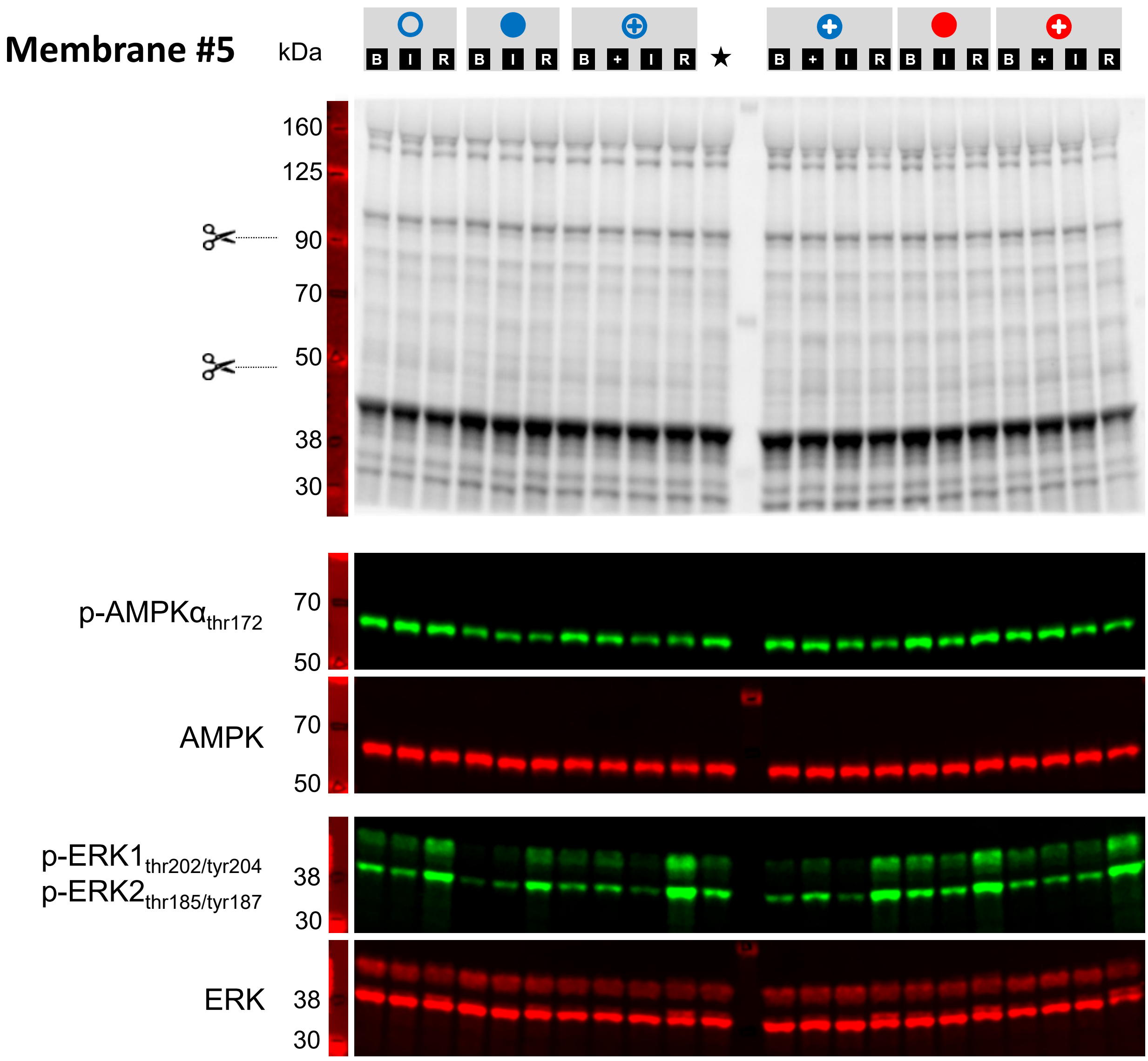
Membrane #5
kDa
B I R B I R B + I R ★ B + I R B I R B + I R
160
125
90
70
50
38
30
p-AMPKα thr172
70
50
AMPK
70
50
p-ERK1 thr202/tyr204
p-ERK2 thr185/tyr187
38
30
ERK
38
30

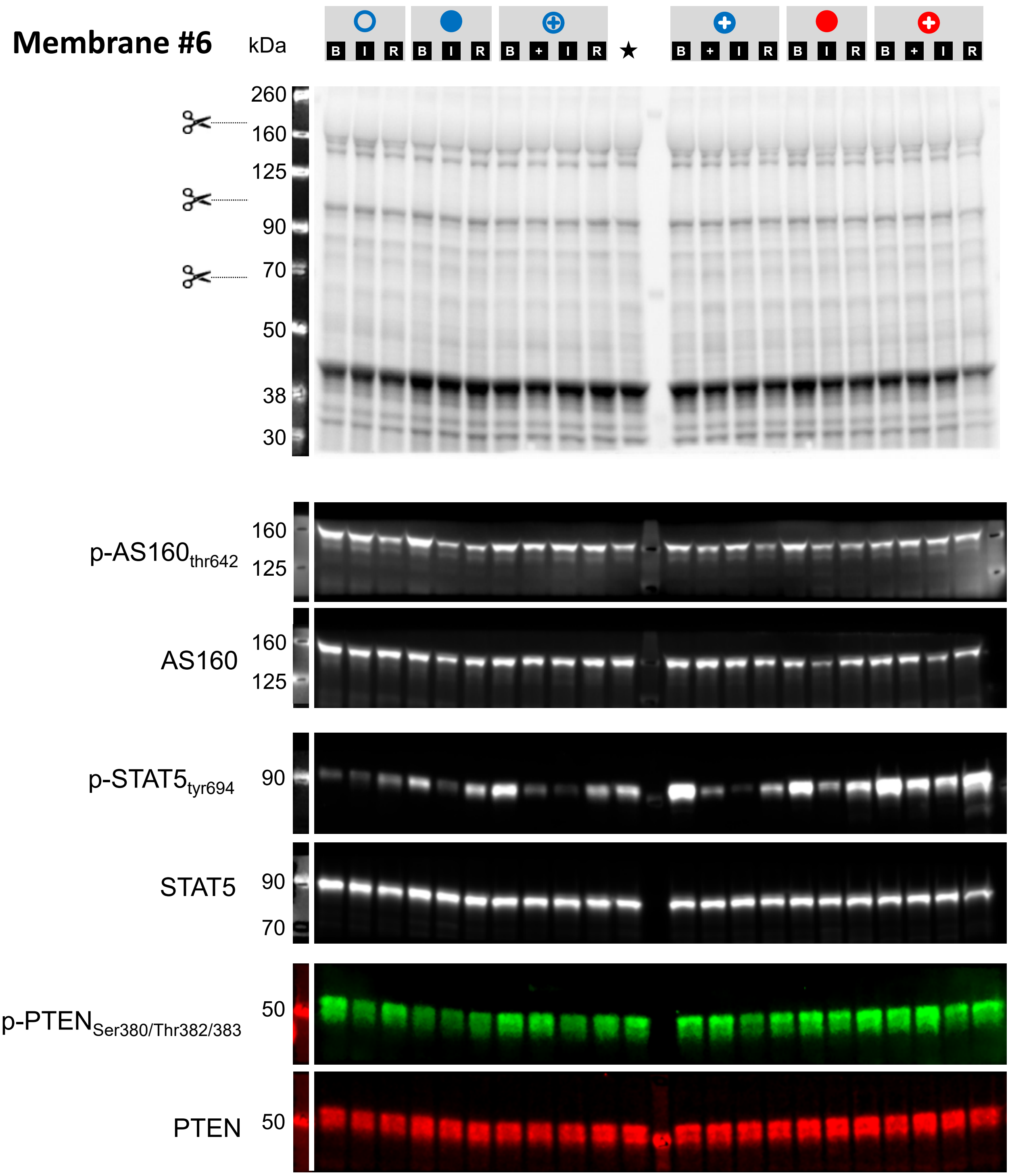

Membrane #6
kDa
B I R B I R B + I R ★ B + I R B I R B + I R
260
160
125
90
70
50
38
30
p-AS160thr642
160
125
AS160
160
125
p-STAT5tyr694
90
STAT5
90
70
p-PTENSer380/Thr382/383
50
PTEN
50

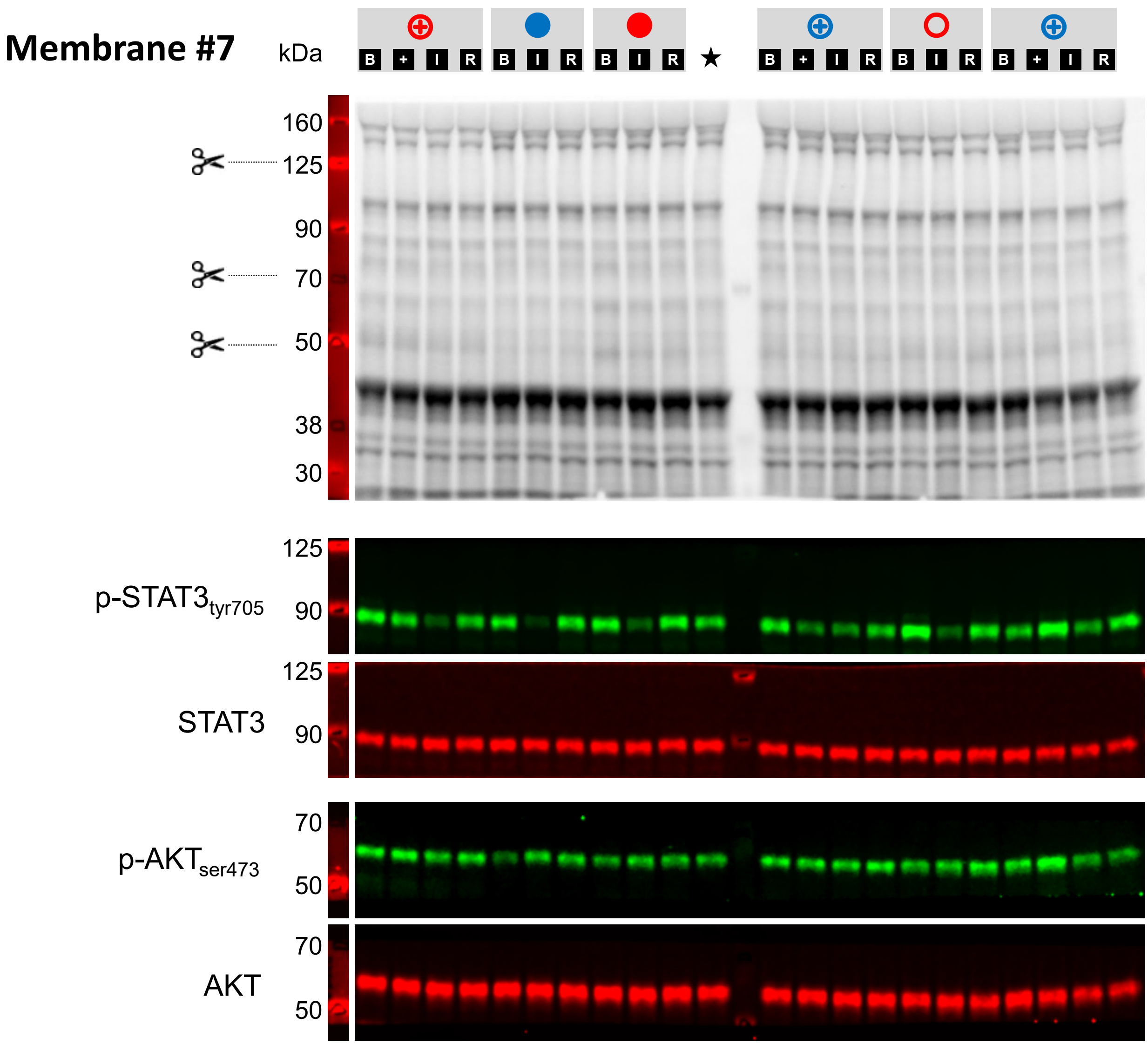
Membrane #7
kDa
B + I R B I R B I R ★ B + I R B I R B + I R
160
125
90
70
50
38
30
p-STAT3tyr705
125
90
STAT3
125
90
p-AKTser473
70
50
AKT
70
50

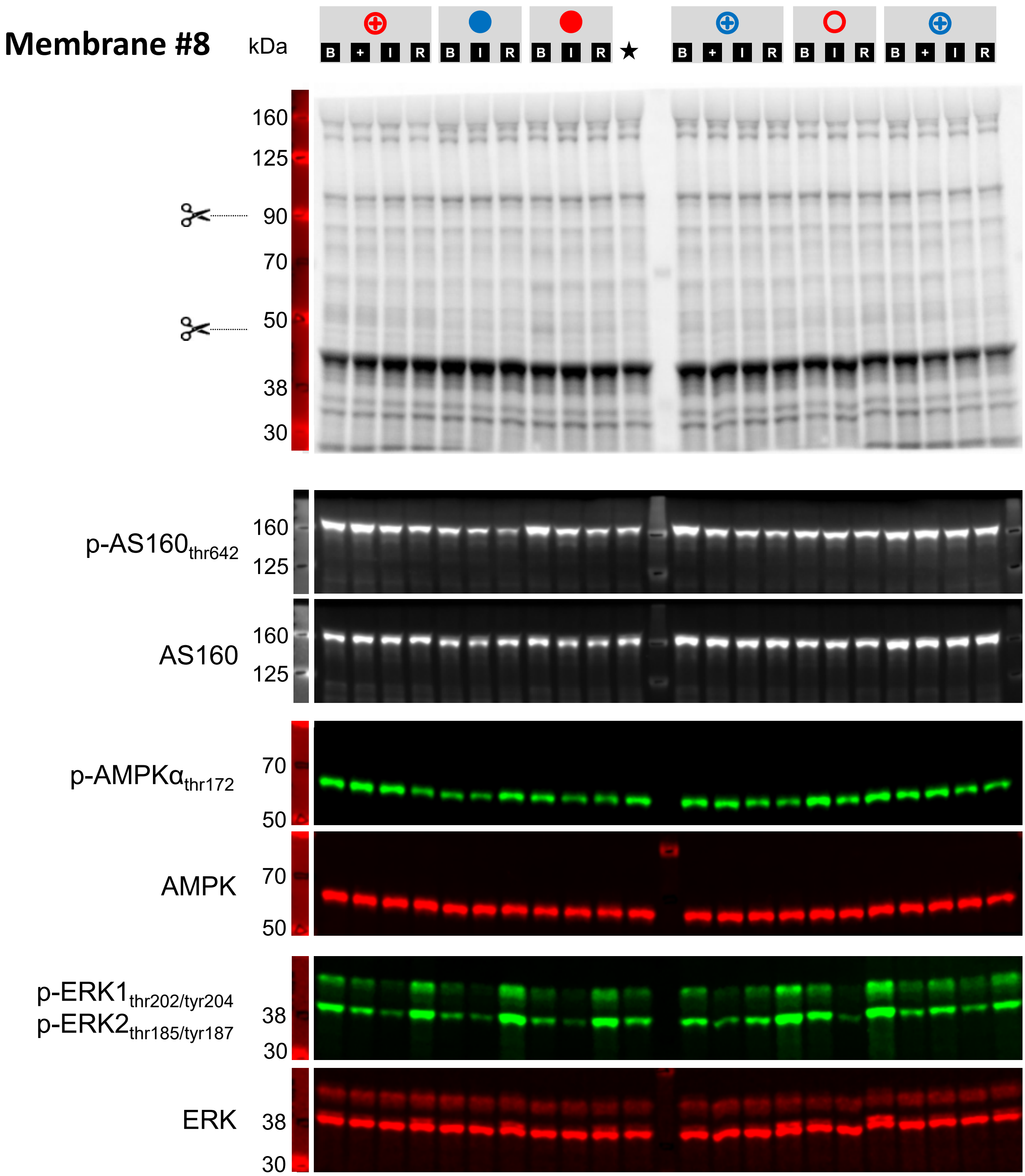
Membrane #8
kDa
B + I R B I R B I R ★ B + I R B I R B + I R
160
125
90
70
50
38
30
p-AS160thr642
160
125
AS160
160
125
p-AMPKαthr172
70
50
AMPK
70
50
p-ERK1thr202/tyr204
p-ERK2thr185/tyr187
38
30
ERK
38
30

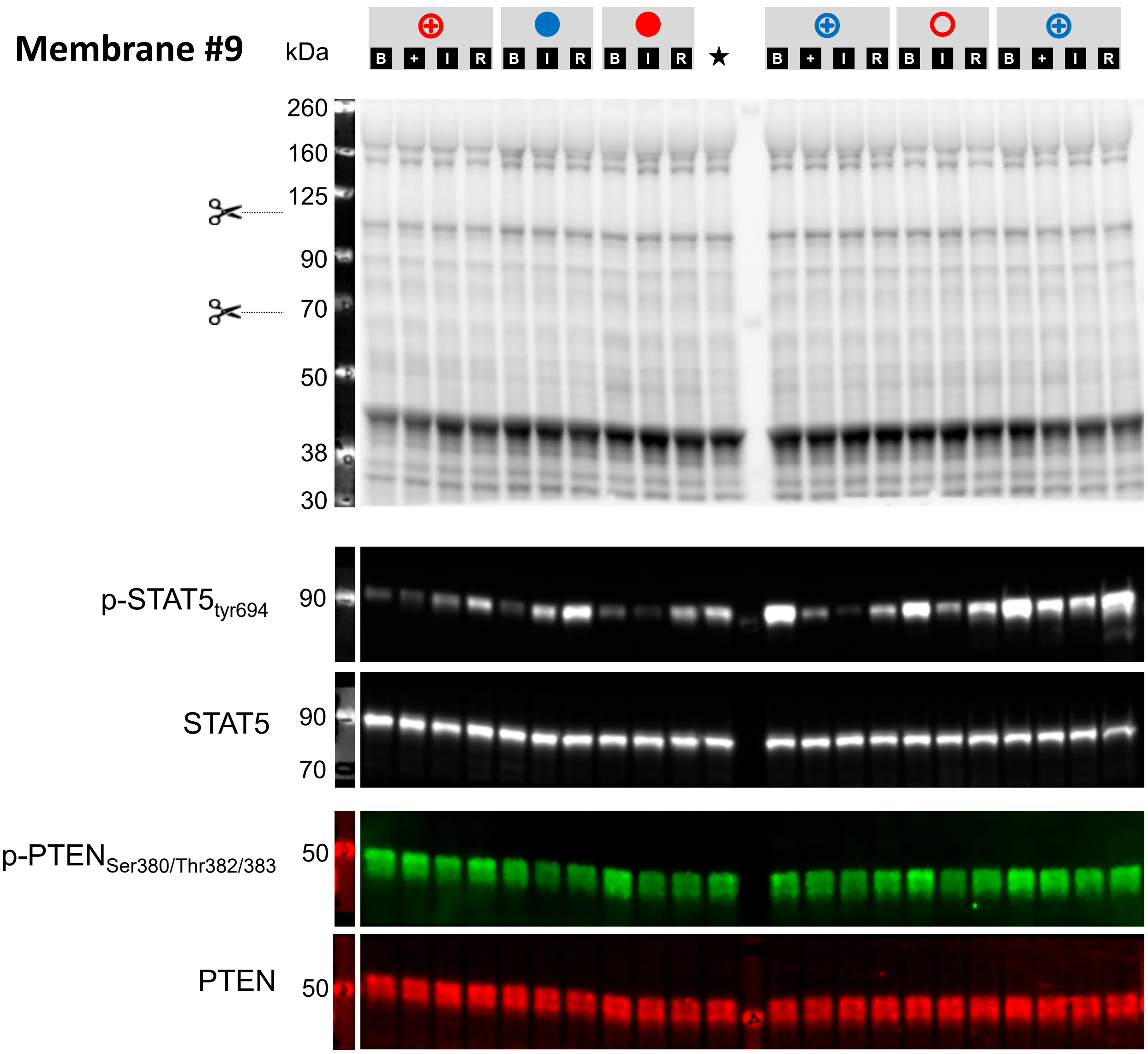
Membrane #9
kDa
B + I R B I R B I R ★ B + I R B I R B + I R
260
160
125
90
70
50
38
30
p-STAT5tyr694
90
STAT5
90
70
p-PTENSer380/Thr382/383
50
PTEN
50

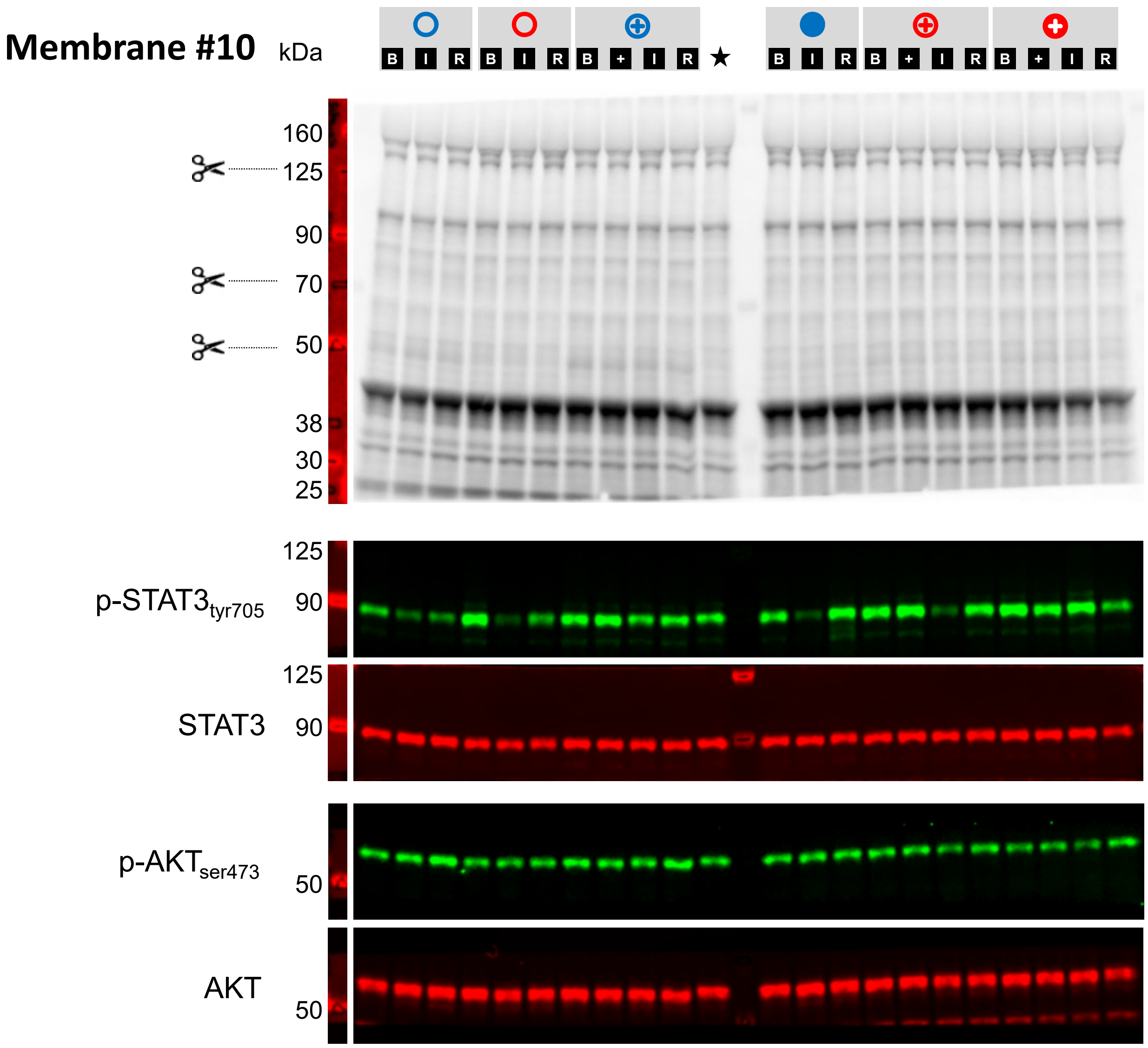
Membrane #10
kDa
B I R B I R B + I R ★ B I R B + I R B + I R
160
125
90
70
50
38
30
25
125
p-STAT3tyr705
90
125
STAT3
90
p-AKTser473
50
AKT
50

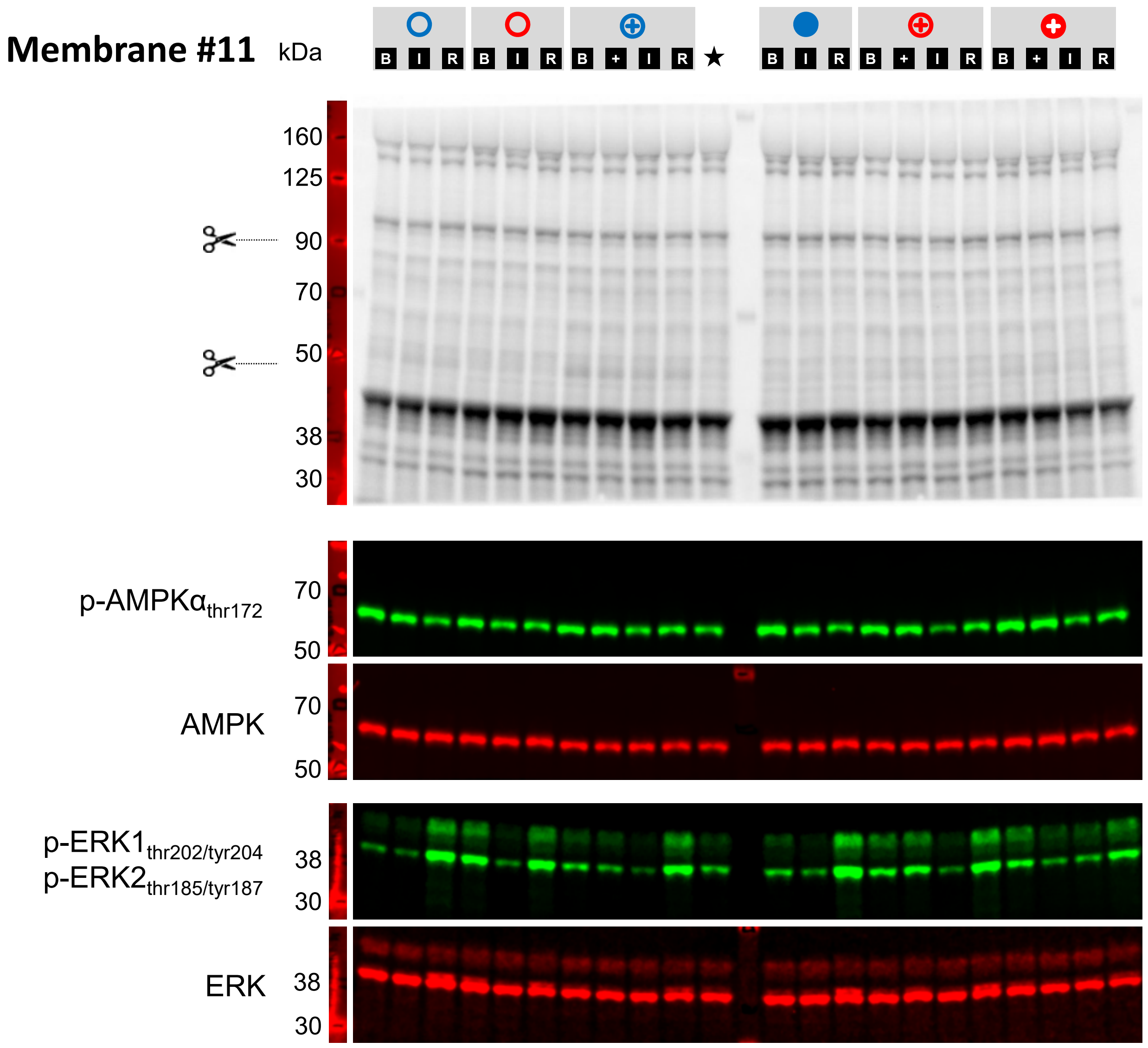
Membrane #11
kDa
B I R B I R B + I R ★ B I R B + I R B + I R
160
125
90
70
50
38
30
p-AMPKα thr172
70
50
AMPK
70
50
p-ERK1 thr202/tyr204
p-ERK2 thr185/tyr187
38
30
ERK
38
30

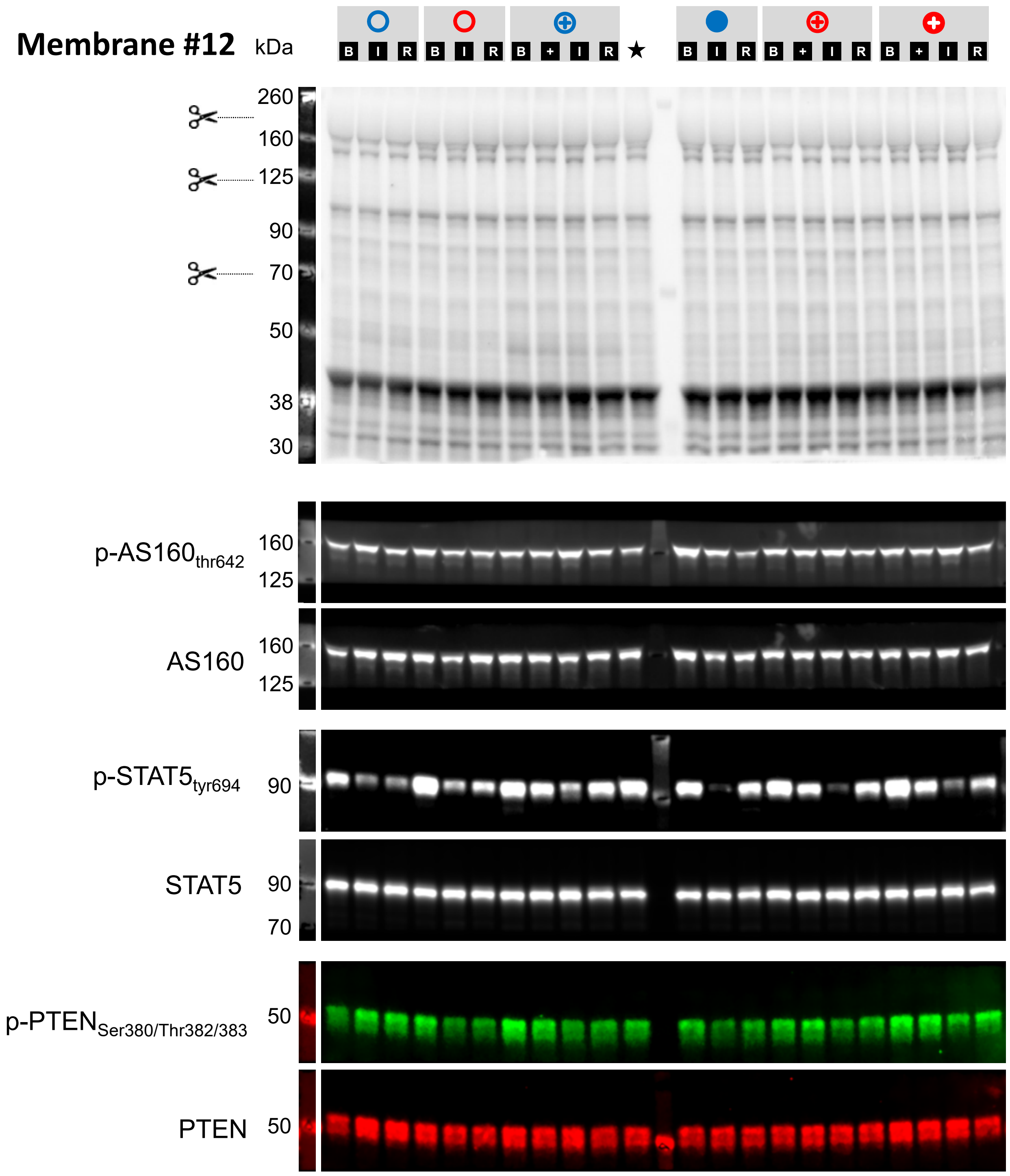
Membrane #12
kDa
B I R B I R B + I R ★ B I R B + I R B + I R
260
160
125
90
70
50
38
30
p-AS160thr642
160
125
AS160
160
125
p-STAT5tyr694
90
STAT5
90
70
p-PTENSer380/Thr382/383
50
PTEN
50

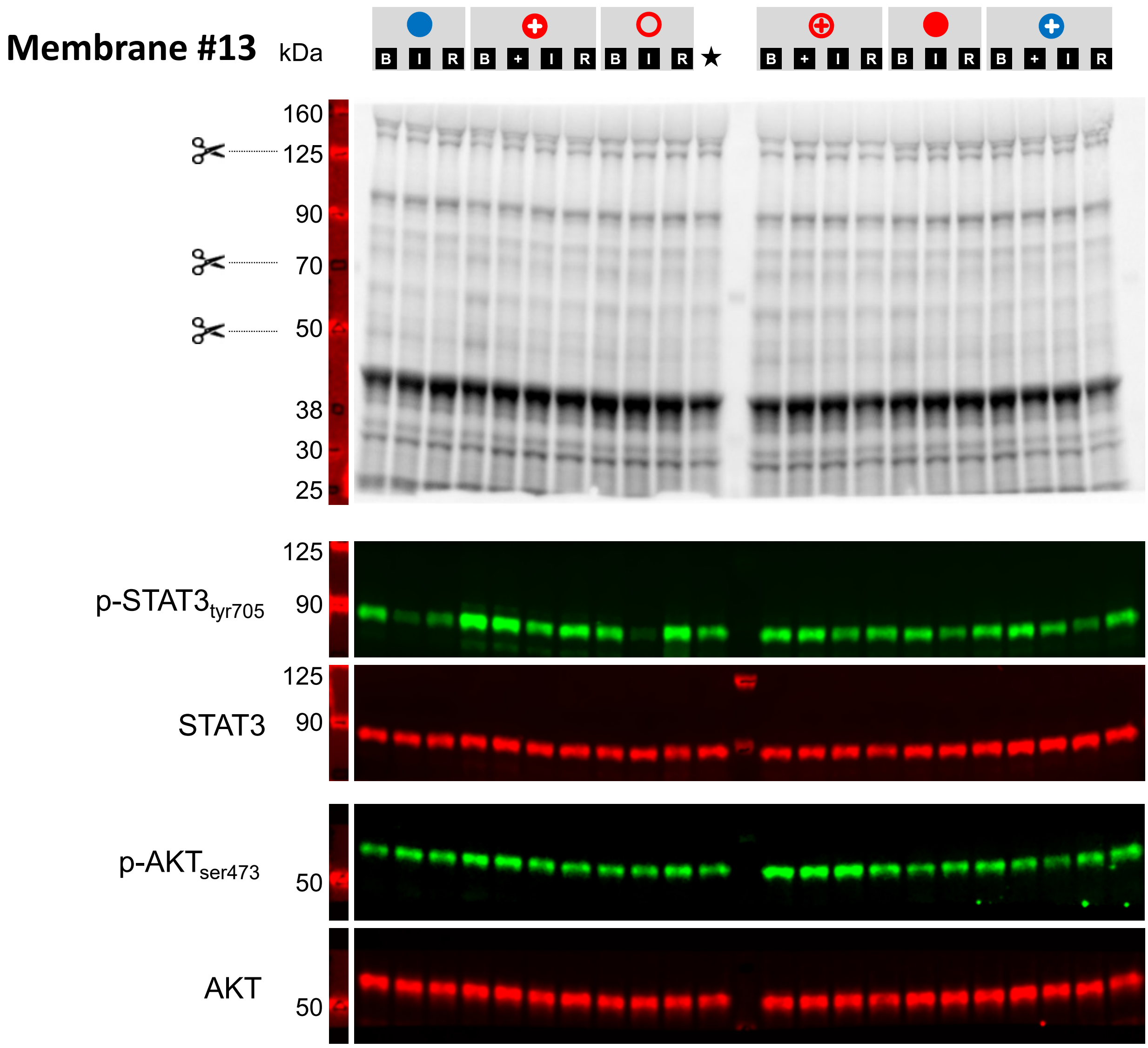
Membrane #13
kDa
B I R B + I R B I R ★ B + I R B I R B + I R
160
125
90
70
50
38
30
25
p-STAT3tyr705
125
90
STAT3
125
90
p-AKTser473
50
AKT
50

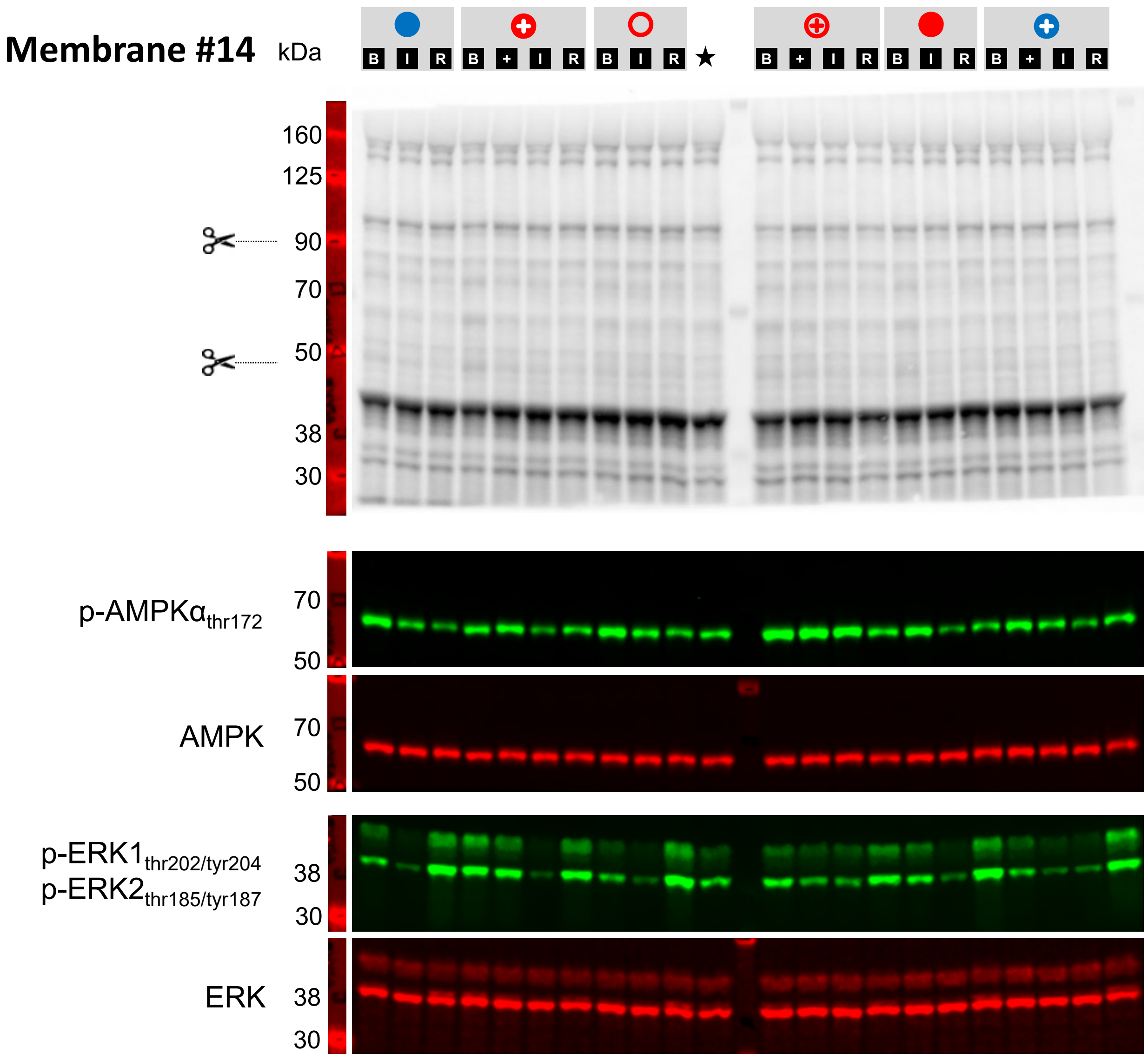
Membrane #14
kDa
B I R B + I R B I R ★ B + I R B I R B + I R
160
125
90
70
50
38
30
p-AMPKα thr172
70
50
AMPK
70
50
p-ERK1 thr202/tyr204
p-ERK2 thr185/tyr187
38
30
ERK
38
30

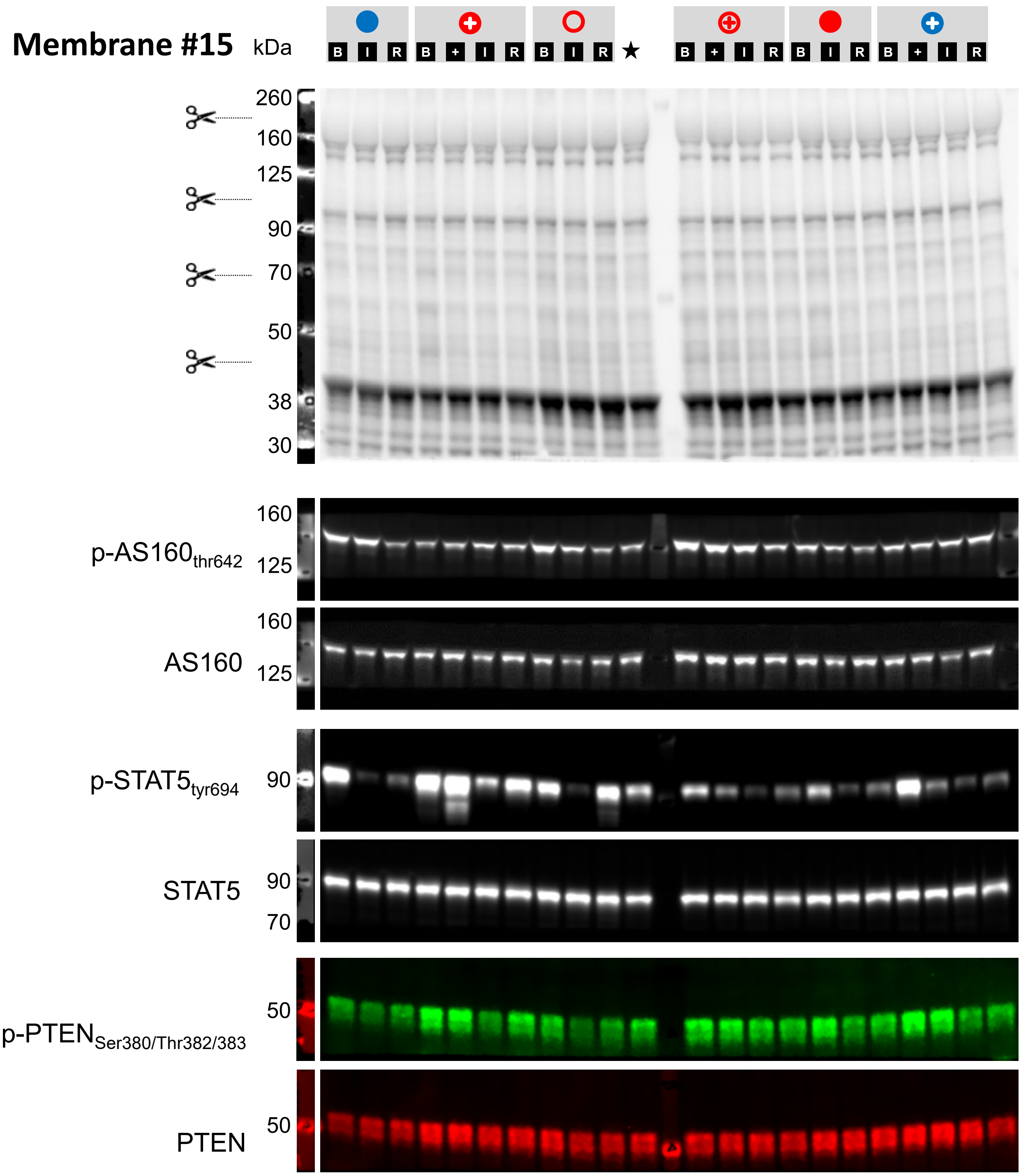
Membrane #15
kDa
B I R B + I R B I R ★ B + I R B I R B + I R
260
160
125
90
70
50
38
30
p-AS160thr642
160
125
AS160
160
125
p-STAT5tyr694
90
STAT5
90
70
p-PTENSer380/Thr382/383
50
PTEN
50

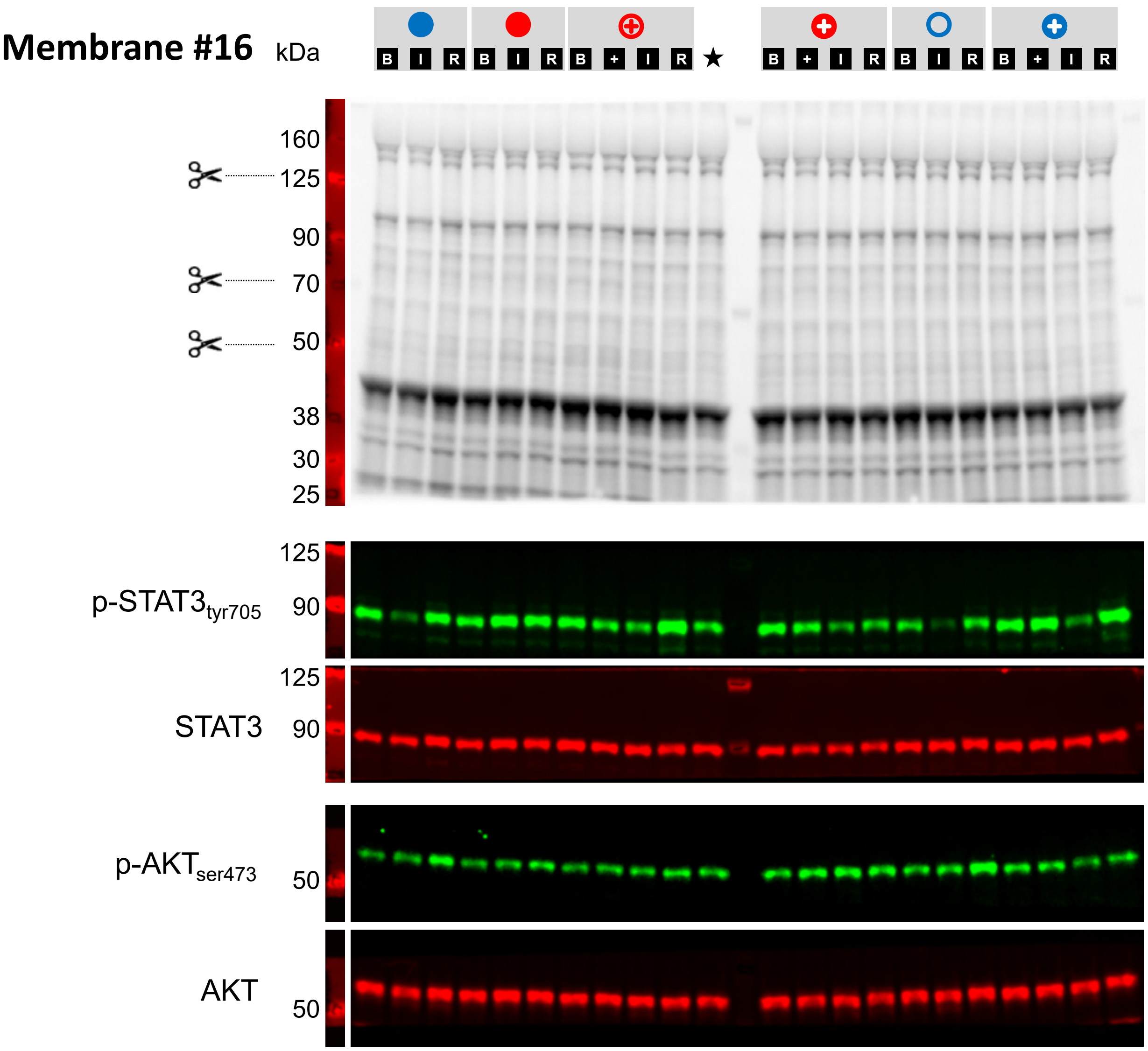
Membrane #16
kDa
B I R B I R B + I R ★ B + I R B I R B + I R
160
125
90
70
50
38
30
25
p-STAT3tyr705
125
90
STAT3
125
90
p-AKTser473
50
AKT
50

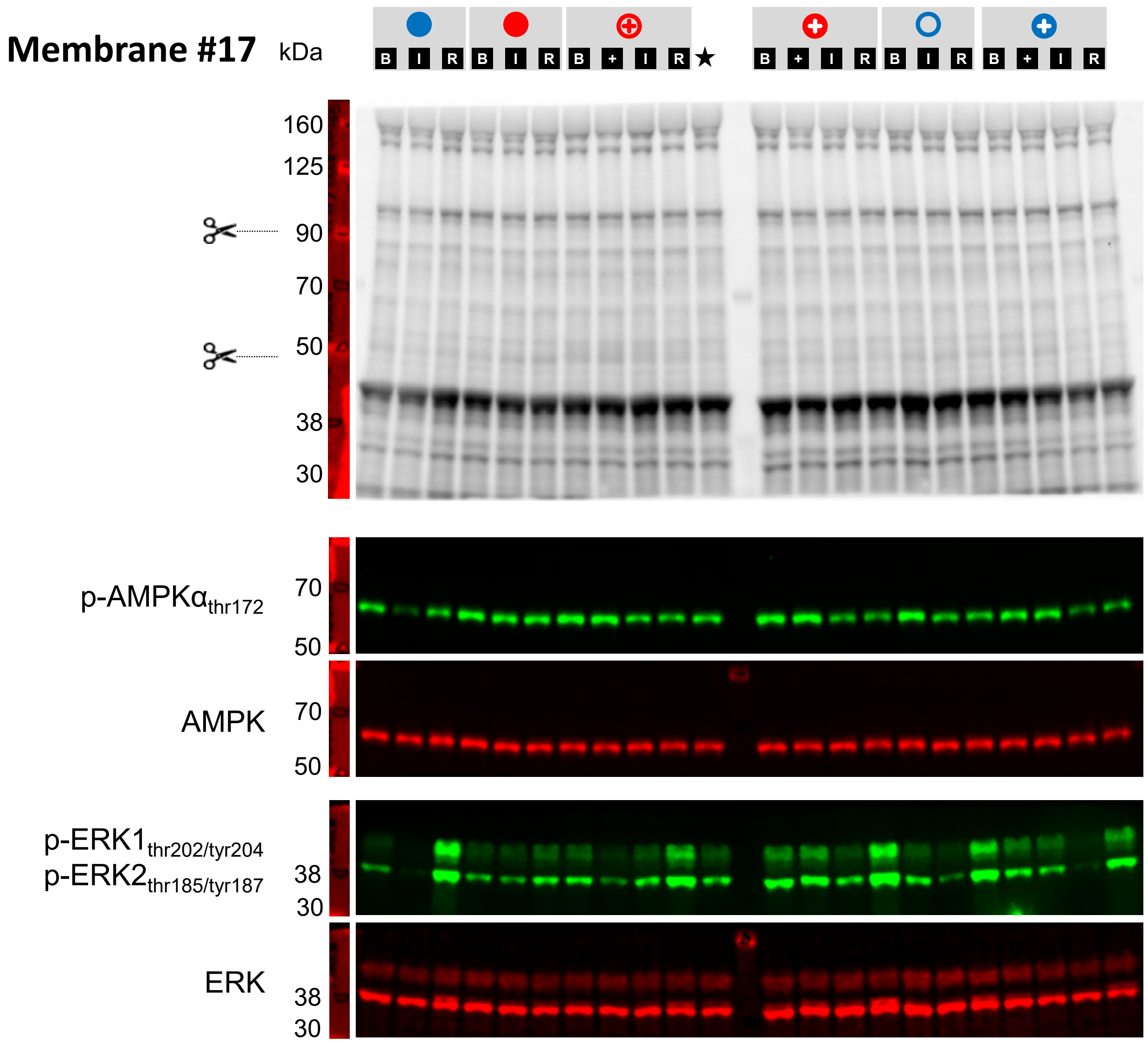

Membrane #17
kDa
B I R B I R B + I R ★ B + I R B I R B + I R
160
125
90
70
50
38
30
p-AMPKα thr172
70
50
AMPK
70
50
p-ERK1 thr202/tyr204
p-ERK2 thr185/tyr187
38
30
ERK
38
30

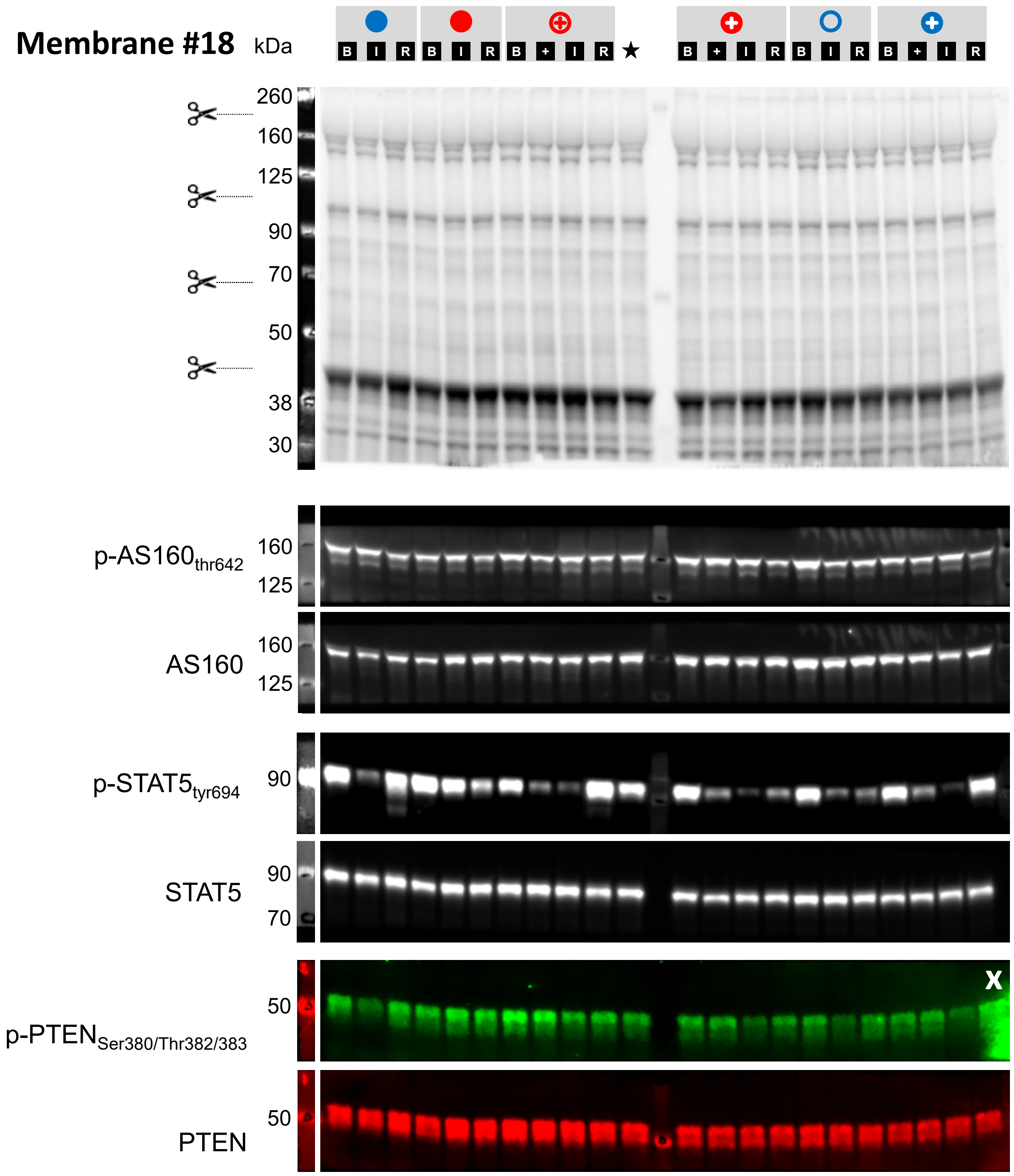

Membrane #18
kDa
B I R B I R B + I R ★ B + I R B I R B + I R
260
160
125
90
70
50
38
30
p-AS160thr642
160
125
AS160
160
125
p-STAT5tyr694
90
STAT5
90
70
p-PTENSer380/Thr382/383
50
X
PTEN
50

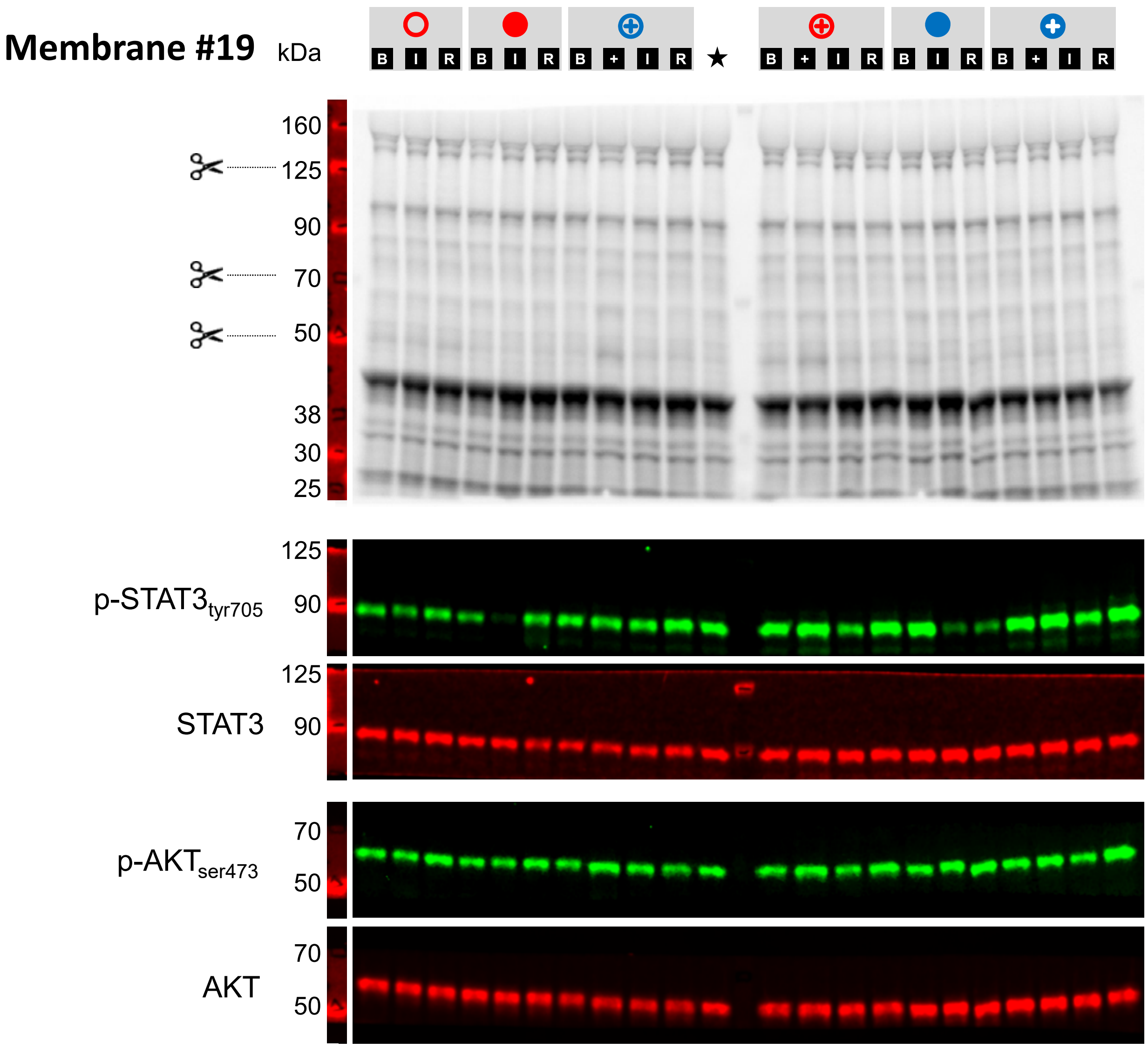
Membrane #19
kDa
B I R B I R B + I R ★ B + I R B I R B + I R
160
125
90
70
50
38
30
25
p-STAT3tyr705
125
90
STAT3
125
90
p-AKTser473
70
50
AKT
70
50

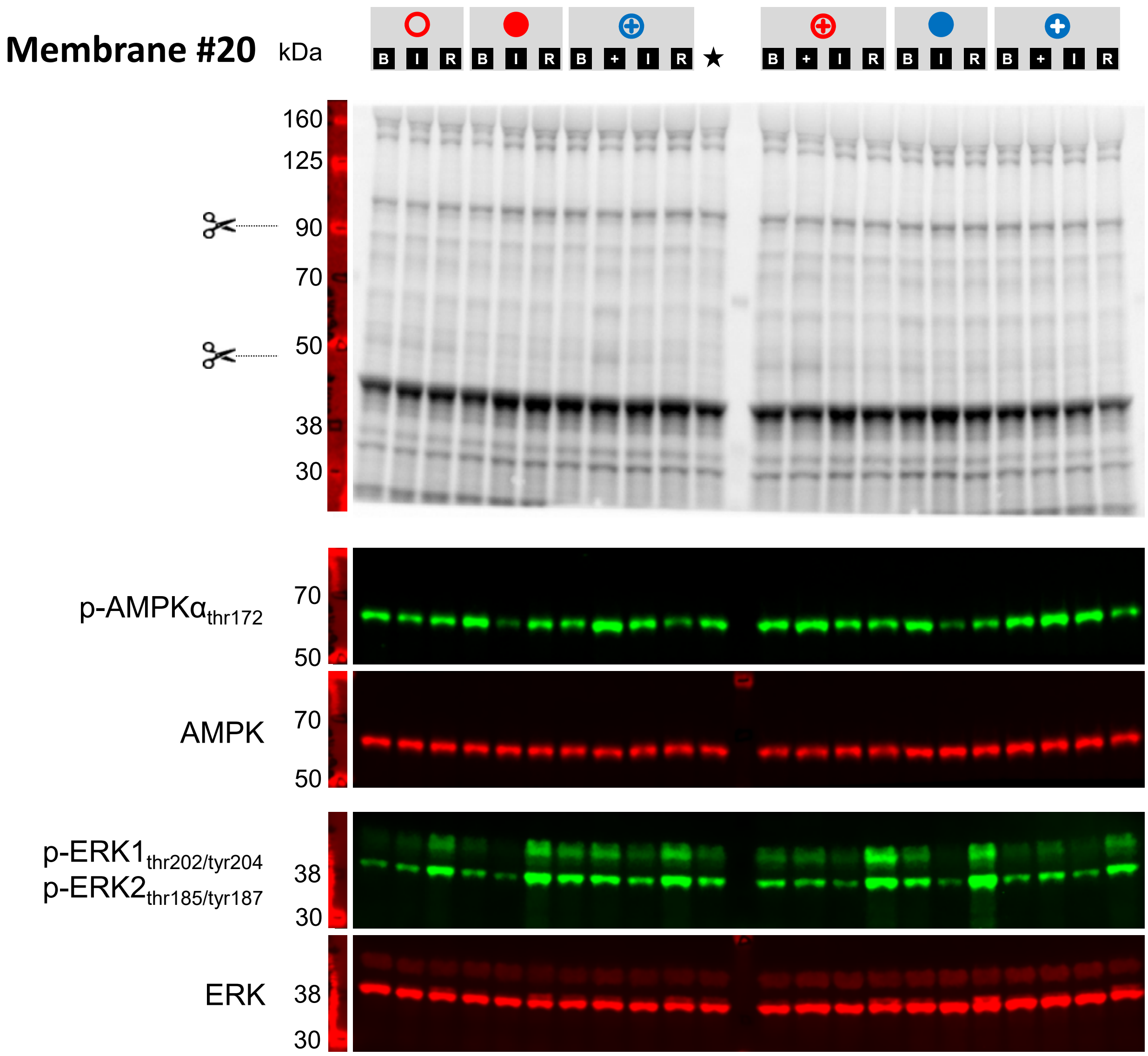

Membrane #20
kDa
B I R B I R B + I R ★ B + I R B I R B + I R
160
125
90
70
50
38
30
p-AMPKα thr172
70
50
AMPK
70
50
p-ERK1 thr202/tyr204
p-ERK2 thr185/tyr187
38
30
ERK
38
30

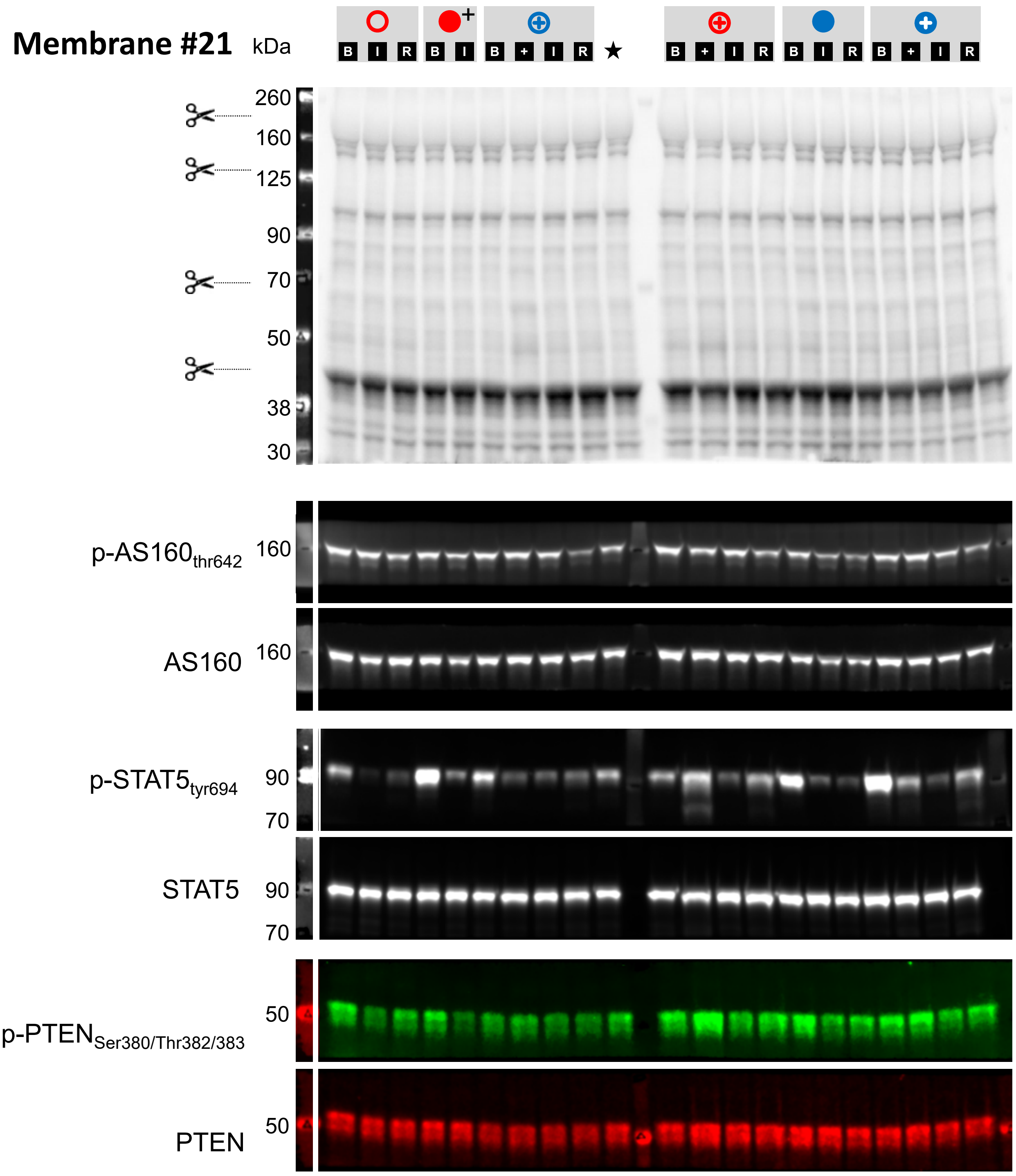
Membrane #21
kDa
B I R B I B + I R ★ B + I R B I R B + I R
260
160
125
90
70
50
38
30
p-AS160thr642
160
AS160
160
p-STAT5tyr694
90
70
STAT5
90
70
p-PTENSer380/Thr382/383
50
PTEN
50

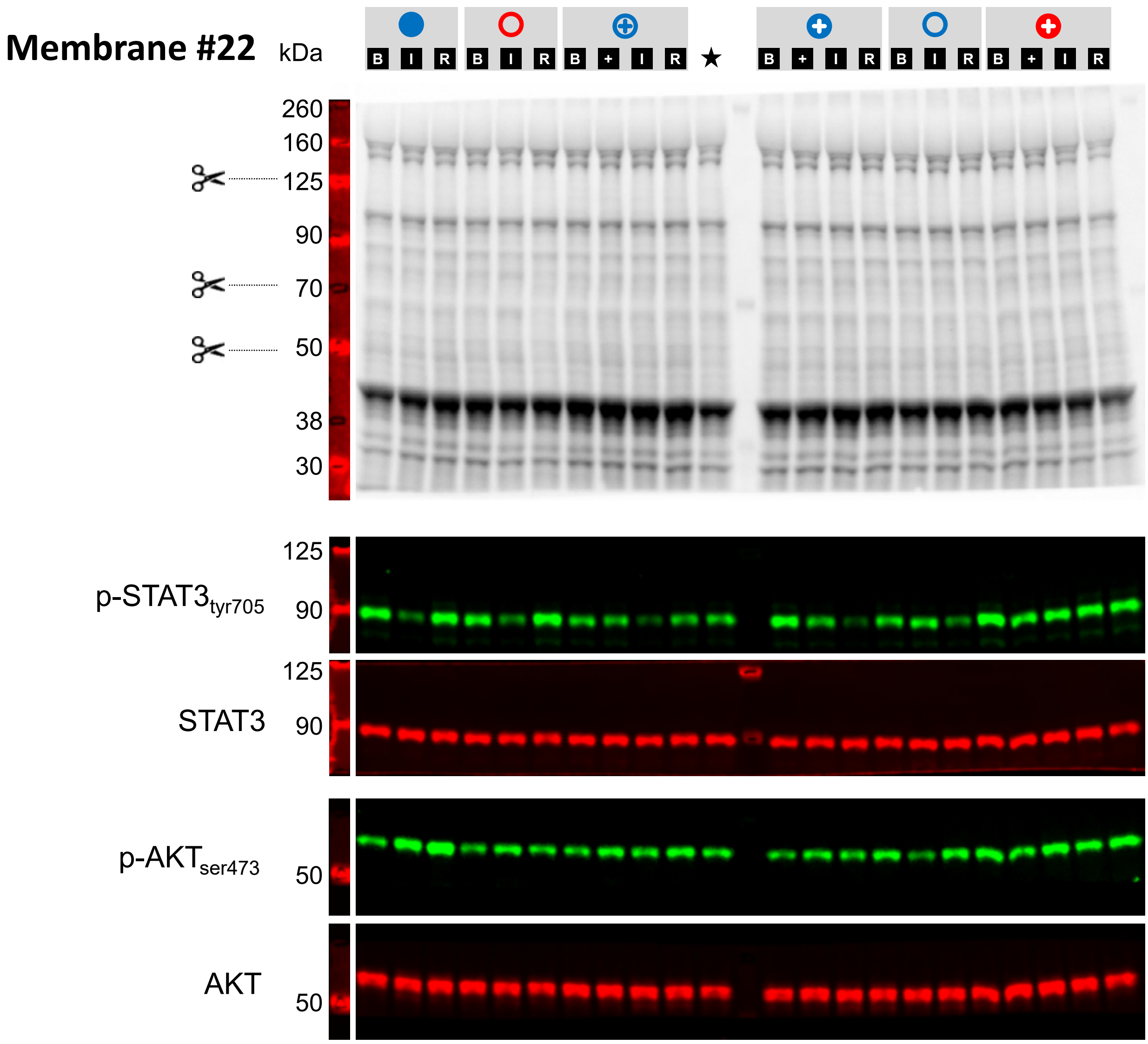
Membrane #22
kDa
B I R B I R B + I R ★ B + I R B I R B + I R
260
160
125
90
70
50
38
30
p-STAT3tyr705
125
90
STAT3
125
90
p-AKTser473
50
AKT
50

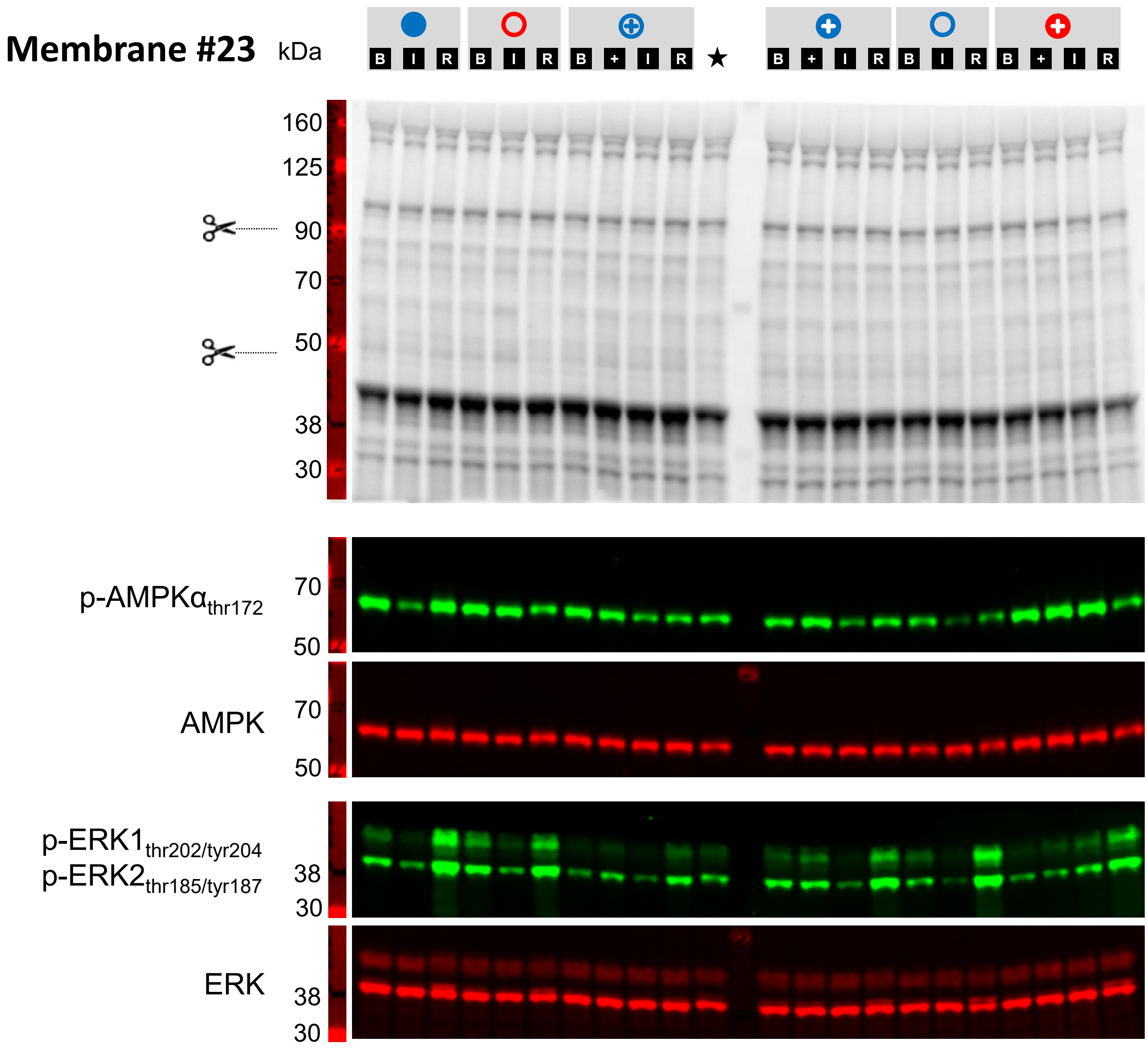
Membrane #23
kDa
B I R B I R B + I R ★ B + I R B I R B + I R
160
125
90
70
50
38
30
p-AMPKα thr172
70
50
AMPK
70
50
p-ERK1 thr202/tyr204
p-ERK2 thr185/tyr187
38
30
ERK
38
30

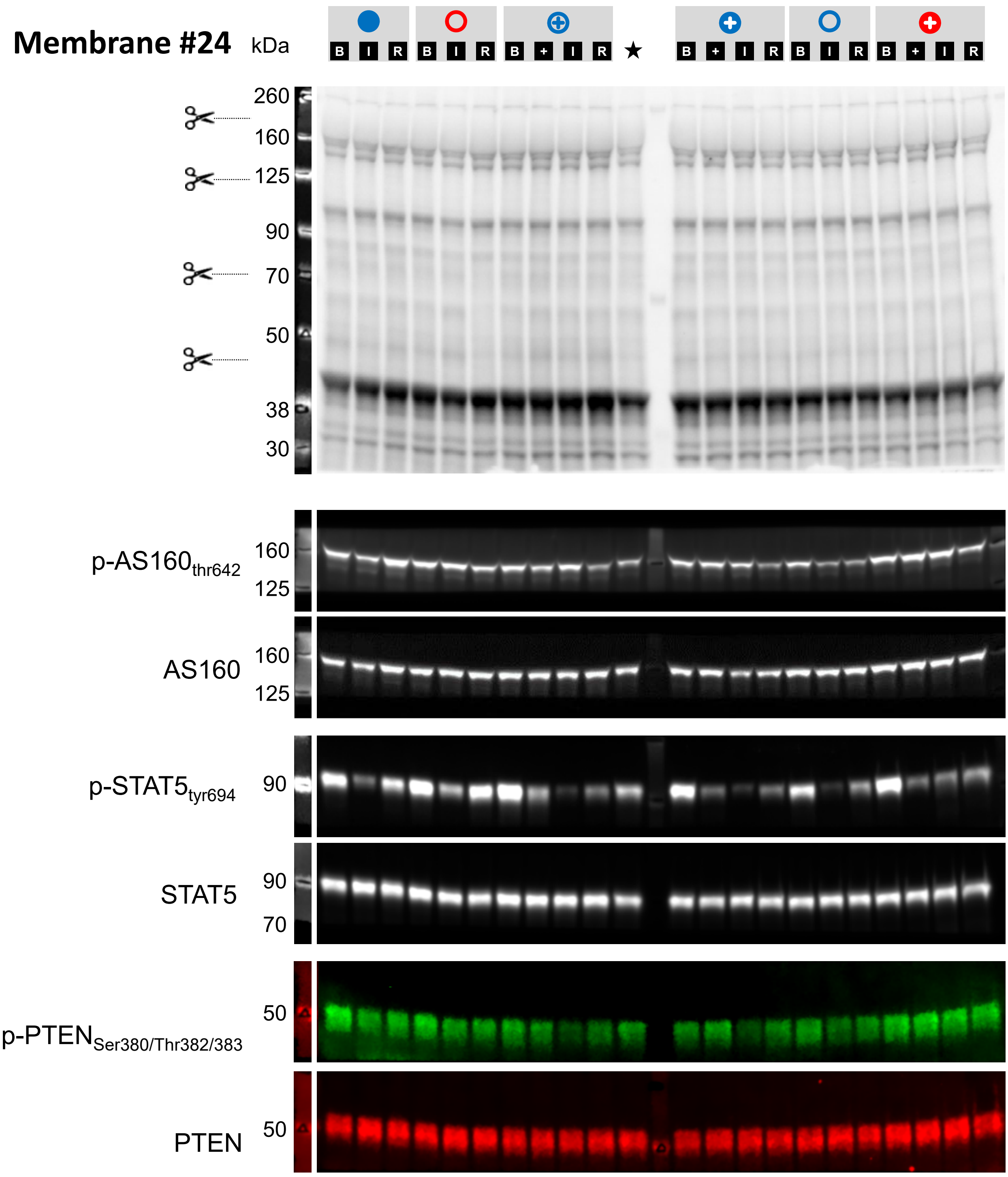

Membrane #24
kDa
B I R B I R B + I R ★ B + I R B I R B + I R
260
160
125
90
70
50
38
30
p-AS160thr642
AS160
p-STAT5tyr694
STAT5
p-PTENSer380/Thr382/383
PTEN

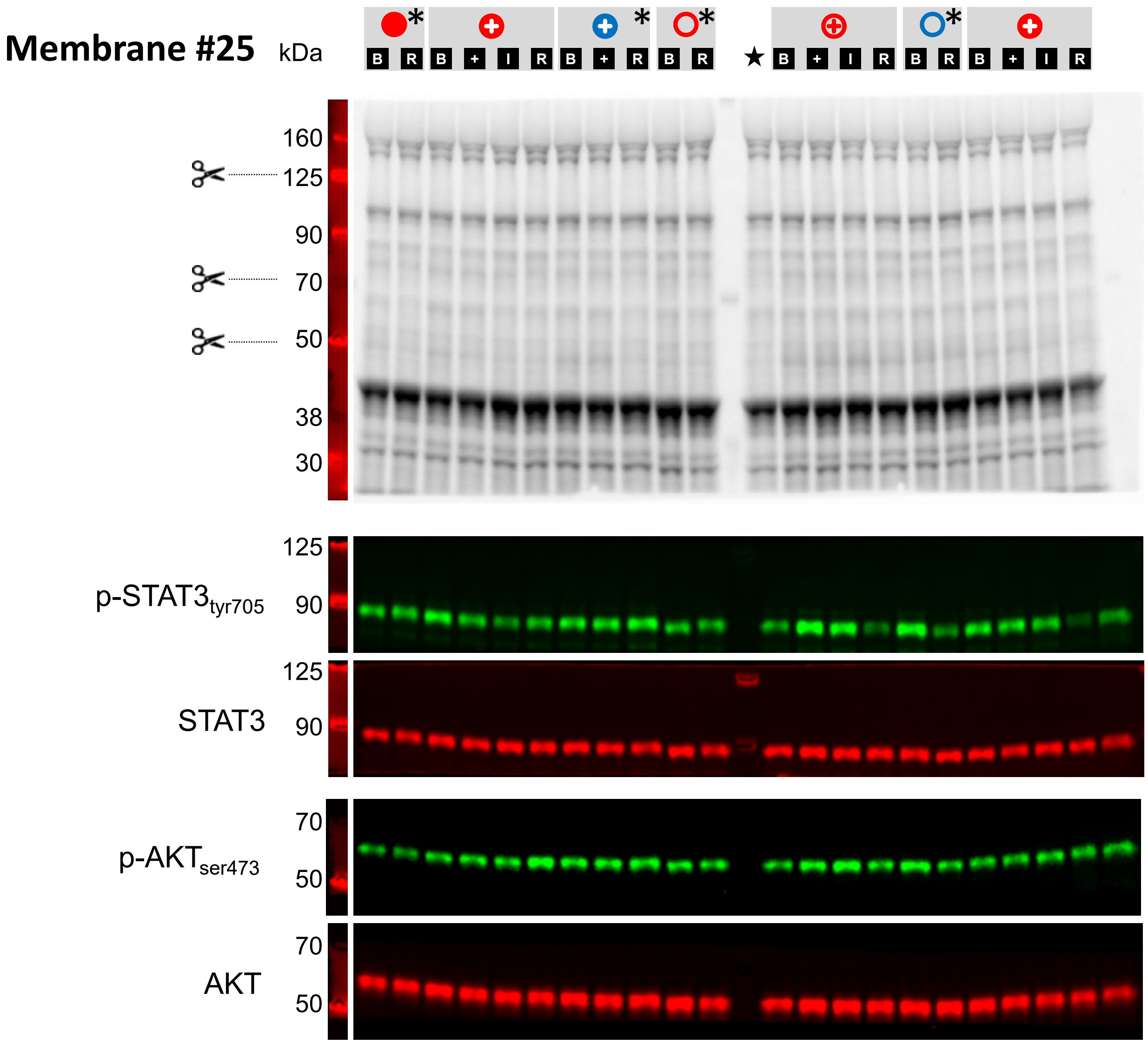
Membrane #25
kDa
B R B + I R B + R B R
B + I R B R B + I R
160
125
90
70
50
38
30
p-STAT3tyr705
125
90
STAT3
125
90
p-AKTser473
70
50
AKT
70
50

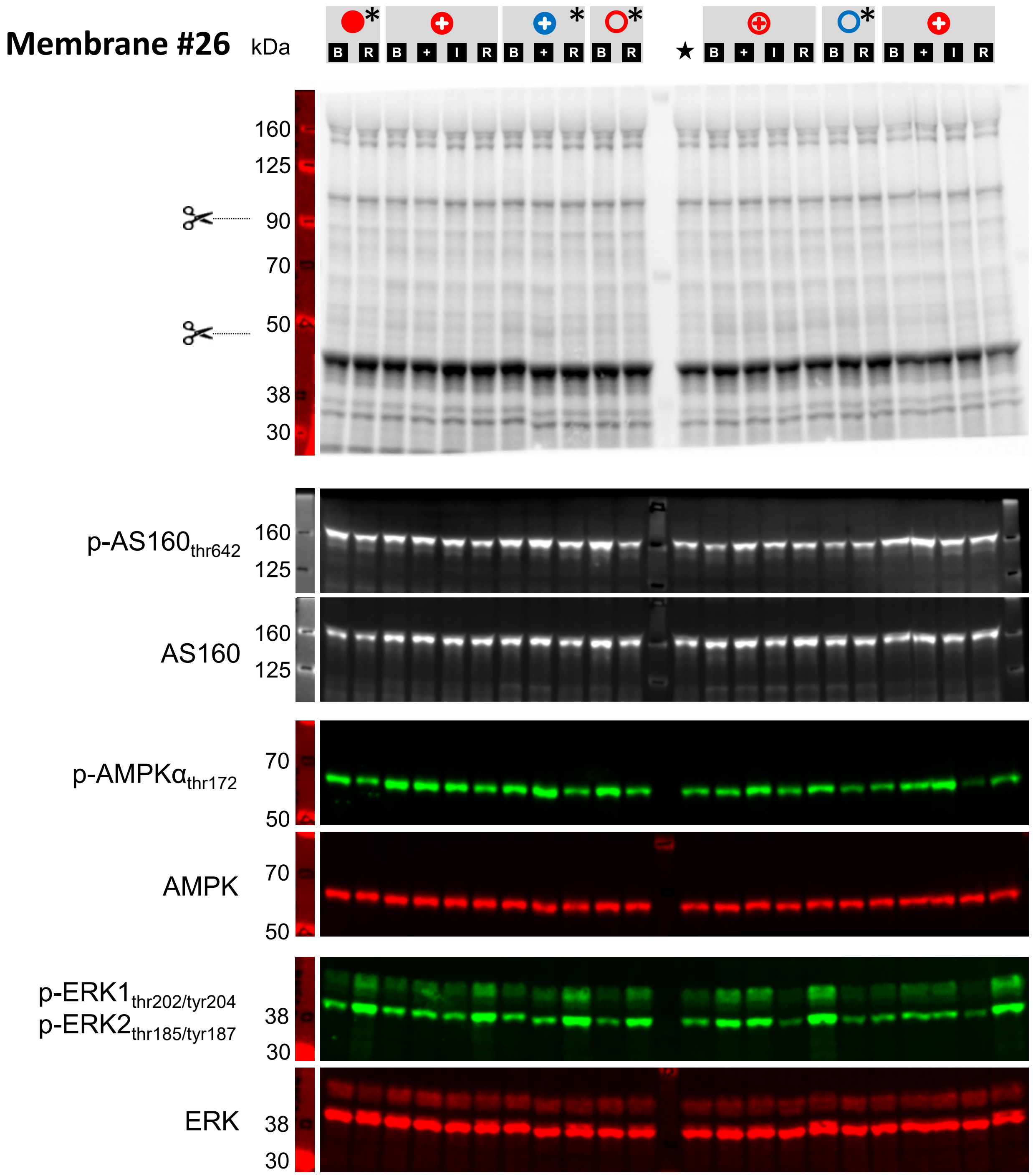

Membrane #26
kDa
B R B + I R B + R B R
B + I R B R B + I R
160
125
90
70
50
38
30
p-AS160$_{thr642}$
AS160
p-AMPKα$_{thr172}$
AMPK
p-ERK1$_{thr202/tyr204}$
p-ERK2$_{thr185/tyr187}$
ERK

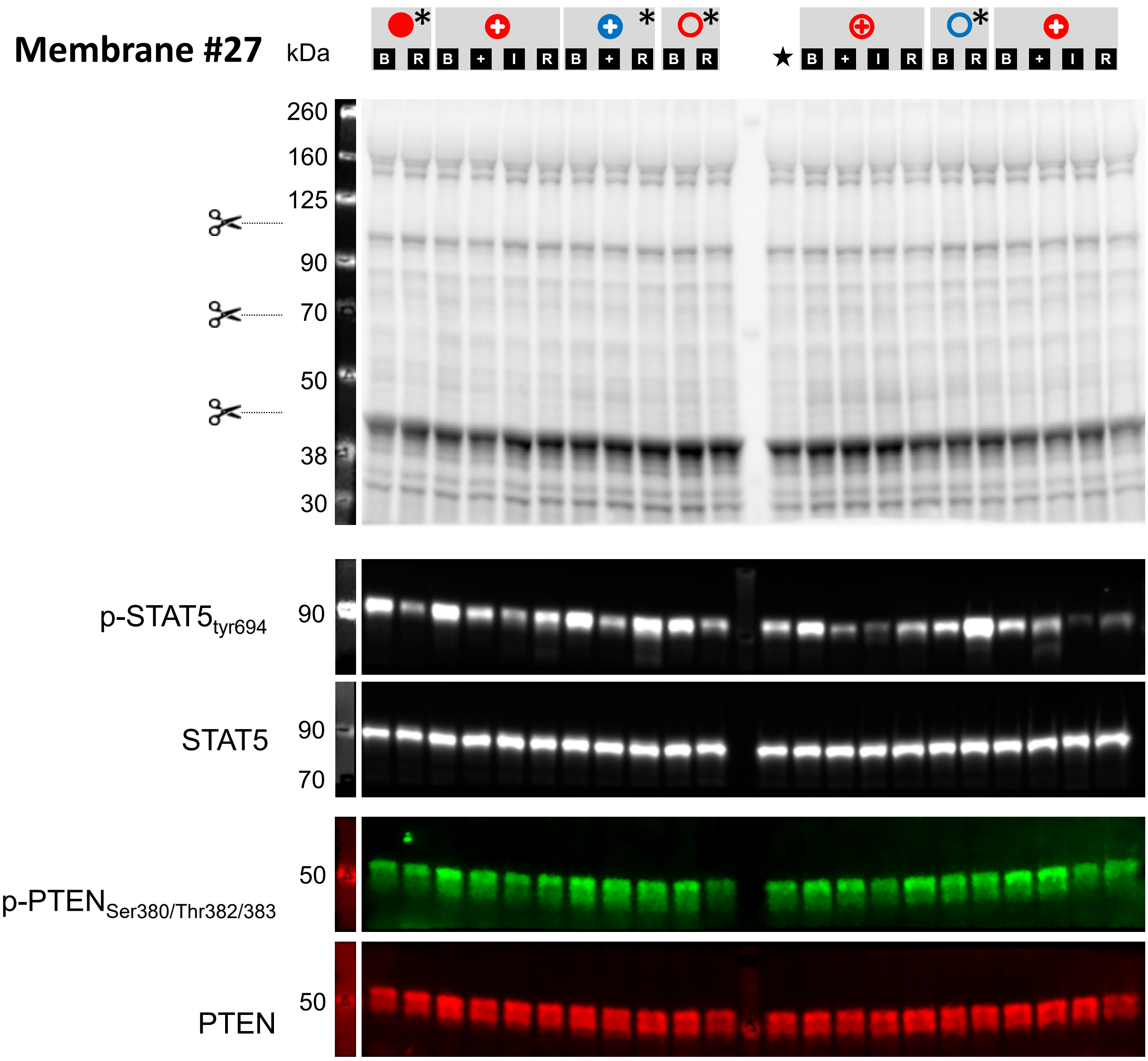
Membrane #27
kDa
B R B + I R B + R B R
★ B + I R B R B + I R
260
160
125
90
70
50
38
30
p-STAT5$_{tyr694}$
90
STAT5
90
70
p-PTEN$_{Ser380/Thr382/383}$
50
PTEN
50

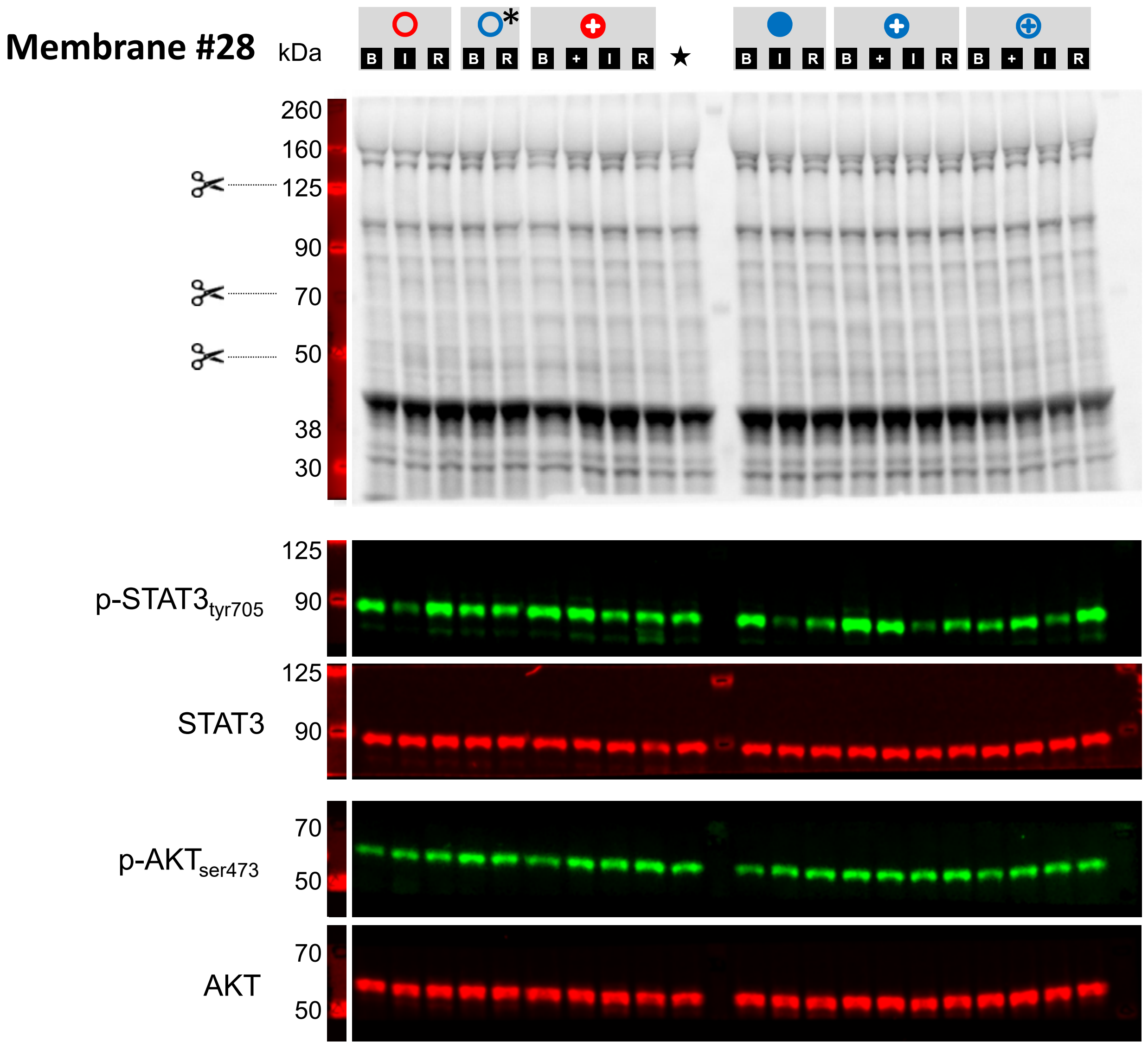
Membrane #28
kDa
B I R B R B + I R ★ B I R B + I R B + I R
260
160
125
90
70
50
38
30
p-STAT3tyr705
125
90
STAT3
125
90
p-AKTser473
70
50
AKT
70
50

Membrane #29

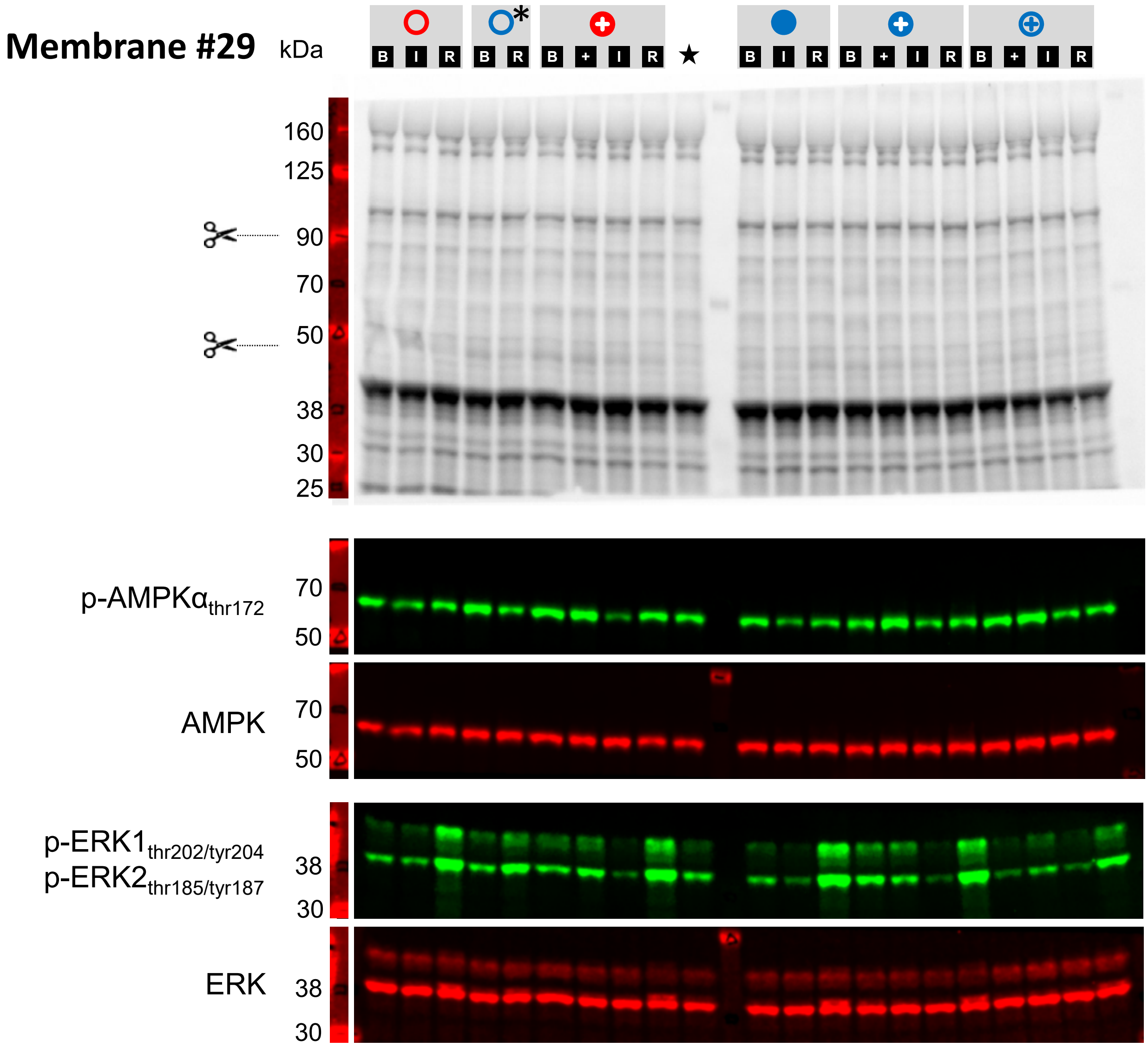
kDa
B I R B R B + I R ★ B I R B + I R B + I R
160
125
90
70
50
38
30
25
p-AMPKα thr172
70
50
AMPK
70
50
p-ERK1 thr202/tyr204
p-ERK2 thr185/tyr187
38
30
ERK
38
30

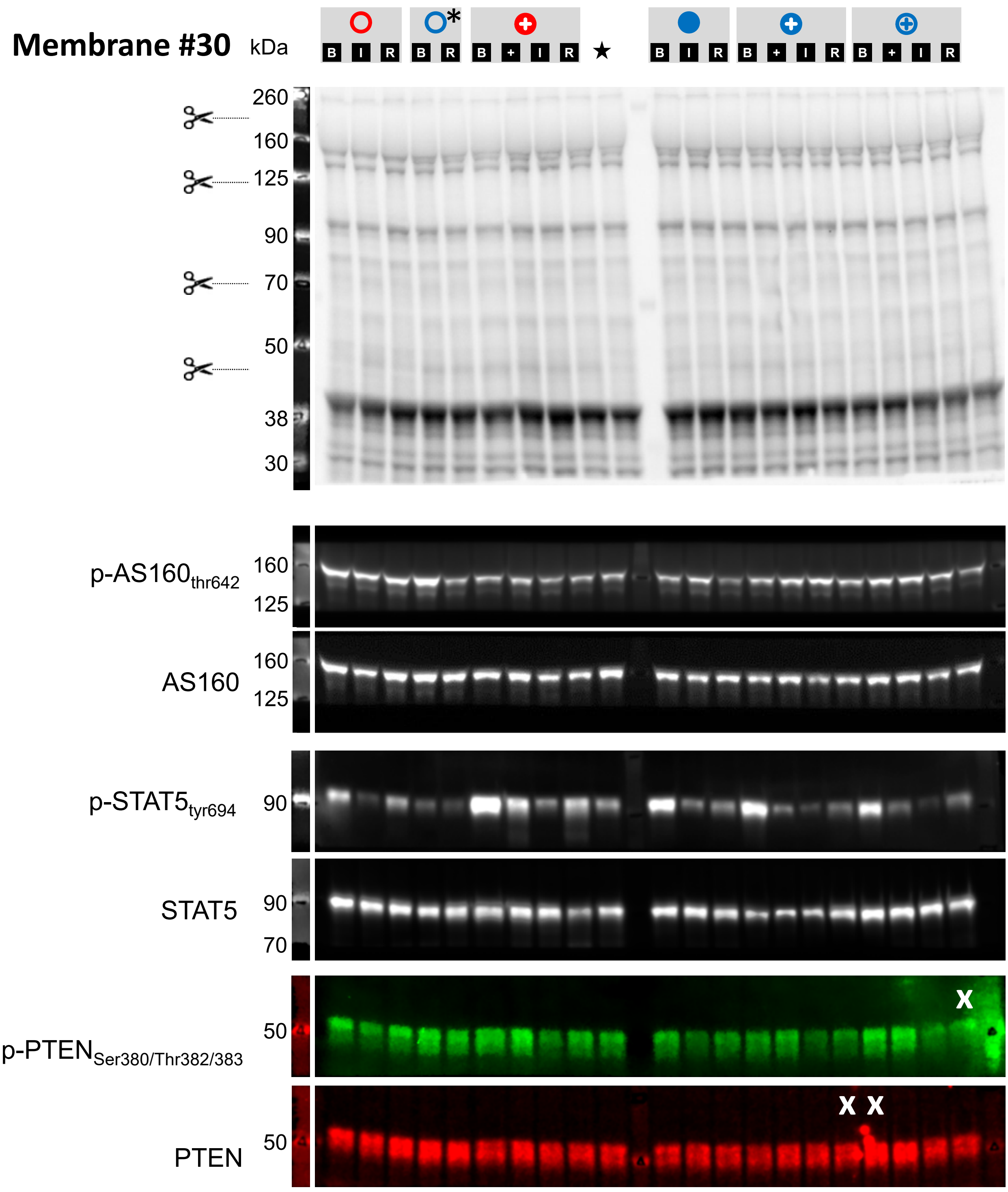
Membrane #30
kDa
B I R B R B + I R
B I R B + I R B + I R
260
160
125
90
70
50
38
30
p-AS160thr642
160
125
AS160
160
125
p-STAT5tyr694
90
STAT5
90
70
p-PTENSer380/Thr382/383
50
PTEN
50
X
X X
